# Supplementary material for: Association analyses identify 31 new risk loci for colorectal cancer susceptibility
Source: Nat Commun. 2019 May 14;10:2154. doi: 10.1038/s41467-019-09775-w (PMC6517433; doi:10.1038/s41467-019-09775-w)
Supplement: Supplementary file 1 — Supplementary Information [file 41467_2019_9775_MOESM1_ESM.pdf]

Supplementary Information: Association analyses identify 31 new risk loci for colorectal cancer susceptibility

Law *et al.*

SUPPLEMENTARY METHODS

Colorectal cancer studies

Genome-wide association studies (GWAS) of colorectal cancer (CRC) have thus far reported 43 SNPs mapping to 40 risk loci in European populations[1-16] (**Supplementary Table 1**). In Asians, 18 SNPs mapping to 16 risk loci have been identified[17-23].

Supplementary tools

Gene expression was normalised using the TMM algorithm, implemented in edgeR[24]. *cis*-eQTL mapping was performed separately for proximal colon, distal colon and rectum samples using FastQTL[25]. Principal components for the SNP data and additional covariate factors were identified using Probabilistic Estimation of Expression Residuals (PEER)[26]. Beta distribution-adjusted empirical *P*-values from FastQTL were used to calculate *Q*-values[27]. MetaTissue[28] was used to generate a "pan-colonic" eQTL measure from the three individual RNA-seq datasets per patient.

Hi-C and CHi-C libraries were sequenced using HiSeq 2000 (Illumina). Reads were aligned to the GRCh37 build using bowtie2 v2.2.6[29] and identification of valid di-tags was performed using HiCUP v0.5.9[30].

ChIP libraries were sequenced using HiSeq 2000 (Illumina) with 100bp single-ended reads. Generated raw reads were filtered for quality (Phred33 ≥ 30) and length (n ≥ 32), and adapter sequences were removed using Trimmomatic v0.22[31]. Reads passing filters were then aligned to the human reference (hg19) using BWA v0.6.1. Peak calls are obtained using MACS2 v 2.0.10.07132012[32].

Chromatin data were obtained from HaploReg v4[33].

Supplementary datasets

Transcription factor binding disruption was performed by tested the SNPs by estimating their effects on over 2,800 binding motifs as characterised by ENCODE[34], FactorBook[35], HOCOMOCO[36] and HOMER[37].

LD score regression was used to determine if any traits were correlated with CRC risk. GWAS summary data was obtained for allergy[38], asthma[38], coronary artery disease[39], fatty acids[40-45], lipids (total cholesterol, high density lipoprotein, low density lipoprotein, triglycerides)[46], auto-immune diseases

(Crohn's disease[47], rheumatoid arthritis[48], atopic dermatitis[49], celiac disease[50], multiple sclerosis[51], primary biliary cirrhosis[52], inflammatory bowel disease[47], ulcerative colitis[47], systemic lupus erythematosus[53]), anthropometric measures (BMI, height, body fat)[54,55], glucose sensitivity (fasting glucose, fasting insulin, HbA1c)[56,57], childhood measures (birth weight[58], birth length[59], childhood obesity[60], childhood BMI[61]), eGFR[62], and type 2 diabetes[63].

SUPPLEMENTARY FIGURES

Supplementary Figure 1: Quantile-Quantile plots of observed and expected χ^2 values of association between SNP genotype and CRC after imputation for (a) NSCCG-OncoArray, (b) SCOT, (c) SOCCS/GS, (d) SOCCS/LBC, (e) UK Biobank, and (f) overall meta-analysis. Meta λ_{GC} = 1.10, λ_{1000} = 1.00. The red line represents the null hypothesis of no true association.

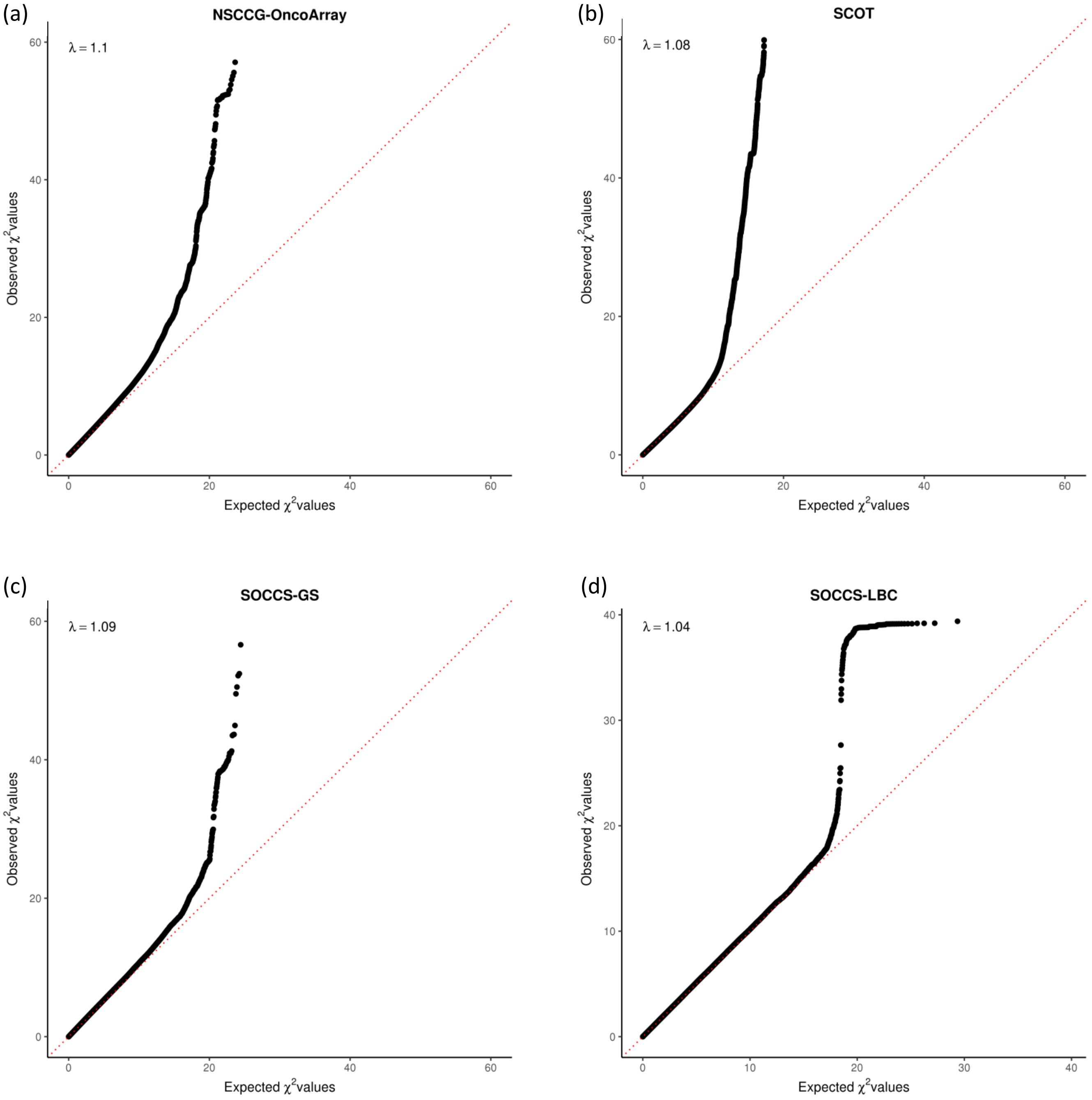

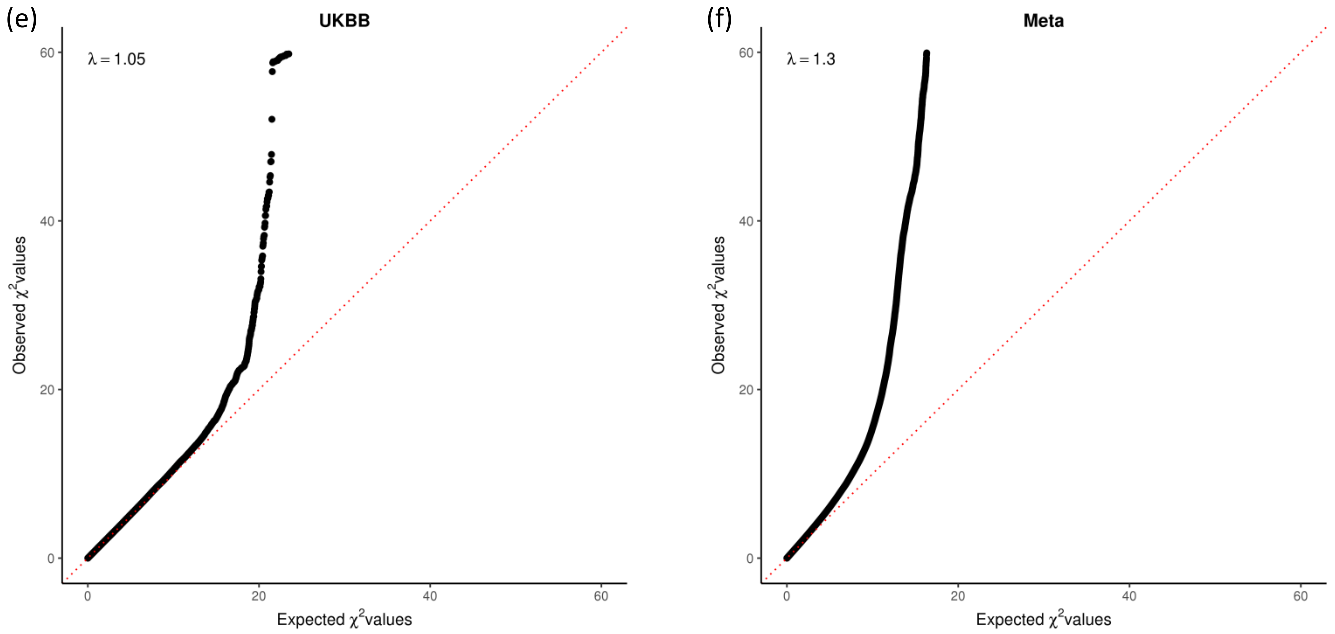
(e)
UKBB
λ = 1.05
Observed χ² values
Expected χ² values
0
20
40
60
(f)
Meta
λ = 1.3
Observed χ² values
Expected χ² values
0
20
40
60

Supplementary Figure 2: Regional plots of the new colorectal cancer risk loci. In the main panel, $-\log_{10}$ *P*-values (y-axis) of the SNPs are shown according to their chromosomal positions (x-axis). The colour intensity of each symbol reflects the extent of LD with the top SNP: white (r^2 = 0) through to dark red (r^2 = 1.0), with r^2 estimated from EUR 1000 Genomes data. Genetic recombination rates (cM/Mb), are shown with a light blue line. Physical positions are based on GRCh37 of the human genome. Where available, the upper panel shows Hi-C contacts from HT29 or LoVo. The lower panel shows the chromatin state segmentation track from the Roadmap epigenomics project (colonic mucosa, rectal mucosa, sigmoid colon), and HCT116. Also shown are the relative positions of genes and transcripts mapping to each region of association.

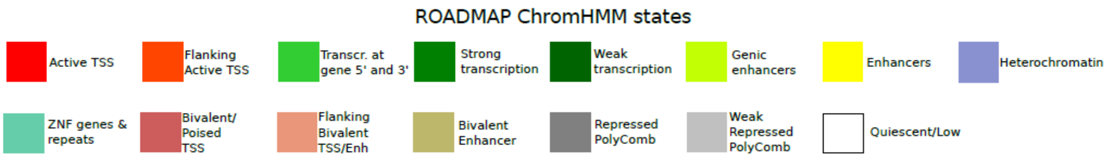

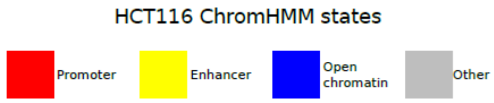

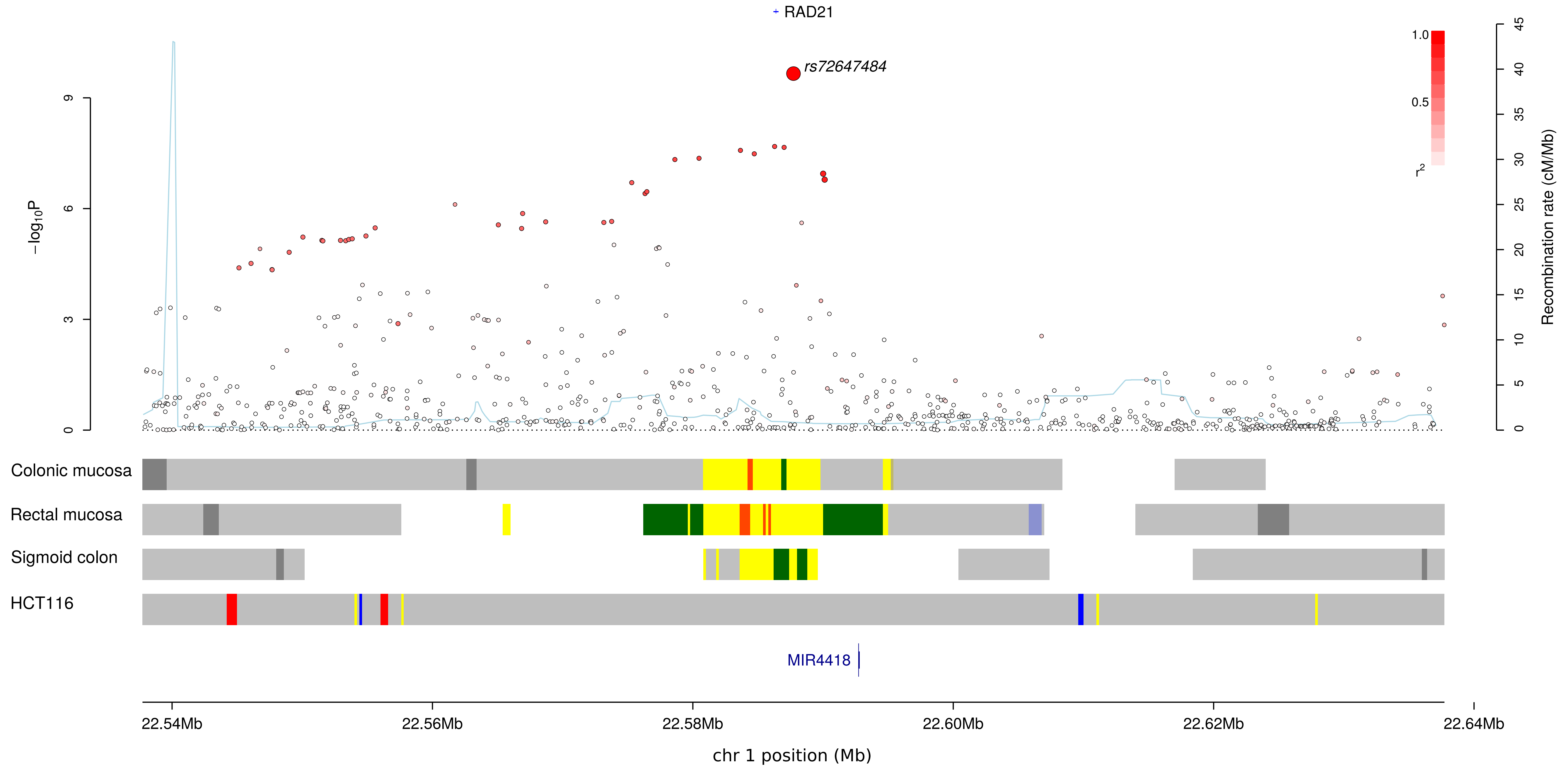

RAD21
rs72647484
1.0
0.5
r^2
$-\log_{10}P$
0
3
6
9
Recombination rate (cM/Mb)
0
5
10
15
20
25
30
35
40
45
Colonic mucosa
Rectal mucosa
Sigmoid colon
HCT116
MIR4418
22.54Mb
22.56Mb
22.58Mb
22.60Mb
22.62Mb
22.64Mb
chr 1 position (Mb)

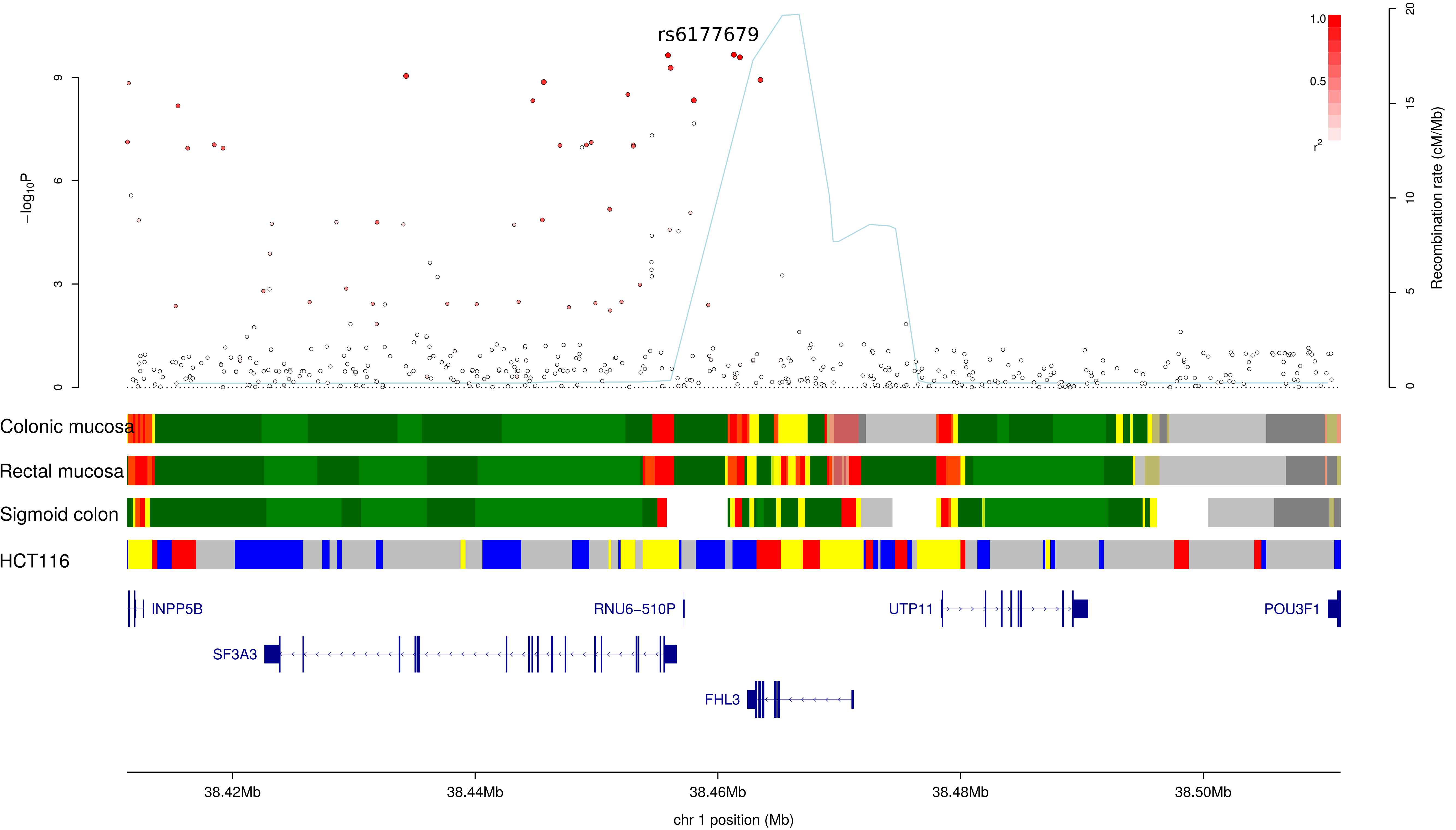
rs6177679
−log10P
0
3
6
9
Recombination rate (cM/Mb)
0
5
10
15
20
1.0
0.5
r2
Colonic mucosa
Rectal mucosa
Sigmoid colon
HCT116
INPP5B
RNU6−510P
UTP11
POU3F1
SF3A3
FHL3
38.42Mb
38.44Mb
38.46Mb
38.48Mb
38.50Mb
chr 1 position (Mb)

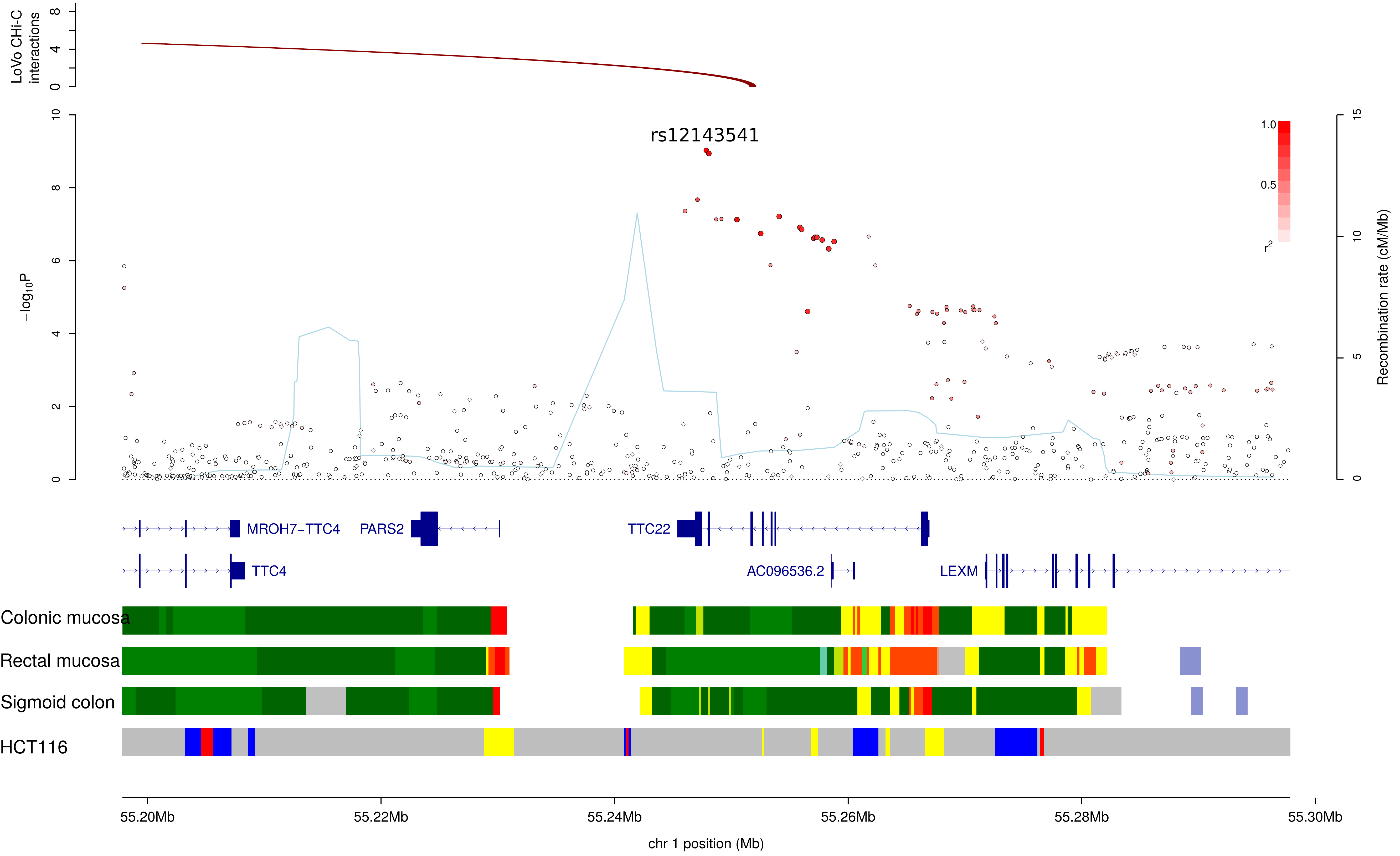
LoVo CHi-C interactions
0
4
8
−log10P
Recombination rate (cM/Mb)
rs12143541
1.0
0.5
r2
MROH7−TTC4
PARS2
TTC22
TTC4
AC096536.2
LEXM
Colonic mucosa
Rectal mucosa
Sigmoid colon
HCT116
55.20Mb
55.22Mb
55.24Mb
55.26Mb
55.28Mb
55.30Mb
chr 1 position (Mb)

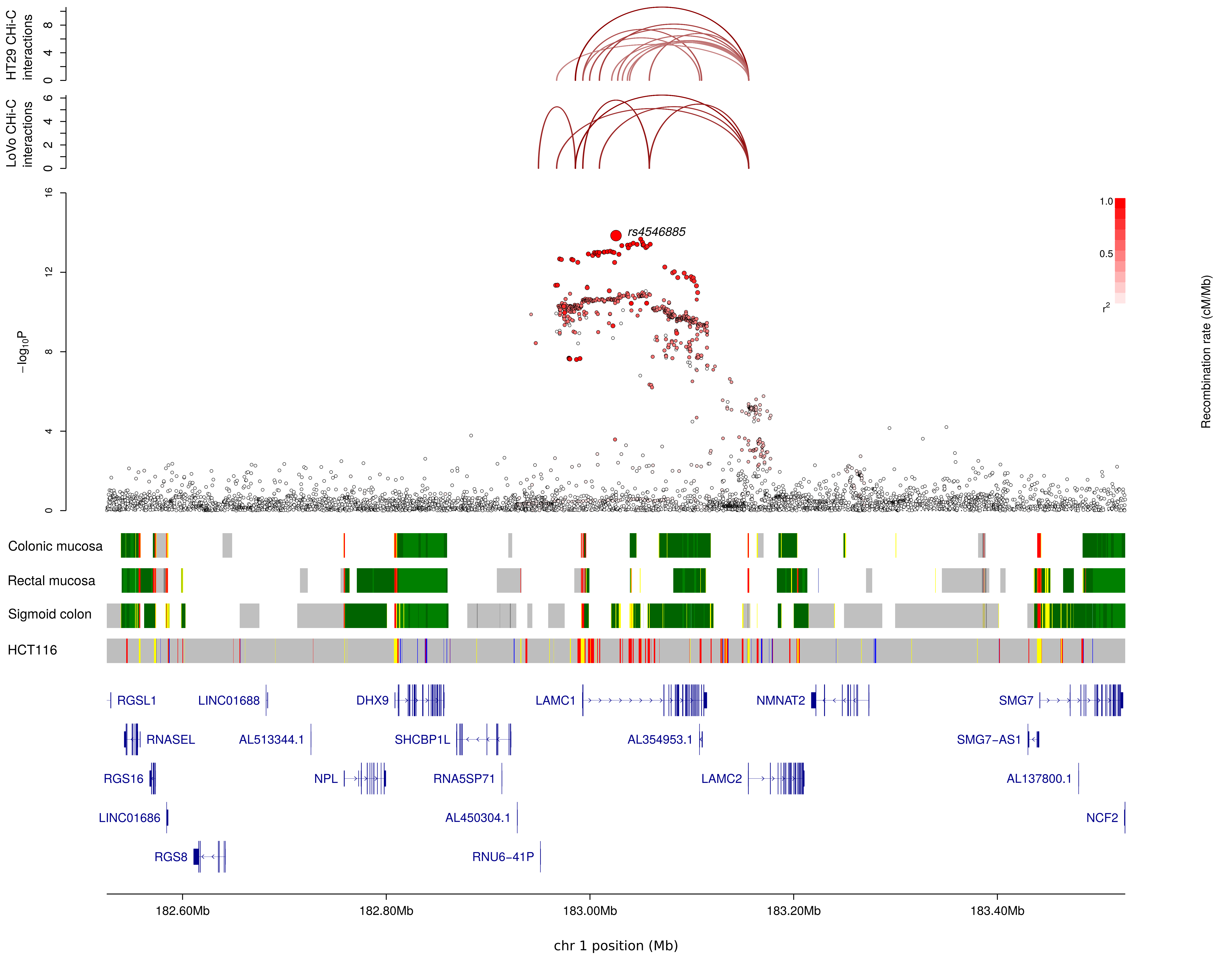

HT29 CHi-C interactions
LoVo CHi-C interactions
−log10P
rs4546885
Recombination rate (cM/Mb)
1.0
0.5
r2
Colonic mucosa
Rectal mucosa
Sigmoid colon
HCT116
RGSL1
LINC01688
DHX9
LAMC1
NMNAT2
SMG7
RNASEL
AL513344.1
SHCBP1L
AL354953.1
SMG7−AS1
RGS16
NPL
RNA5SP71
LAMC2
AL137800.1
LINC01686
AL450304.1
NCF2
RGS8
RNU6−41P
182.60Mb
182.80Mb
183.00Mb
183.20Mb
183.40Mb
chr 1 position (Mb)

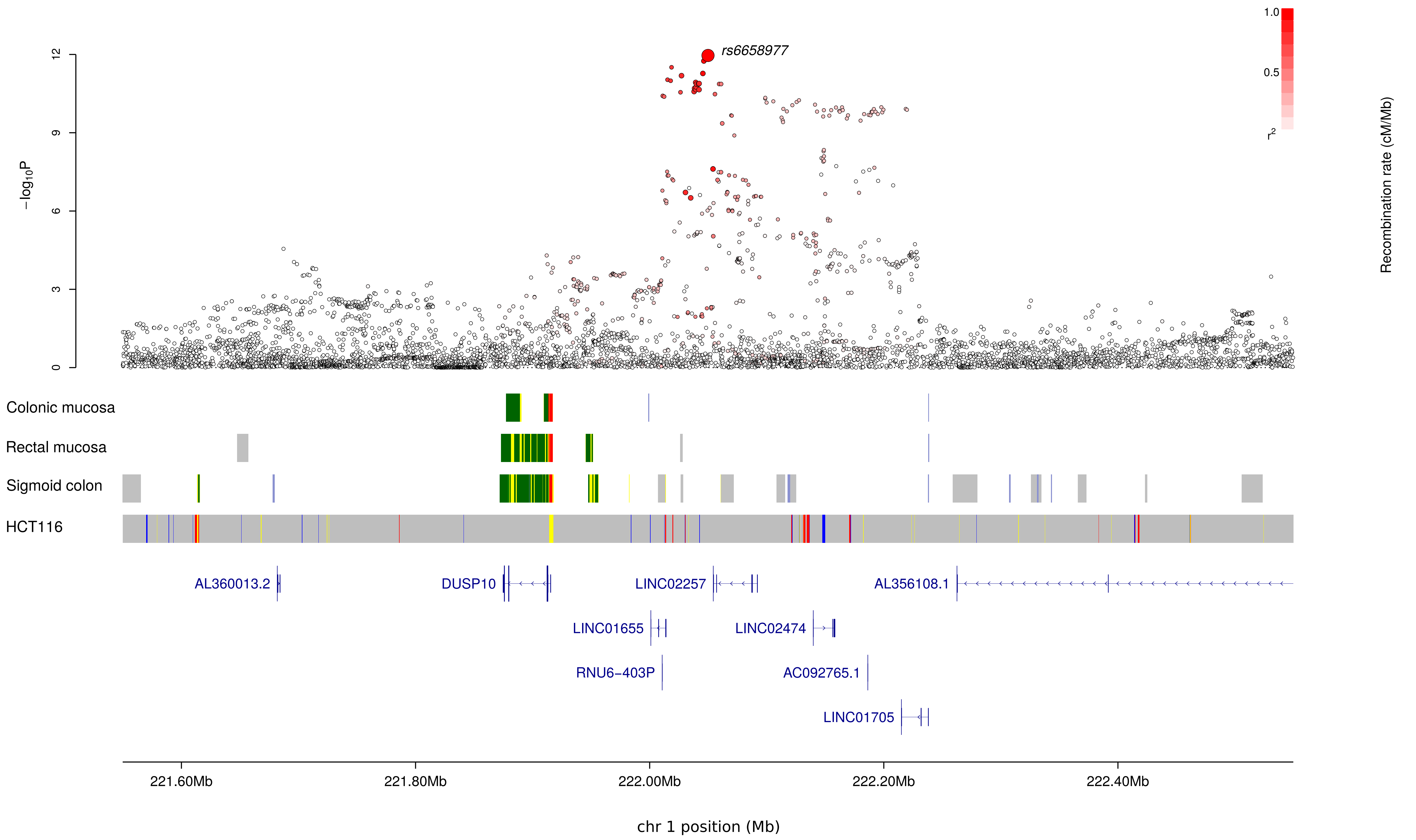

rs6658977
1.0
0.5
r^2
Recombination rate (cM/Mb)
$-\log_{10}P$
12
9
6
3
0
Colonic mucosa
Rectal mucosa
Sigmoid colon
HCT116
AL360013.2
DUSP10
LINC02257
AL356108.1
LINC01655
LINC02474
RNU6−403P
AC092765.1
LINC01705
221.60Mb
221.80Mb
222.00Mb
222.20Mb
222.40Mb
chr 1 position (Mb)

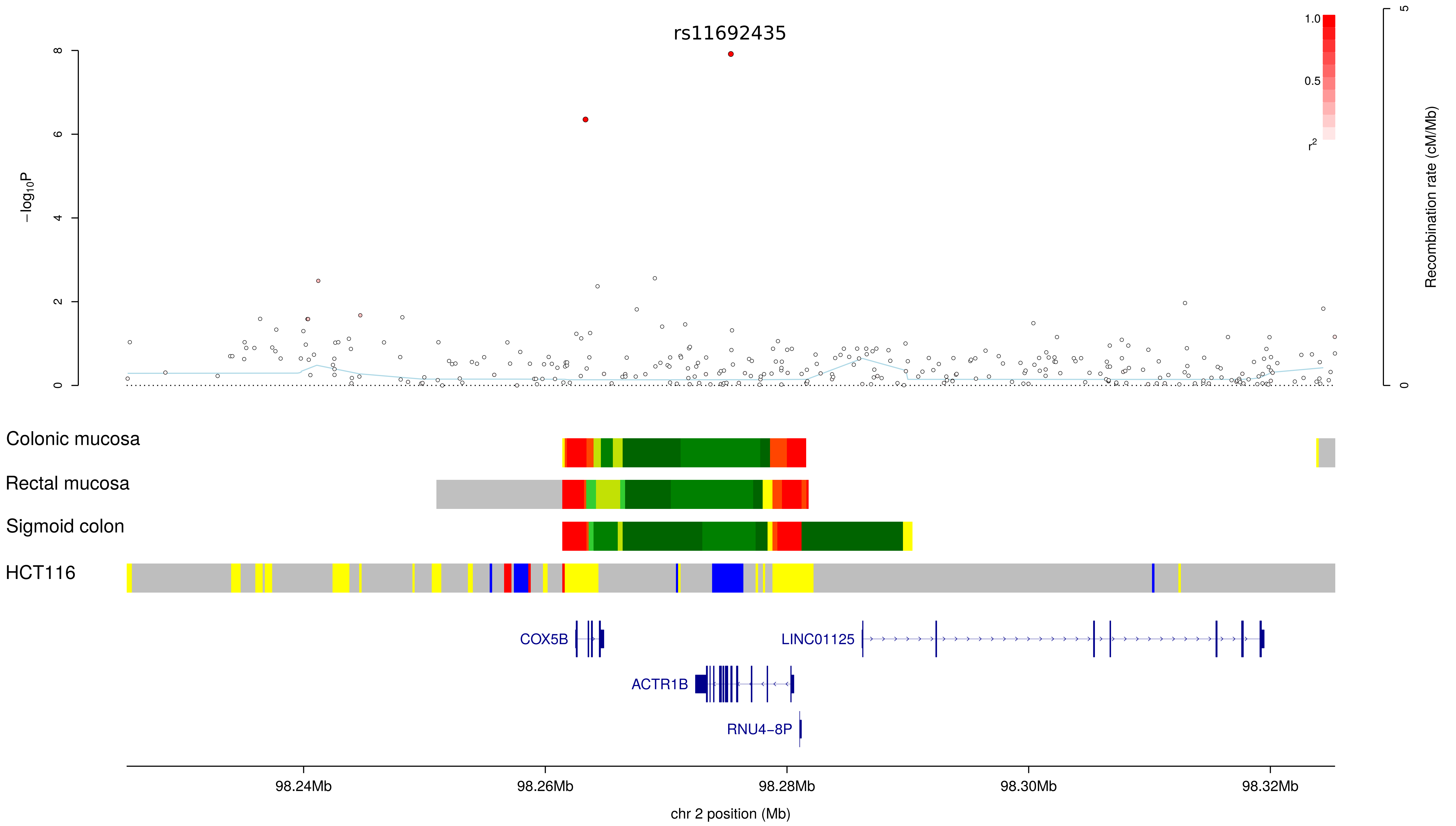

rs11692435
−log10P
Recombination rate (cM/Mb)
1.0
0.5
r2
Colonic mucosa
Rectal mucosa
Sigmoid colon
HCT116
COX5B
LINC01125
ACTR1B
RNU4−8P
98.24Mb
98.26Mb
98.28Mb
98.30Mb
98.32Mb
chr 2 position (Mb)

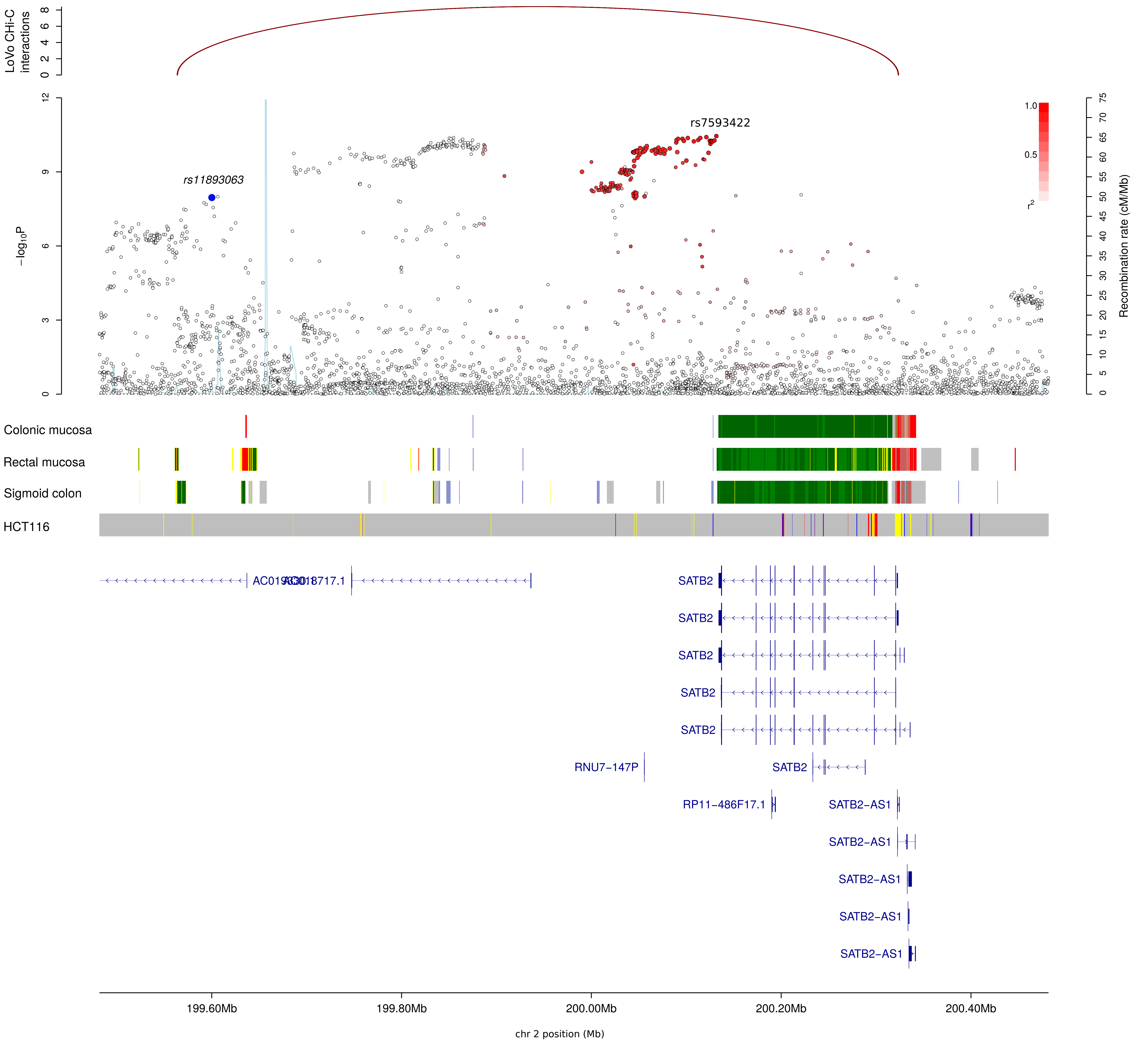

LoVo CHi-C interactions
−log10P
Recombination rate (cM/Mb)
rs7593422
rs11893063
r2
Colonic mucosa
Rectal mucosa
Sigmoid colon
HCT116
AC019130.1
AC018717.1
SATB2
RNU7−147P
RP11−486F17.1
SATB2−AS1
199.60Mb
199.80Mb
200.00Mb
200.20Mb
200.40Mb
chr 2 position (Mb)

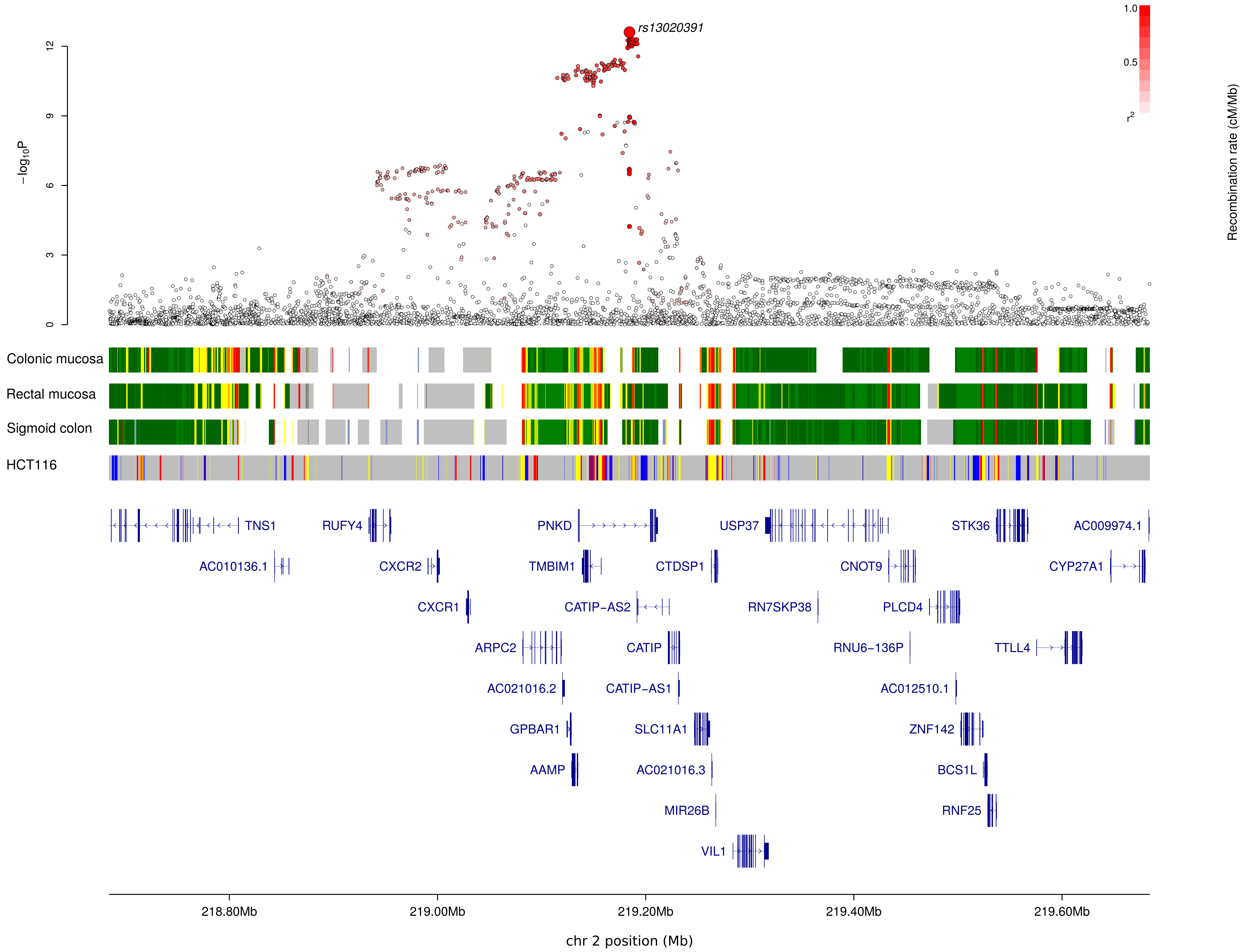
rs13020391
1.0
0.5
r^2
Recombination rate (cM/Mb)
$-\log_{10}P$
12
9
6
3
0
Colonic mucosa
Rectal mucosa
Sigmoid colon
HCT116
TNS1
RUFY4
PNKD
USP37
STK36
AC009974.1
AC010136.1
CXCR2
TMBIM1
CTDSP1
CNOT9
CYP27A1
CXCR1
CATIP−AS2
RN7SKP38
PLCD4
ARPC2
CATIP
RNU6−136P
TTLL4
AC021016.2
CATIP−AS1
AC012510.1
GPBAR1
SLC11A1
ZNF142
AAMP
AC021016.3
BCS1L
MIR26B
RNF25
VIL1
218.80Mb
219.00Mb
219.20Mb
219.40Mb
219.60Mb
chr 2 position (Mb)

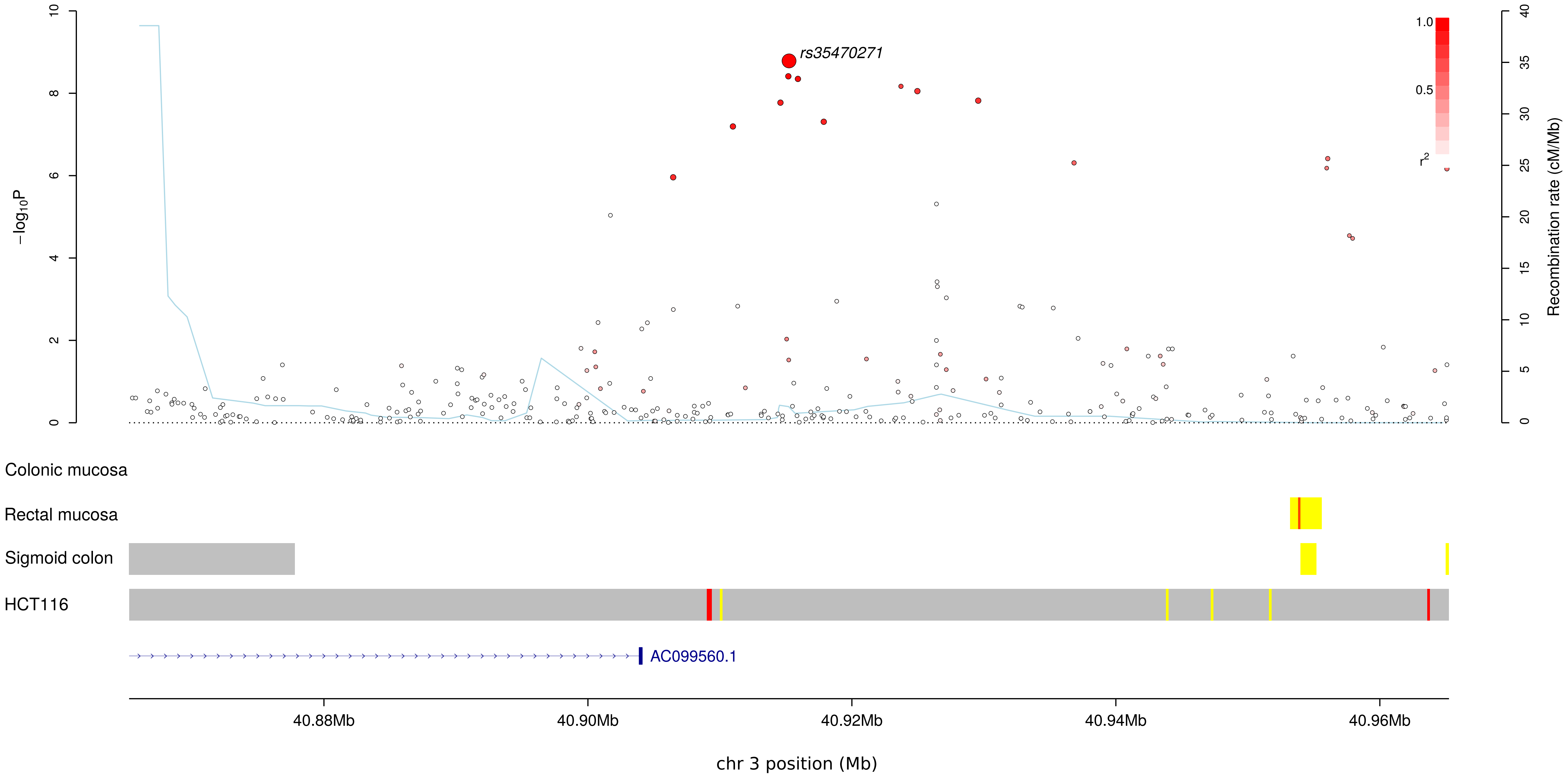

rs35470271
1.0
0.5
r^2
$-\log_{10}P$
Recombination rate (cM/Mb)
Colonic mucosa
Rectal mucosa
Sigmoid colon
HCT116
AC099560.1
40.88Mb
40.90Mb
40.92Mb
40.94Mb
40.96Mb
chr 3 position (Mb)

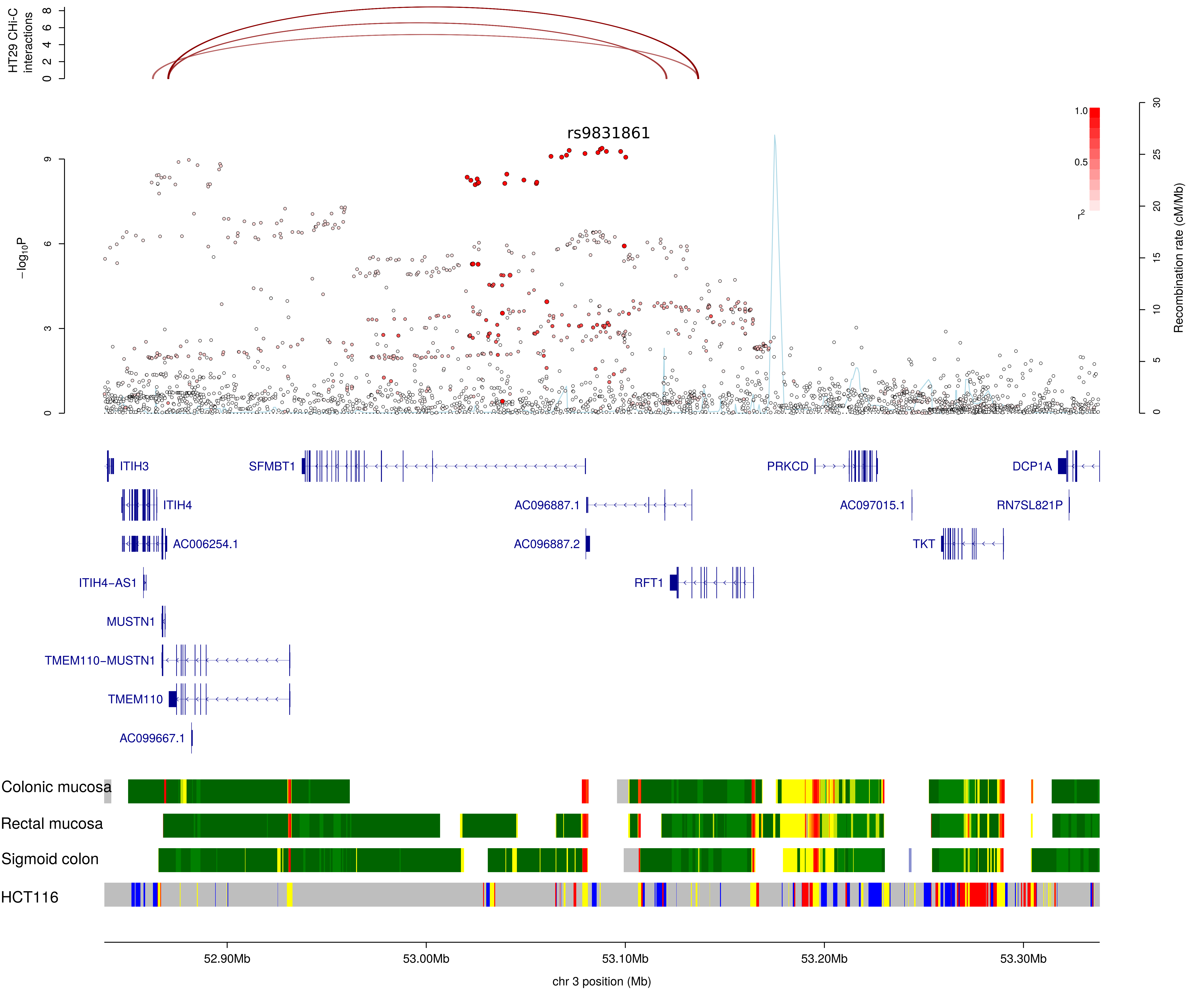

HT29 CHi-C interactions
rs9831861
1.0
0.5
r^2
$-\log_{10}P$
Recombination rate (cM/Mb)
ITIH3
SFMBT1
PRKCD
DCP1A
ITIH4
AC096887.1
AC097015.1
RN7SL821P
AC006254.1
AC096887.2
TKT
ITIH4-AS1
RFT1
MUSTN1
TMEM110-MUSTN1
TMEM110
AC099667.1
Colonic mucosa
Rectal mucosa
Sigmoid colon
HCT116
52.90Mb
53.00Mb
53.10Mb
53.20Mb
53.30Mb
chr 3 position (Mb)

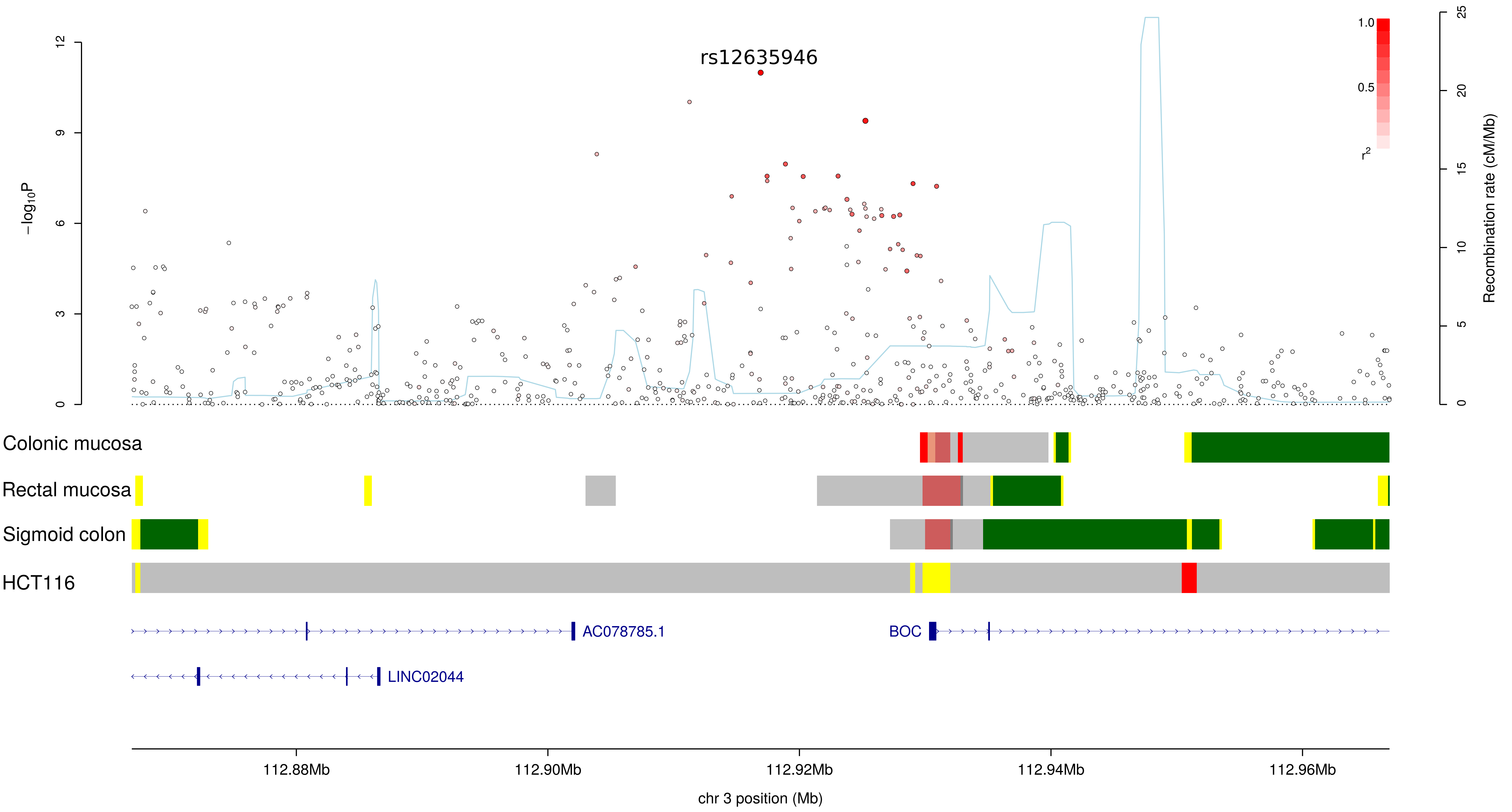
rs12635946
−log10P
0
3
6
9
12
Recombination rate (cM/Mb)
0
5
10
15
20
25
1.0
0.5
r2
Colonic mucosa
Rectal mucosa
Sigmoid colon
HCT116
AC078785.1
BOC
LINC02044
112.88Mb
112.90Mb
112.92Mb
112.94Mb
112.96Mb
chr 3 position (Mb)

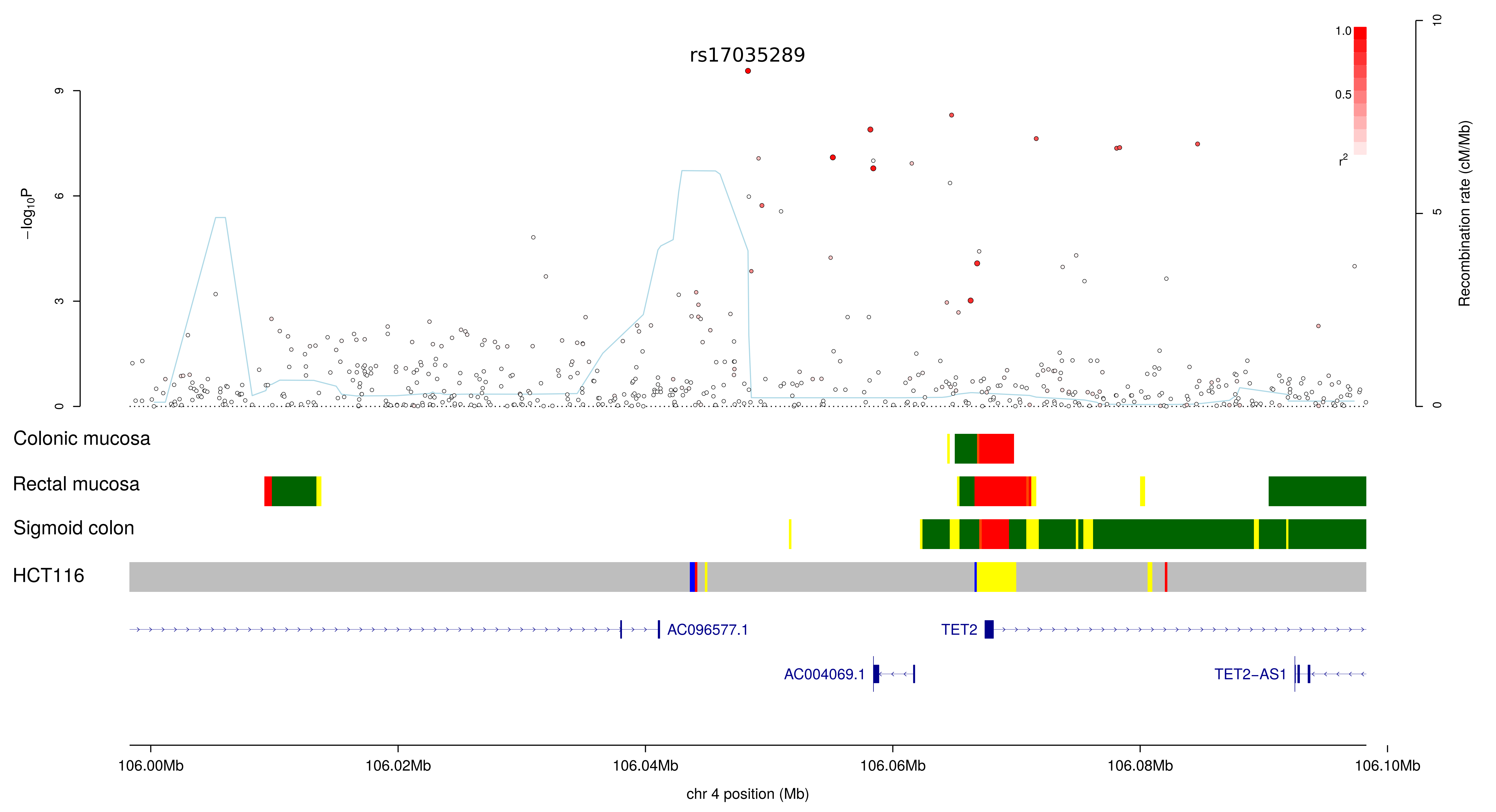

rs17035289
1.0
0.5
r2
−log10P
0
3
6
9
Recombination rate (cM/Mb)
0
5
10
Colonic mucosa
Rectal mucosa
Sigmoid colon
HCT116
AC096577.1
TET2
AC004069.1
TET2−AS1
106.00Mb
106.02Mb
106.04Mb
106.06Mb
106.08Mb
106.10Mb
chr 4 position (Mb)

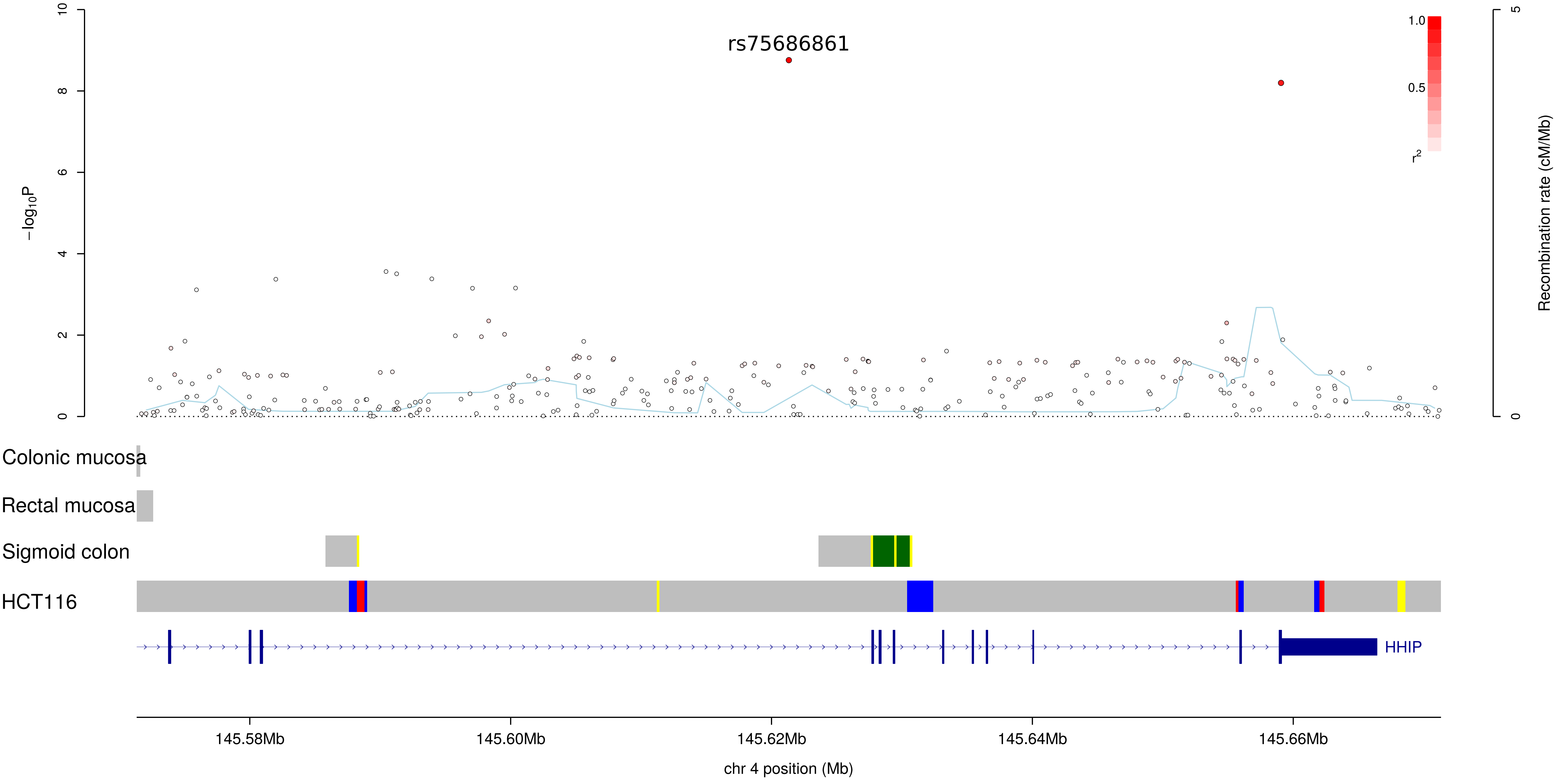

rs75686861
−log10P
10
8
6
4
2
0
1.0
0.5
r2
Recombination rate (cM/Mb)
5
0
Colonic mucosa
Rectal mucosa
Sigmoid colon
HCT116
HHIP
145.58Mb
145.60Mb
145.62Mb
145.64Mb
145.66Mb
chr 4 position (Mb)

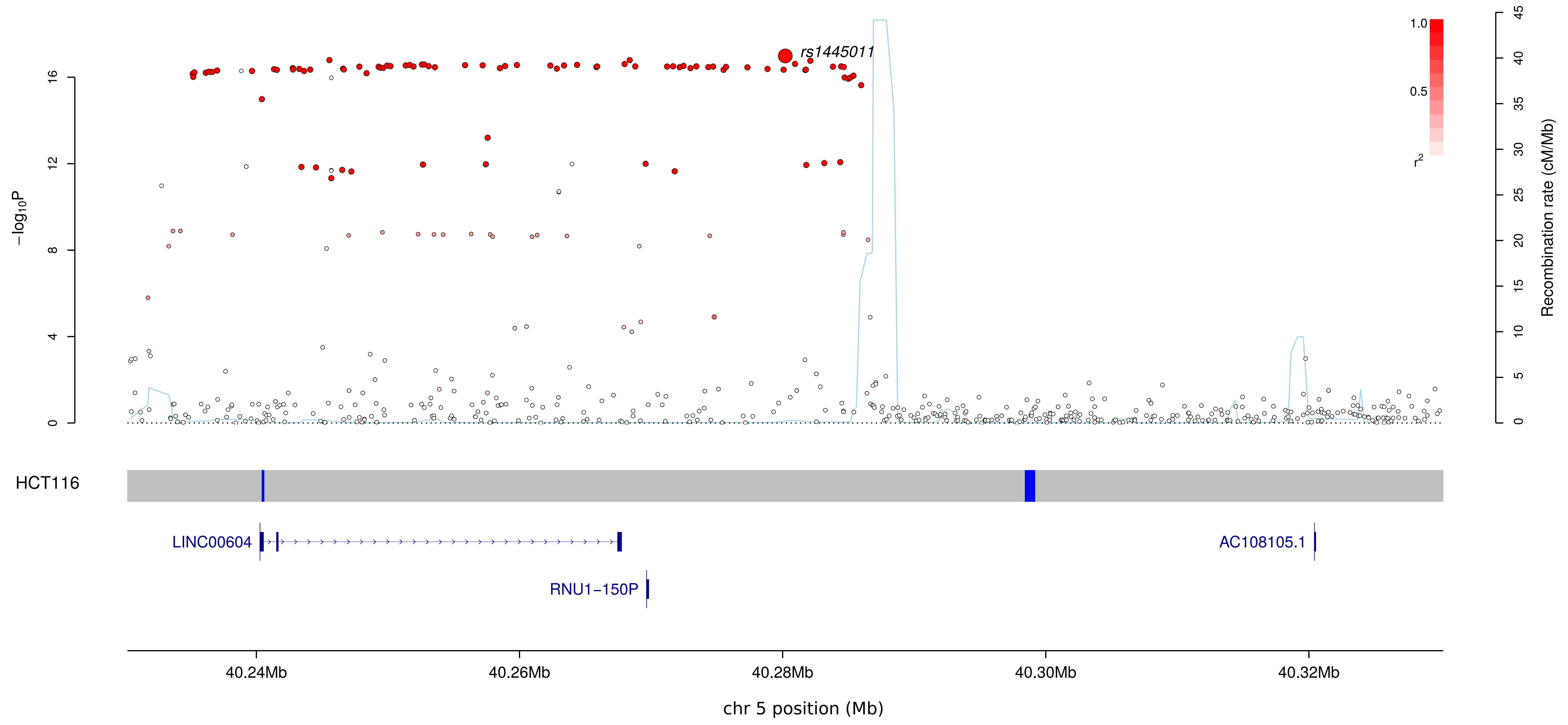

rs1445011
1.0
0.5
r^2
$-\log_{10}P$
0
4
8
12
16
Recombination rate (cM/Mb)
0
5
10
15
20
25
30
35
40
45
HCT116
LINC00604
RNU1−150P
AC108105.1
40.24Mb
40.26Mb
40.28Mb
40.30Mb
40.32Mb
chr 5 position (Mb)

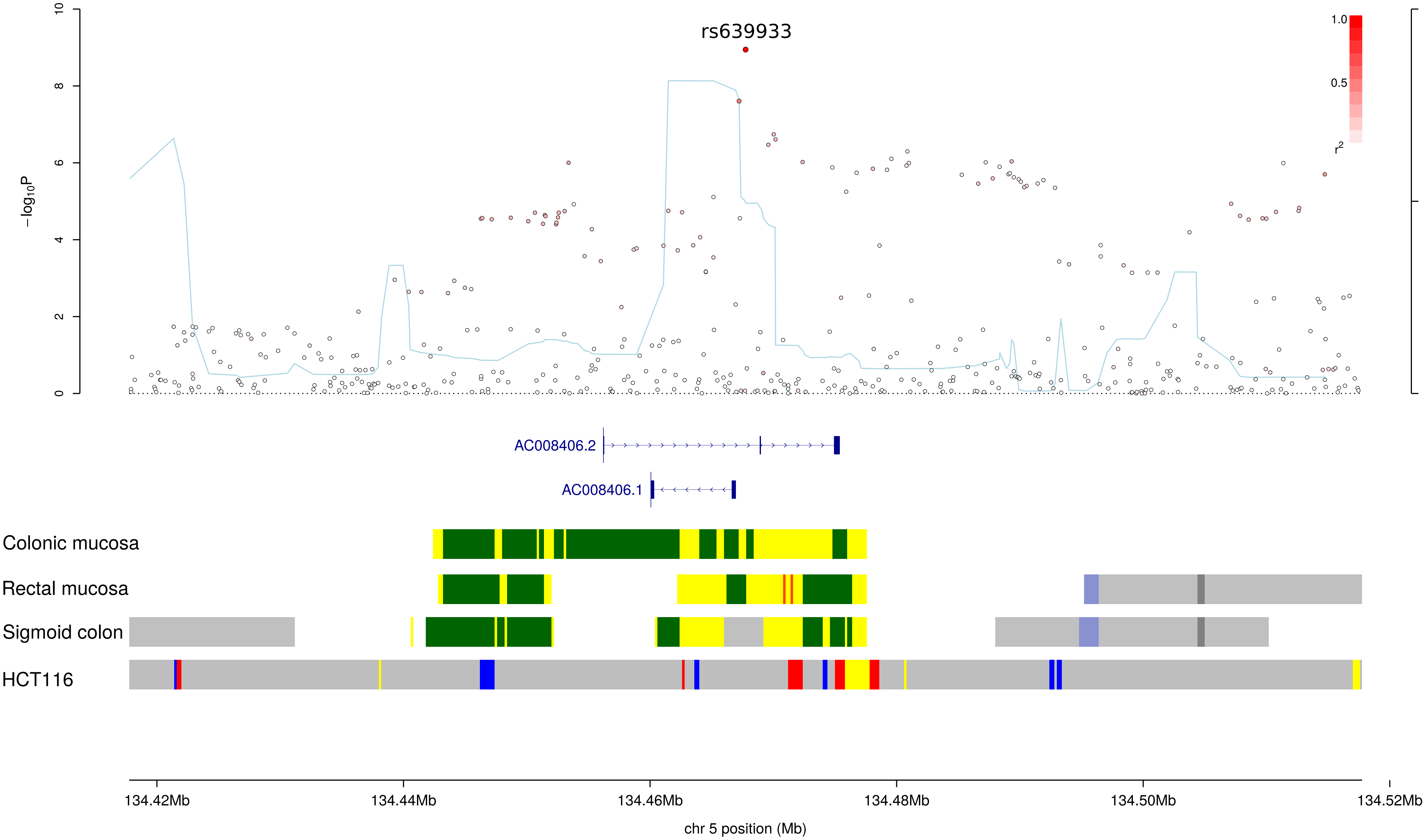

rs639933
−log10P
Recombination rate (cM/Mb)
1.0
0.5
r2
AC008406.2
AC008406.1
Colonic mucosa
Rectal mucosa
Sigmoid colon
HCT116
134.42Mb
134.44Mb
134.46Mb
134.48Mb
134.50Mb
134.52Mb
chr 5 position (Mb)

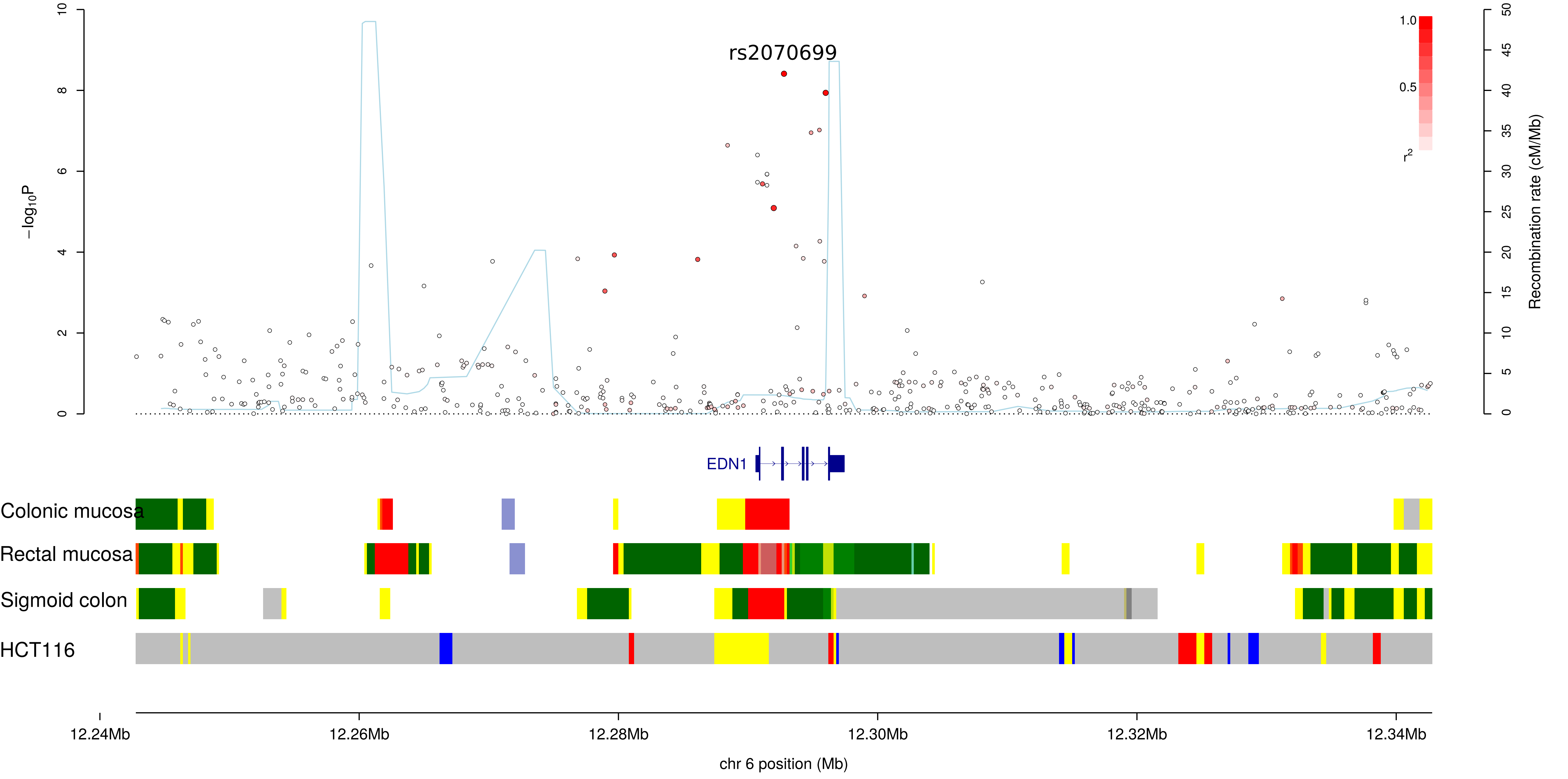
rs2070699
−log10P
Recombination rate (cM/Mb)
1.0
0.5
r2
EDN1
Colonic mucosa
Rectal mucosa
Sigmoid colon
HCT116
12.24Mb
12.26Mb
12.28Mb
12.30Mb
12.32Mb
12.34Mb
chr 6 position (Mb)

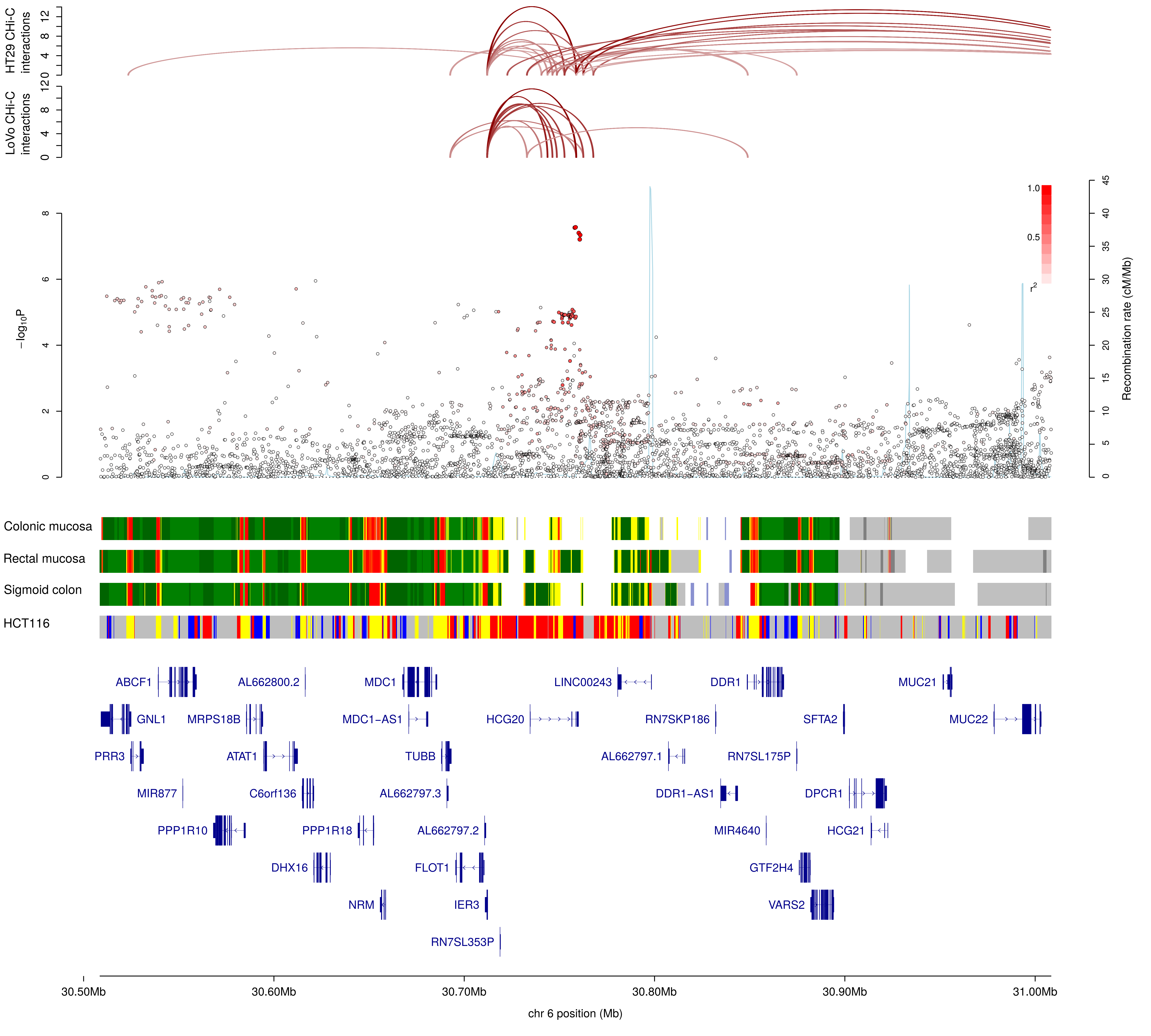

HT29 CHi-C interactions
LoVo CHi-C interactions
−log10P
Recombination rate (cM/Mb)
r2
Colonic mucosa
Rectal mucosa
Sigmoid colon
HCT116
ABCF1
AL662800.2
MDC1
LINC00243
DDR1
MUC21
GNL1
MRPS18B
MDC1-AS1
HCG20
RN7SKP186
SFTA2
MUC22
PRR3
ATAT1
TUBB
AL662797.1
RN7SL175P
MIR877
C6orf136
AL662797.3
DDR1-AS1
DPCR1
PPP1R10
PPP1R18
AL662797.2
MIR4640
HCG21
DHX16
FLOT1
GTF2H4
NRM
IER3
VARS2
RN7SL353P
30.50Mb
30.60Mb
30.70Mb
30.80Mb
30.90Mb
31.00Mb
chr 6 position (Mb)

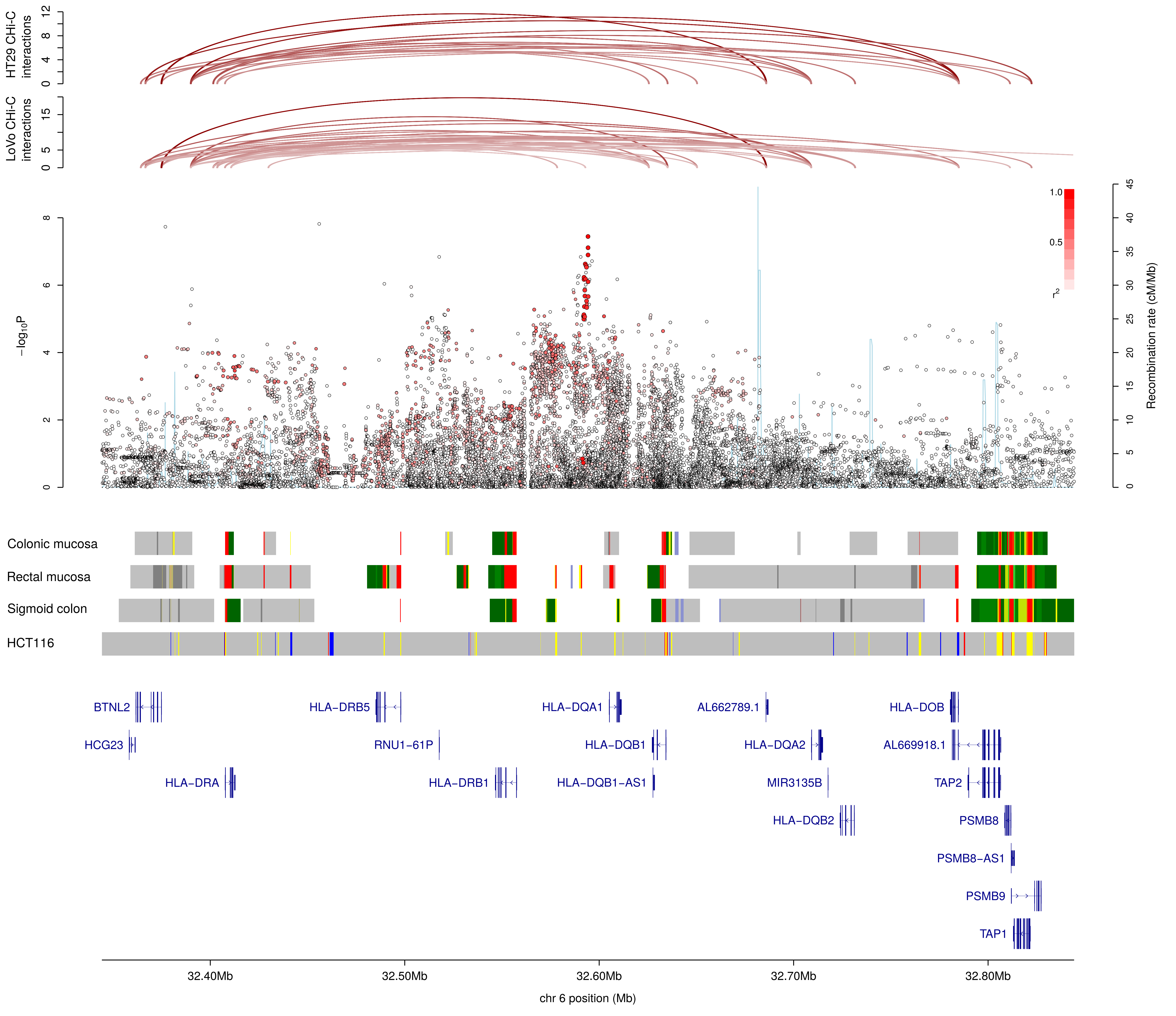

HT29 CHi-C interactions
LoVo CHi-C interactions
−log10P
Recombination rate (cM/Mb)
r²
Colonic mucosa
Rectal mucosa
Sigmoid colon
HCT116
BTNL2
HCG23
HLA−DRA
HLA−DRB5
RNU1−61P
HLA−DRB1
HLA−DQA1
HLA−DQB1
HLA−DQB1−AS1
AL662789.1
HLA−DQA2
MIR3135B
HLA−DQB2
HLA−DOB
AL669918.1
TAP2
PSMB8
PSMB8−AS1
PSMB9
TAP1
32.40Mb
32.50Mb
32.60Mb
32.70Mb
32.80Mb
chr 6 position (Mb)

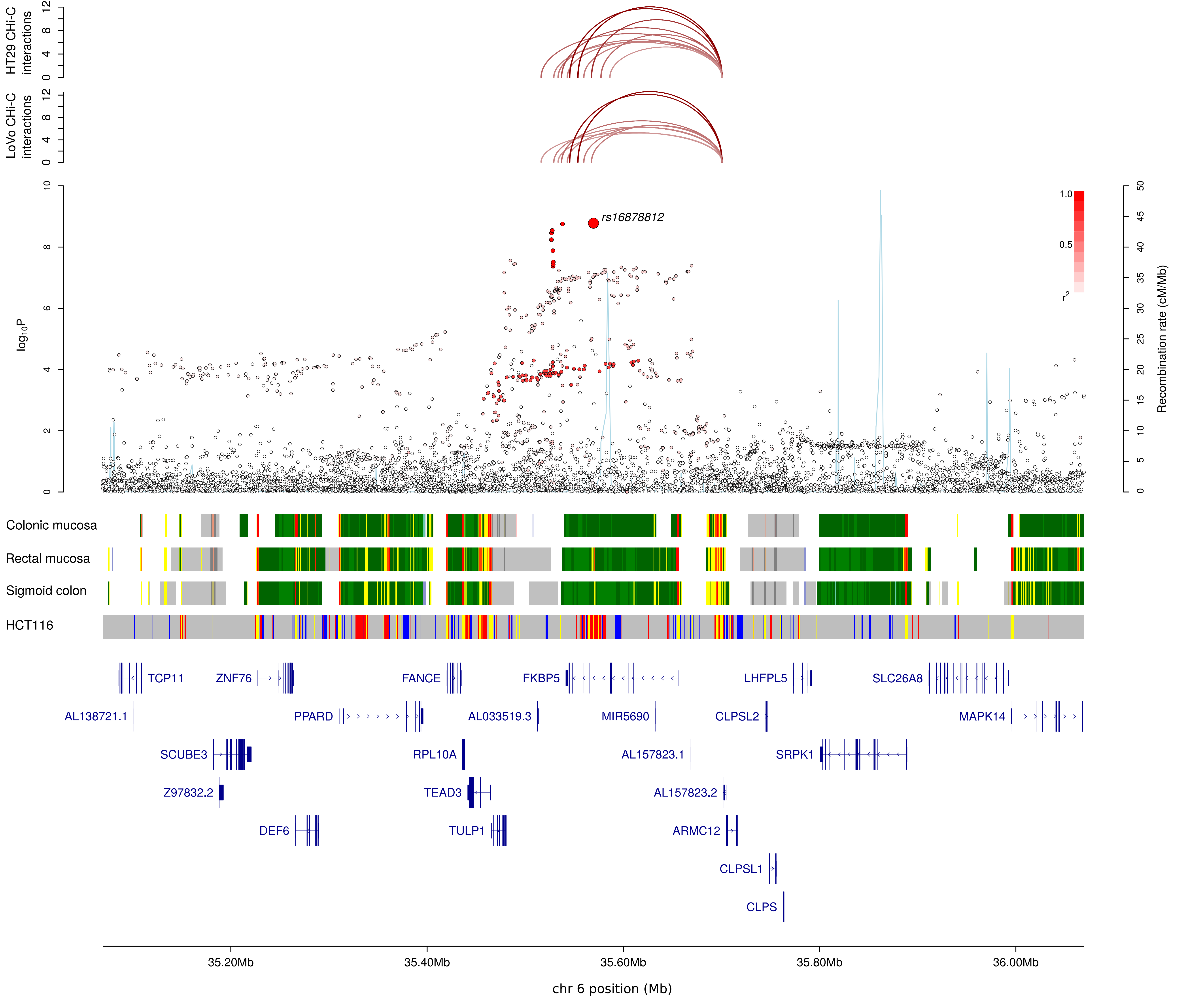

HT29 CHi-C interactions
LoVo CHi-C interactions
rs16878812
−log10P
Recombination rate (cM/Mb)
1.0
0.5
r2
Colonic mucosa
Rectal mucosa
Sigmoid colon
HCT116
TCP11
ZNF76
FANCE
FKBP5
LHFPL5
SLC26A8
AL138721.1
PPARD
AL033519.3
MIR5690
CLPSL2
MAPK14
SCUBE3
RPL10A
AL157823.1
SRPK1
Z97832.2
TEAD3
AL157823.2
DEF6
TULP1
ARMC12
CLPSL1
CLPS
35.20Mb
35.40Mb
35.60Mb
35.80Mb
36.00Mb
chr 6 position (Mb)

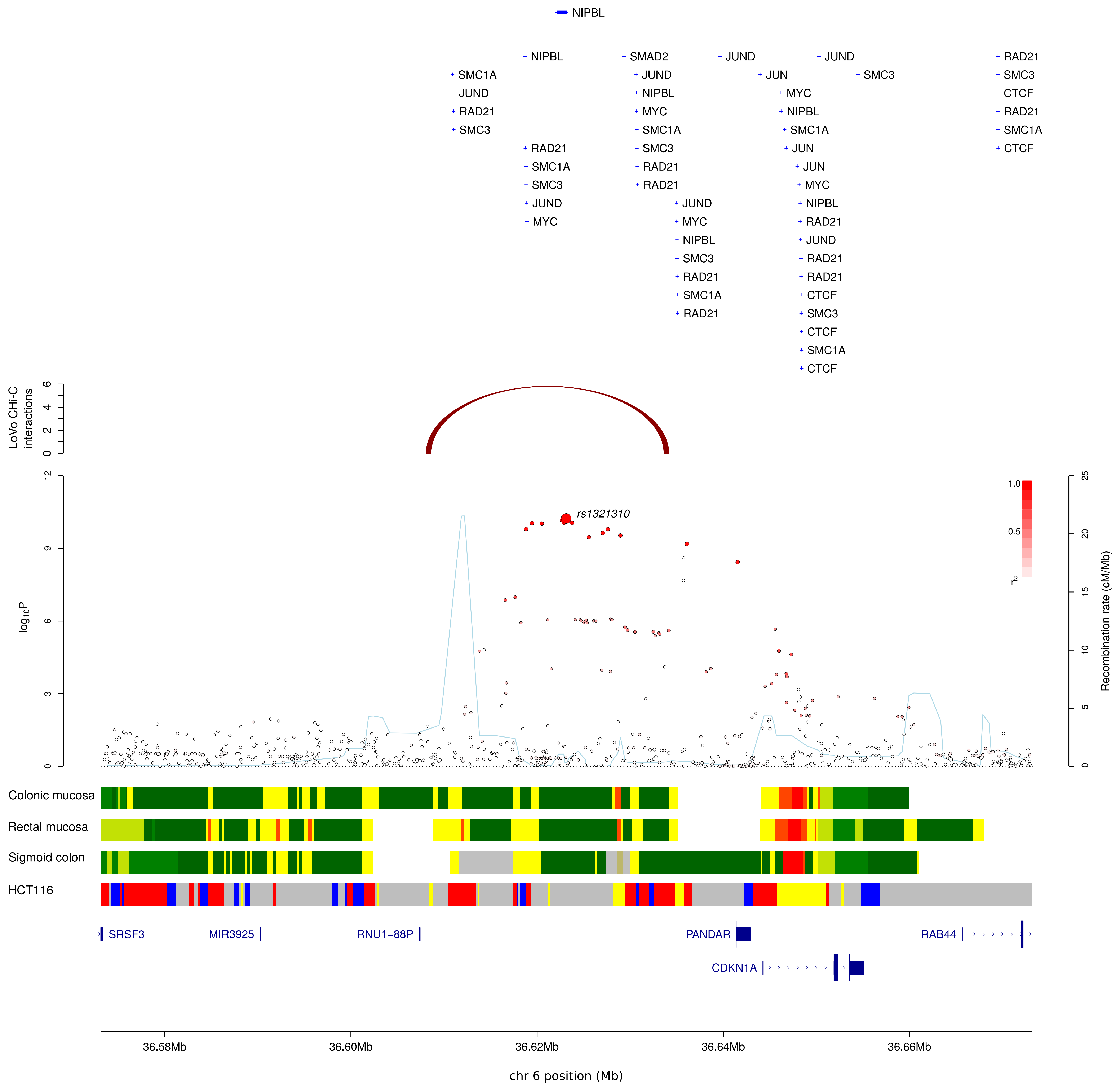

NIPBL
NIPBL
SMC1A
JUND
RAD21
SMC3
RAD21
SMC1A
SMC3
JUND
MYC
SMAD2
JUND
NIPBL
MYC
SMC1A
SMC3
RAD21
RAD21
JUND
MYC
NIPBL
SMC3
RAD21
SMC1A
RAD21
JUND
JUN
MYC
NIPBL
SMC1A
JUN
JUN
MYC
NIPBL
RAD21
JUND
RAD21
RAD21
CTCF
SMC3
CTCF
SMC1A
CTCF
JUND
SMC3
RAD21
SMC3
CTCF
RAD21
SMC1A
CTCF
LoVo CHi-C interactions
0
2
4
6
rs1321310
−log10P
0
3
6
9
12
1.0
0.5
r2
Recombination rate (cM/Mb)
0
5
10
15
20
25
Colonic mucosa
Rectal mucosa
Sigmoid colon
HCT116
SRSF3
MIR3925
RNU1−88P
PANDAR
CDKN1A
RAB44
36.58Mb
36.60Mb
36.62Mb
36.64Mb
36.66Mb
chr 6 position (Mb)

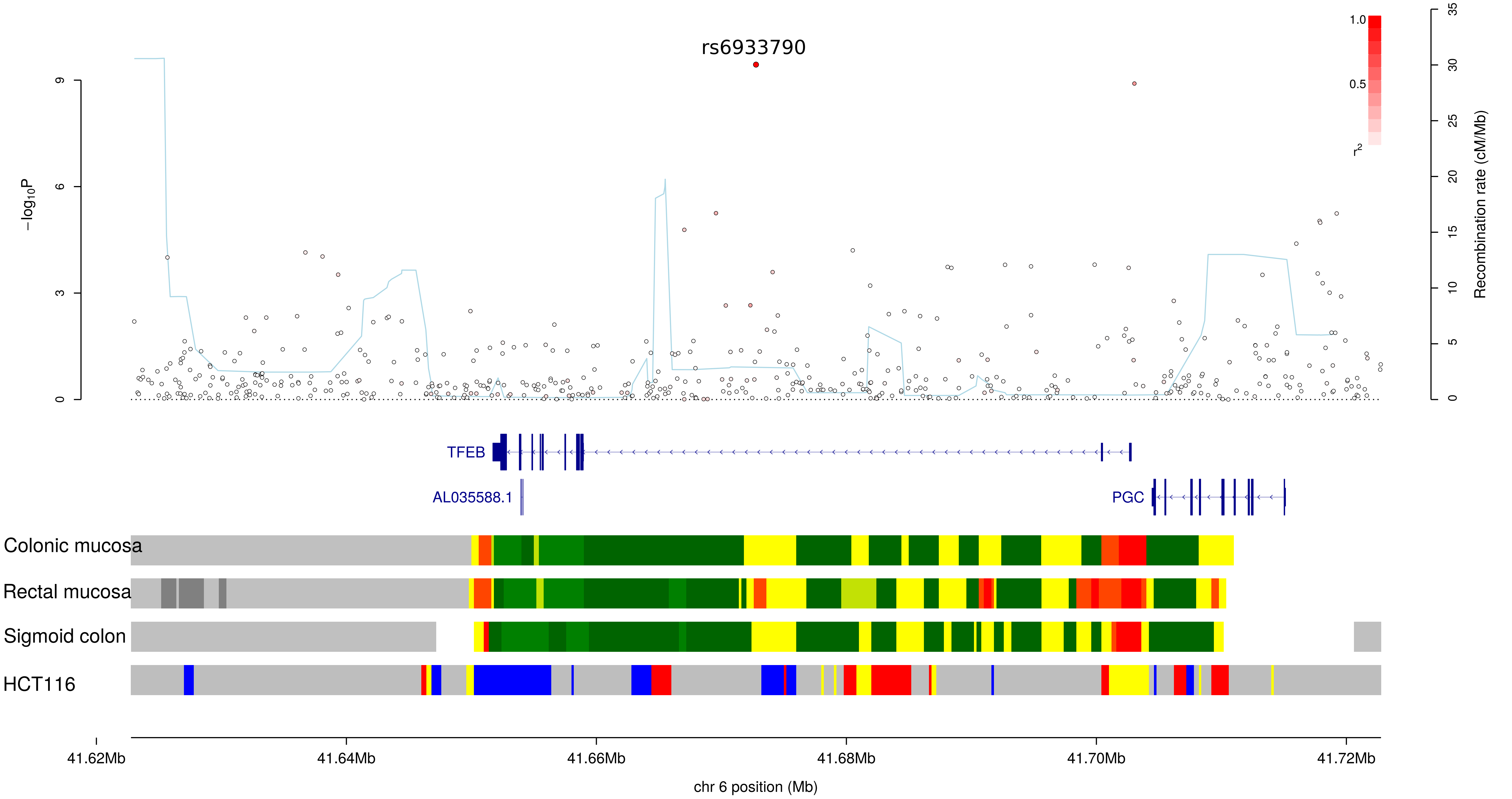

rs6933790
−log10P
0
3
6
9
1.0
0.5
r2
Recombination rate (cM/Mb)
0
5
10
15
20
25
30
35
TFEB
AL035588.1
PGC
Colonic mucosa
Rectal mucosa
Sigmoid colon
HCT116
41.62Mb
41.64Mb
41.66Mb
41.68Mb
41.70Mb
41.72Mb
chr 6 position (Mb)

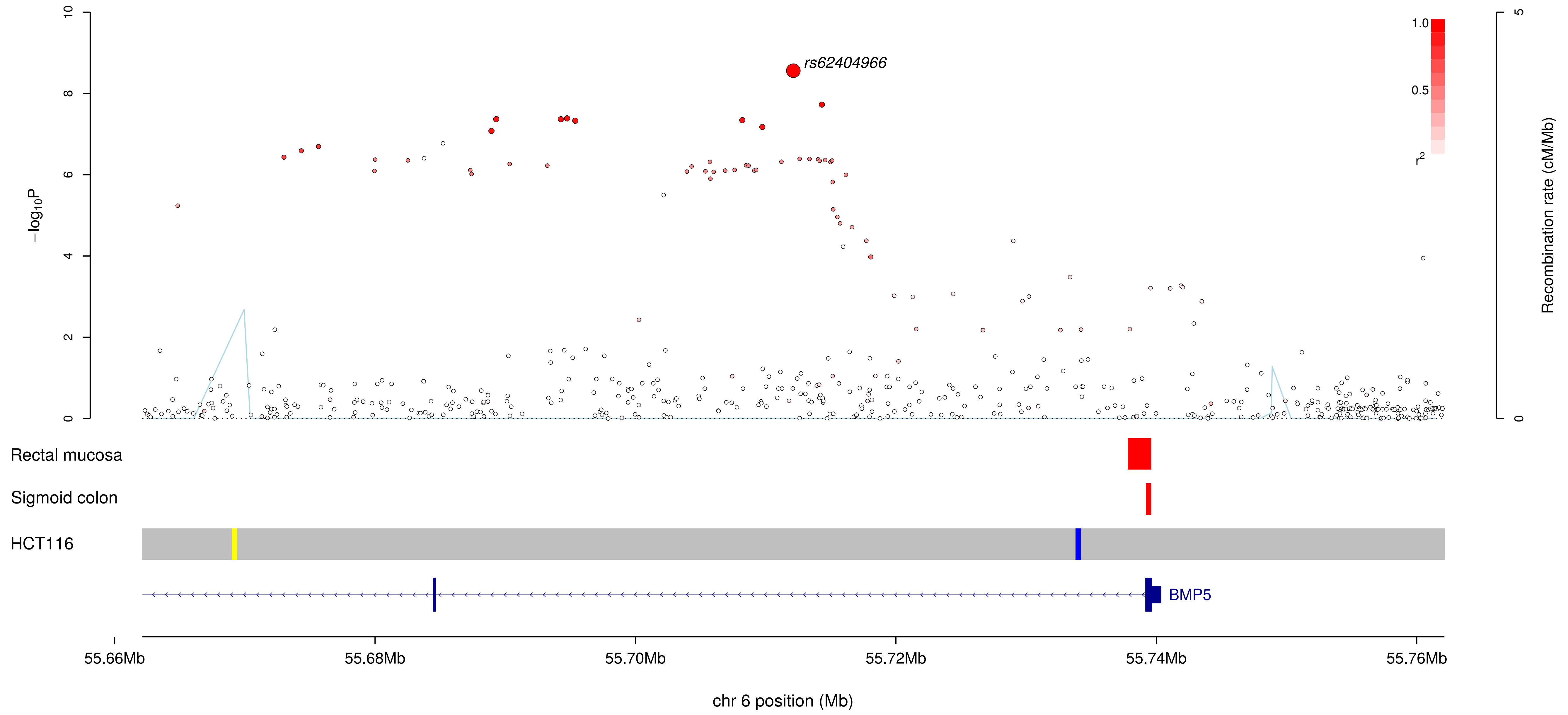

10
8
6
4
2
0
−log10P
rs62404966
1.0
0.5
r2
5
0
Recombination rate (cM/Mb)
Rectal mucosa
Sigmoid colon
HCT116
BMP5
55.66Mb
55.68Mb
55.70Mb
55.72Mb
55.74Mb
55.76Mb
chr 6 position (Mb)

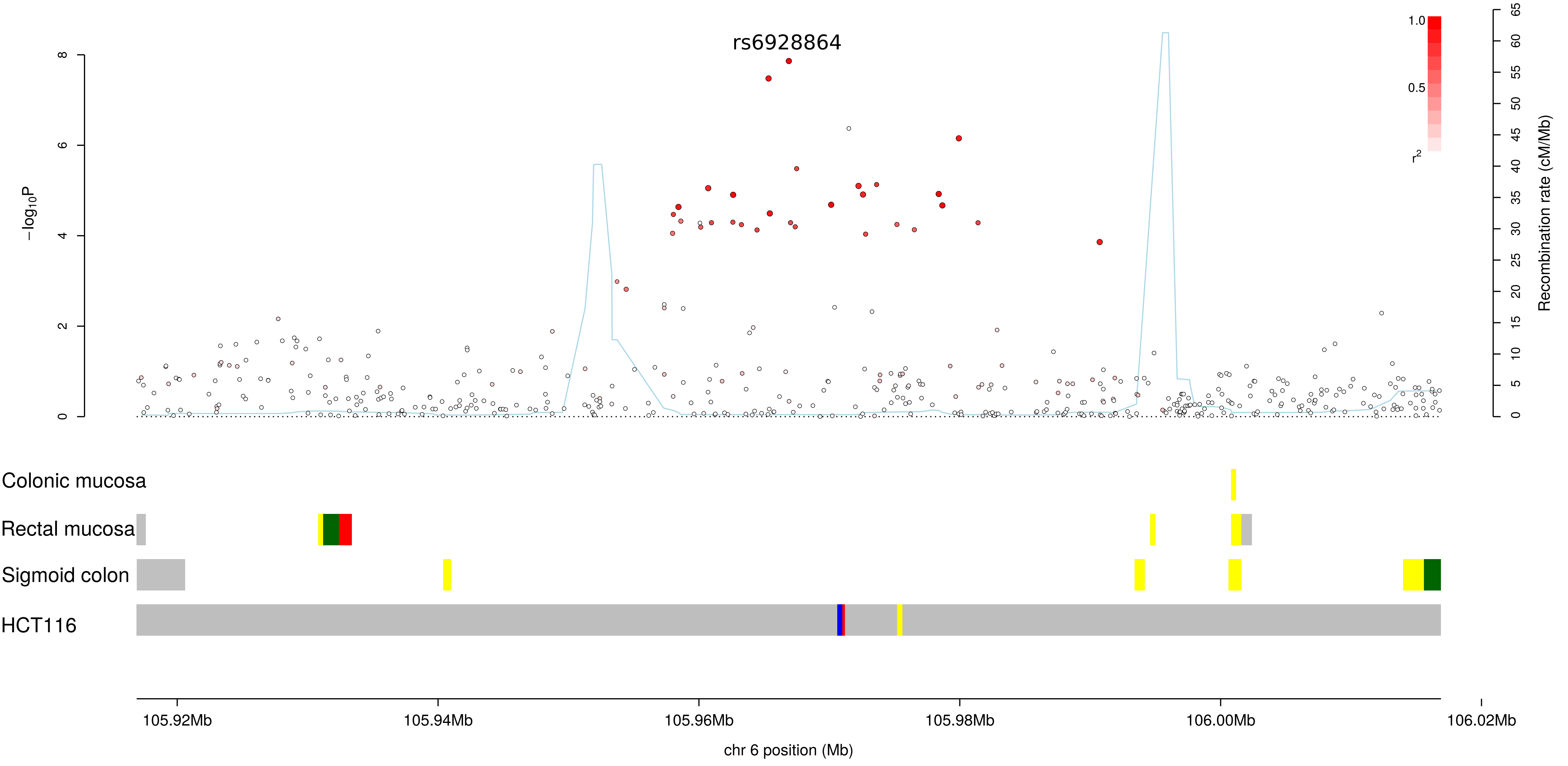

rs6928864
−log10P
Recombination rate (cM/Mb)
1.0
0.5
r2
Colonic mucosa
Rectal mucosa
Sigmoid colon
HCT116
105.92Mb
105.94Mb
105.96Mb
105.98Mb
106.00Mb
106.02Mb
chr 6 position (Mb)

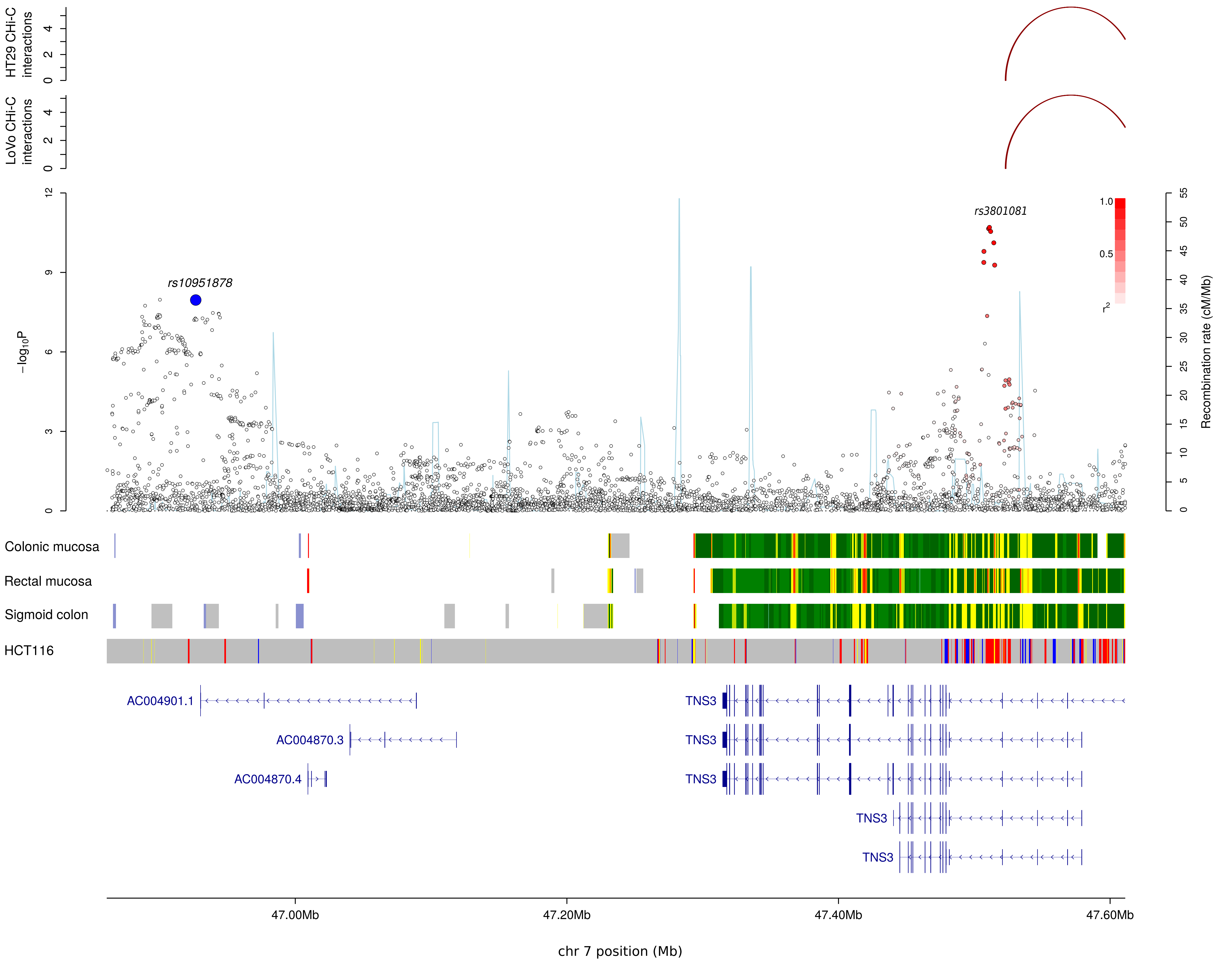

HT29 CHi-C interactions
LoVo CHi-C interactions
−log10P
Recombination rate (cM/Mb)
rs10951878
rs3801081
r2
Colonic mucosa
Rectal mucosa
Sigmoid colon
HCT116
AC004901.1
AC004870.3
AC004870.4
TNS3
47.00Mb
47.20Mb
47.40Mb
47.60Mb
chr 7 position (Mb)

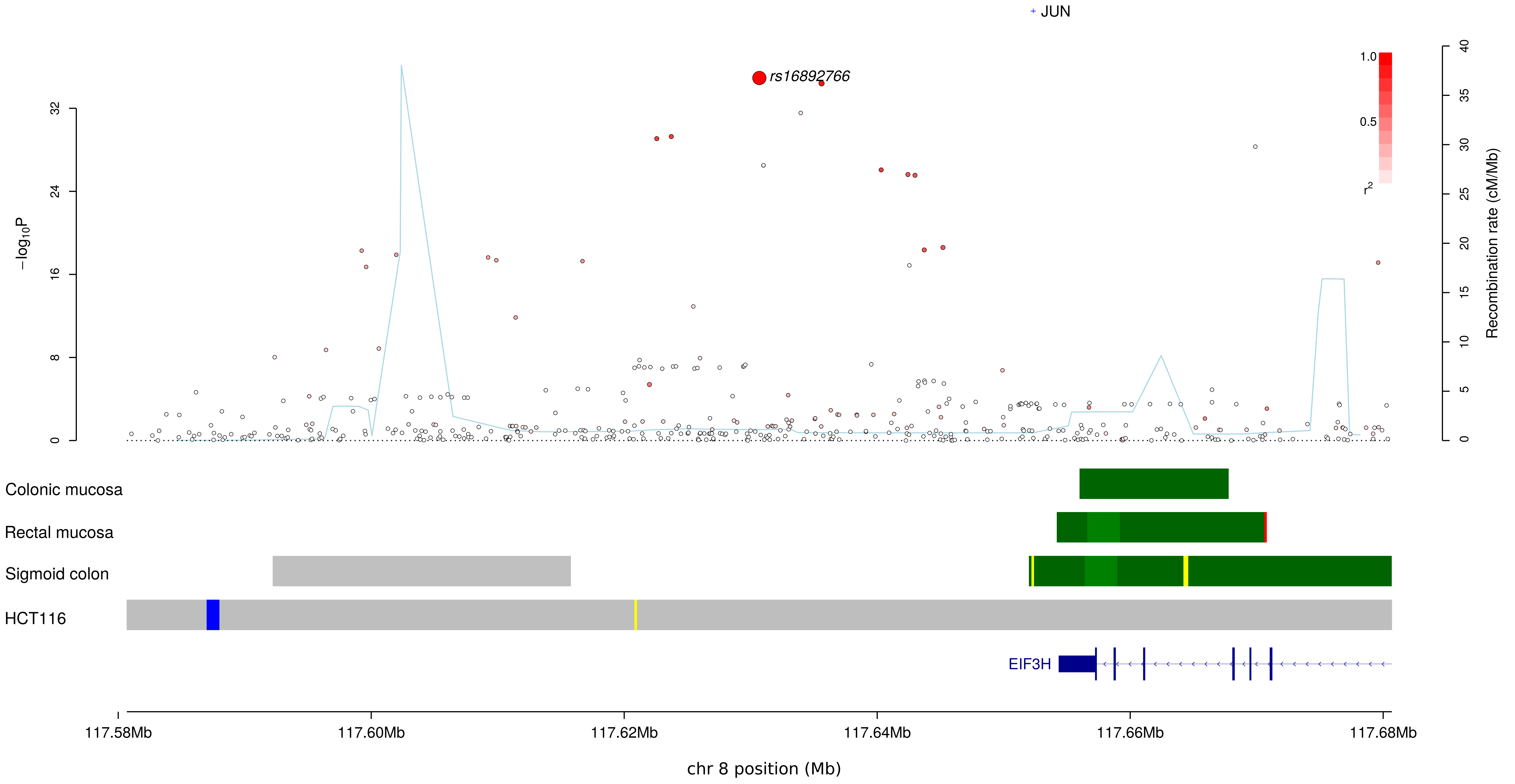
JUN
rs16892766
1.0
0.5
r^2
$-\log_{10}P$
0
8
16
24
32
Recombination rate (cM/Mb)
0
5
10
15
20
25
30
35
40
Colonic mucosa
Rectal mucosa
Sigmoid colon
HCT116
EIF3H
117.58Mb
117.60Mb
117.62Mb
117.64Mb
117.66Mb
117.68Mb
chr 8 position (Mb)

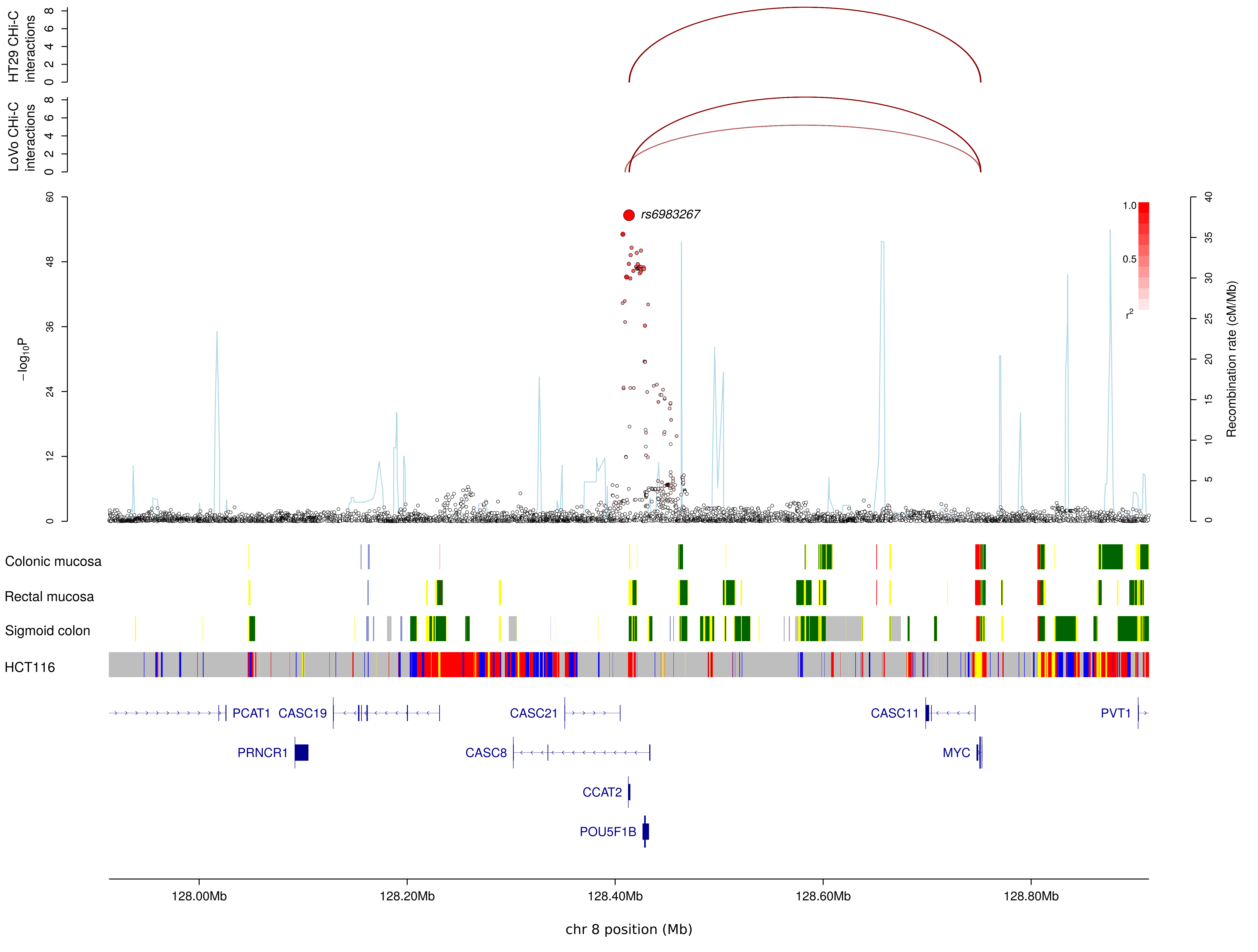
HT29 CHi-C interactions
LoVo CHi-C interactions
rs6983267
−log10P
Recombination rate (cM/Mb)
r2
Colonic mucosa
Rectal mucosa
Sigmoid colon
HCT116
PCAT1
CASC19
CASC21
CASC11
PVT1
PRNCR1
CASC8
MYC
CCAT2
POU5F1B
128.00Mb
128.20Mb
128.40Mb
128.60Mb
128.80Mb
chr 8 position (Mb)

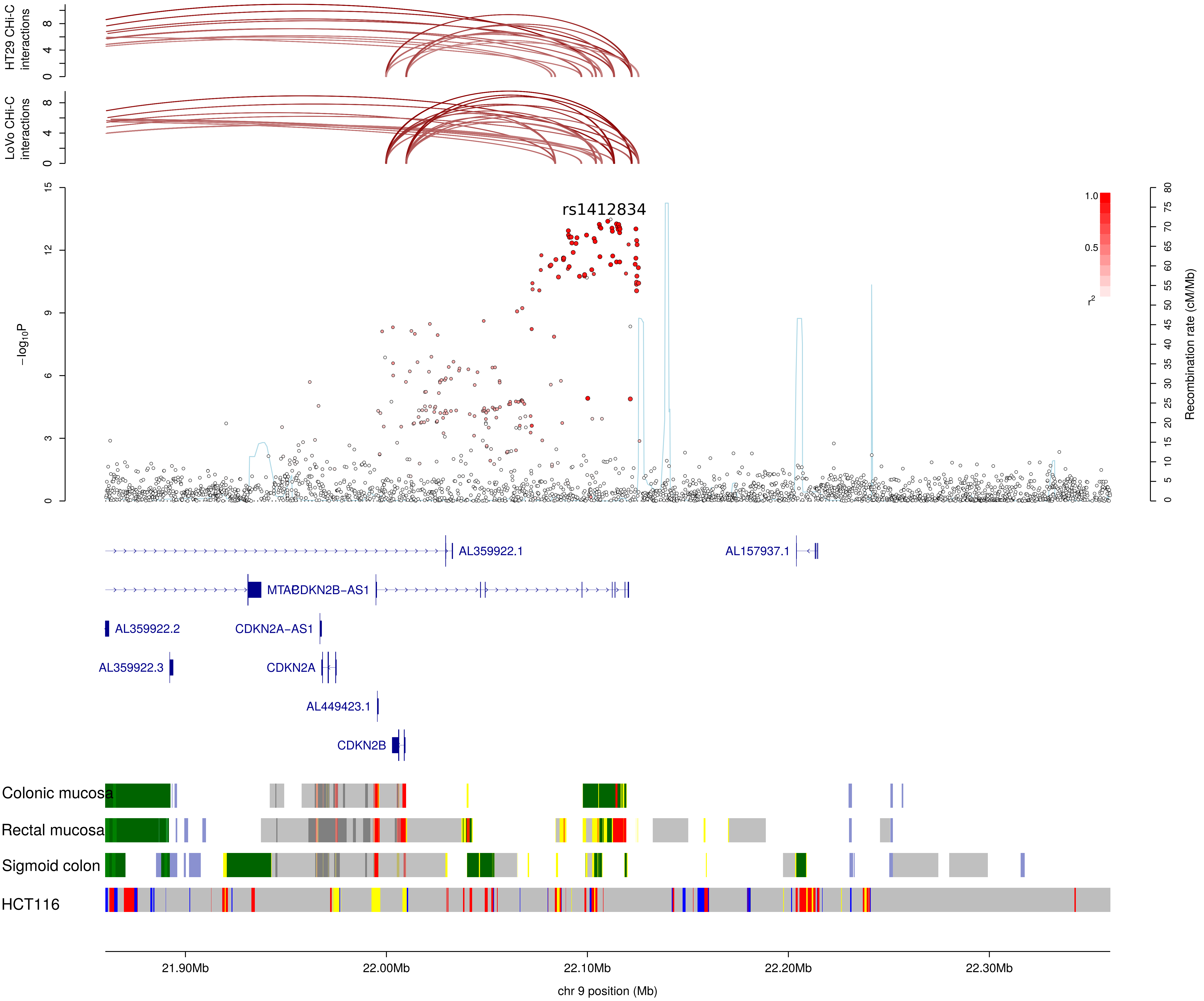

HT29 CHi-C interactions
LoVo CHi-C interactions
rs1412834
−log10P
Recombination rate (cM/Mb)
r2
AL359922.1
AL157937.1
MTAP
CDKN2B-AS1
AL359922.2
CDKN2A-AS1
AL359922.3
CDKN2A
AL449423.1
CDKN2B
Colonic mucosa
Rectal mucosa
Sigmoid colon
HCT116
21.90Mb
22.00Mb
22.10Mb
22.20Mb
22.30Mb
chr 9 position (Mb)

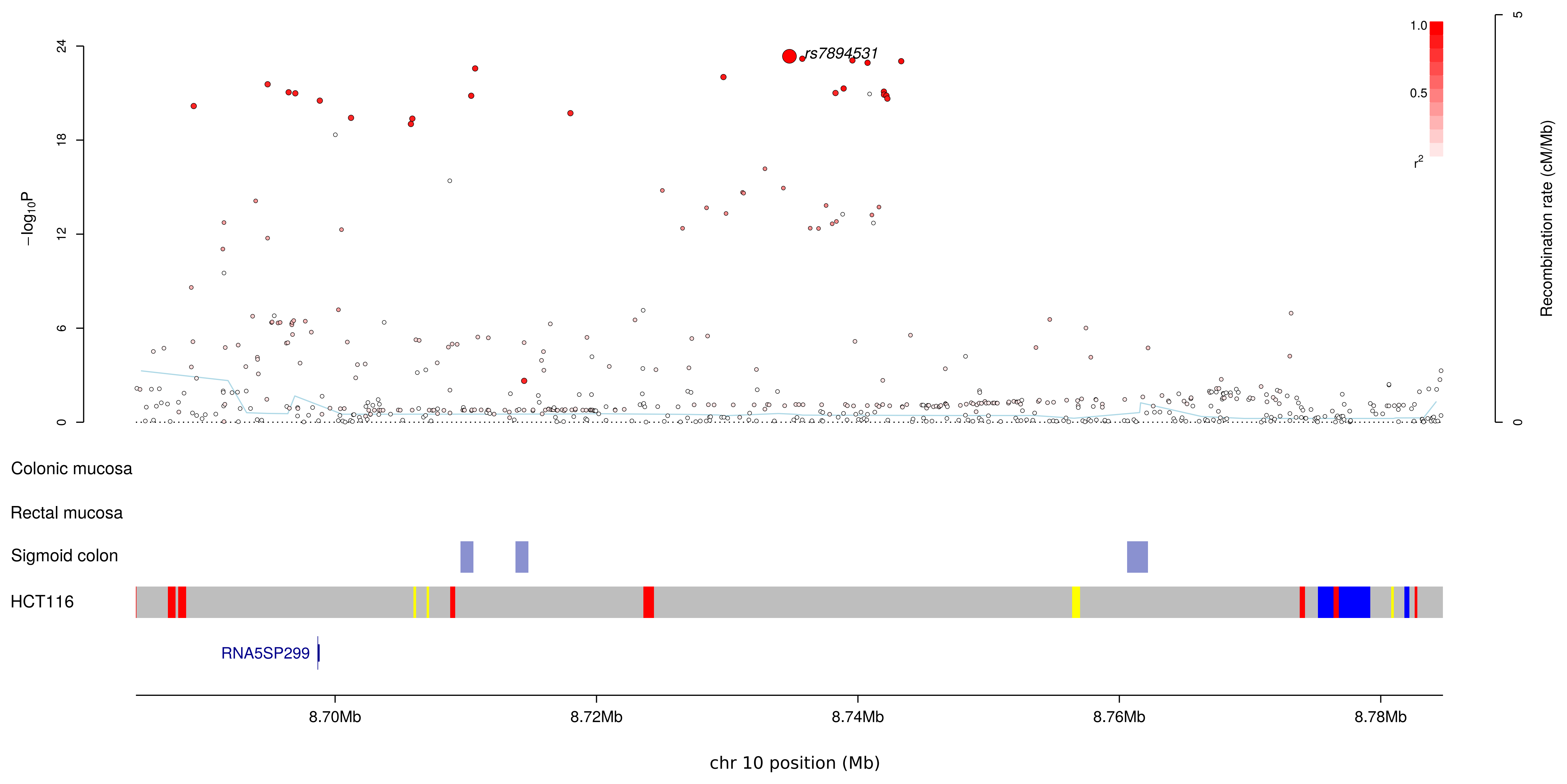
rs7894531
−log10P
24
18
12
6
0
1.0
0.5
r2
Recombination rate (cM/Mb)
5
0
Colonic mucosa
Rectal mucosa
Sigmoid colon
HCT116
RNA5SP299
8.70Mb
8.72Mb
8.74Mb
8.76Mb
8.78Mb
chr 10 position (Mb)

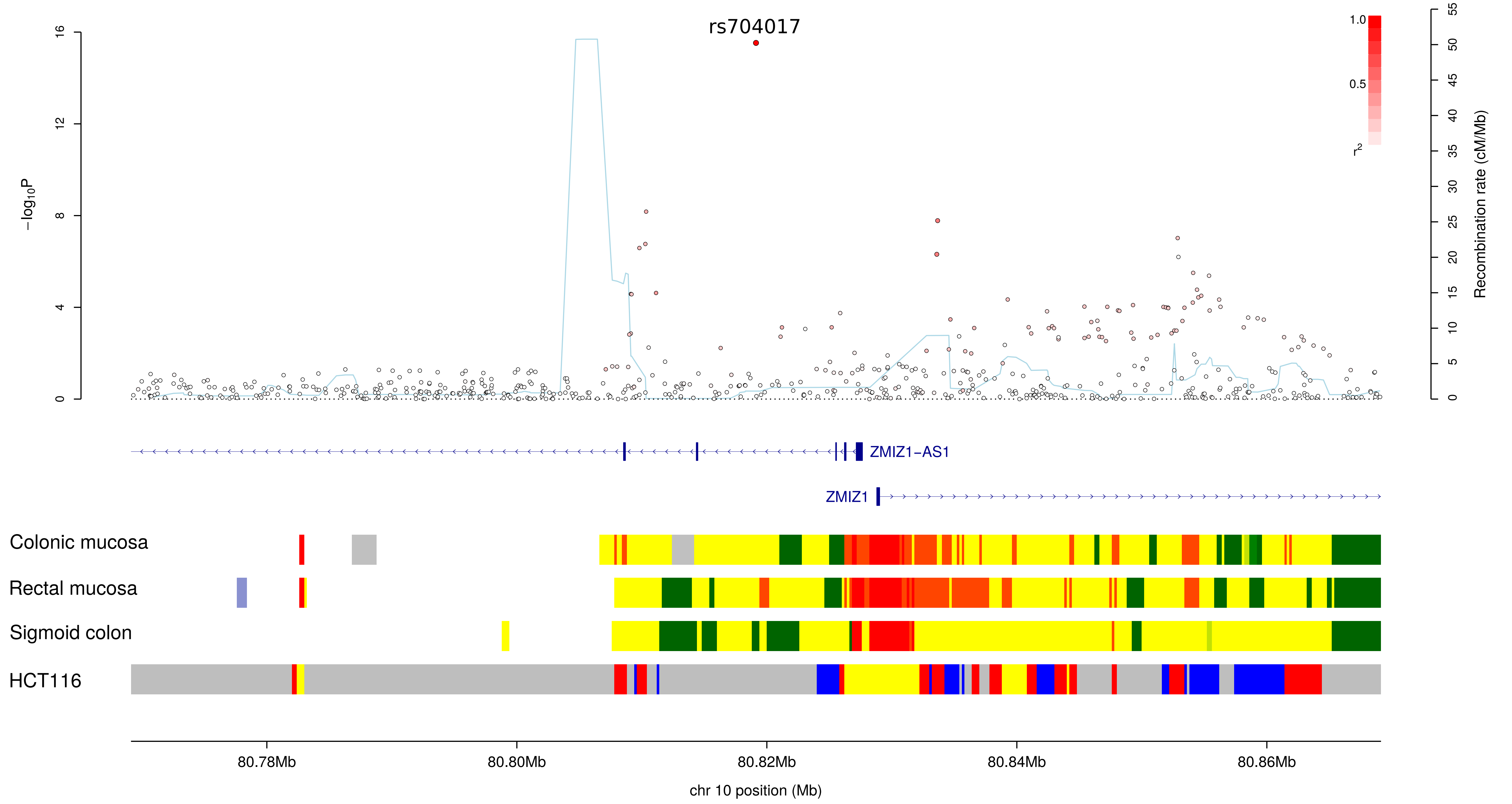
rs704017
$-\log_{10}P$
0
4
8
12
16
Recombination rate (cM/Mb)
1.0
0.5
r^2
ZMIZ1-AS1
ZMIZ1
Colonic mucosa
Rectal mucosa
Sigmoid colon
HCT116
80.78Mb
80.80Mb
80.82Mb
80.84Mb
80.86Mb
chr 10 position (Mb)

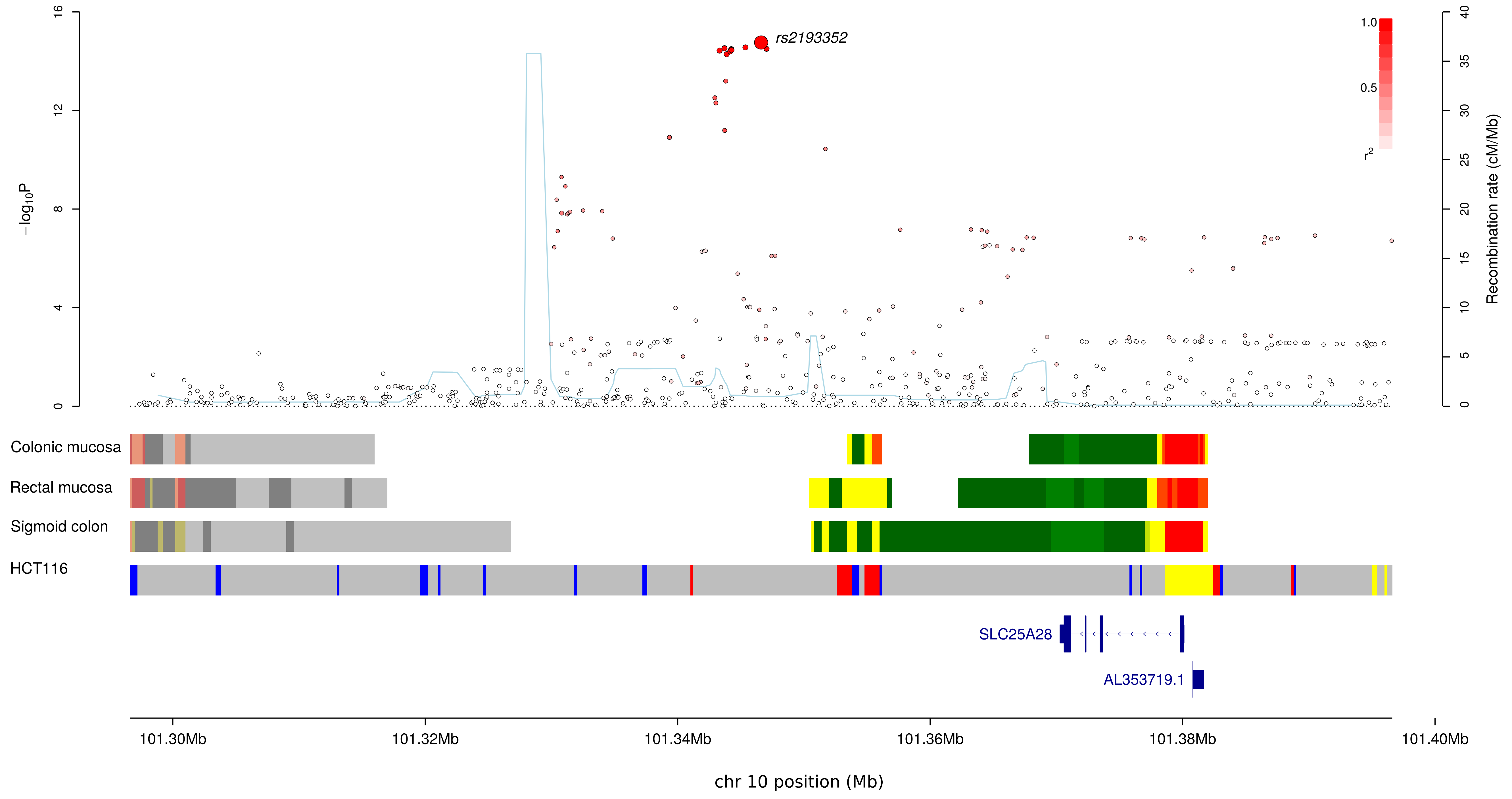
rs2193352
−log10P
16
12
8
4
0
1.0
0.5
r2
Recombination rate (cM/Mb)
40
35
30
25
20
15
10
5
0
Colonic mucosa
Rectal mucosa
Sigmoid colon
HCT116
SLC25A28
AL353719.1
101.30Mb
101.32Mb
101.34Mb
101.36Mb
101.38Mb
101.40Mb
chr 10 position (Mb)

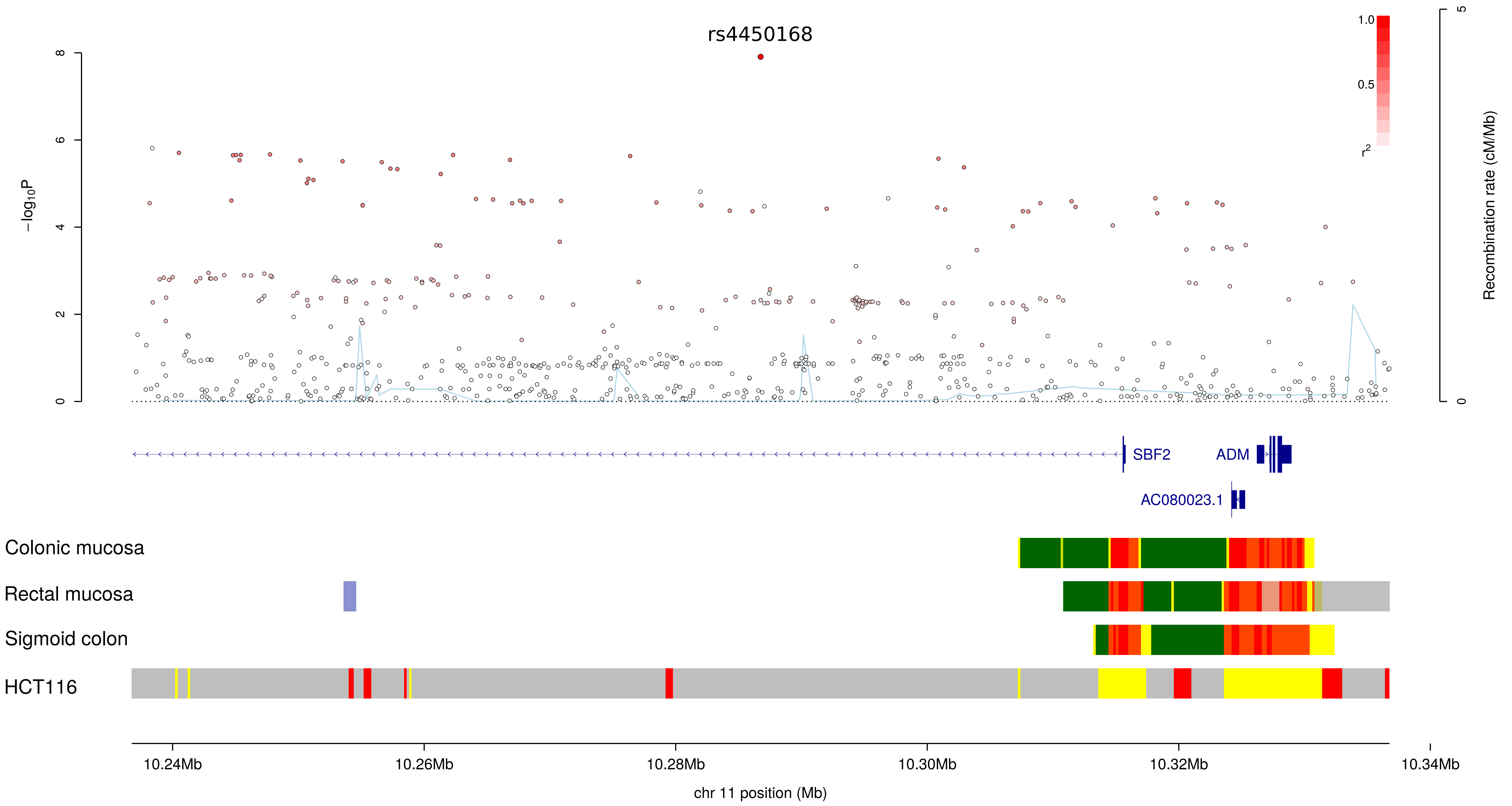

rs4450168
$-\log_{10}P$
8
6
4
2
0
1.0
0.5
r^2
5
0
Recombination rate (cM/Mb)
SBF2
ADM
AC080023.1
Colonic mucosa
Rectal mucosa
Sigmoid colon
HCT116
10.24Mb
10.26Mb
10.28Mb
10.30Mb
10.32Mb
10.34Mb
chr 11 position (Mb)

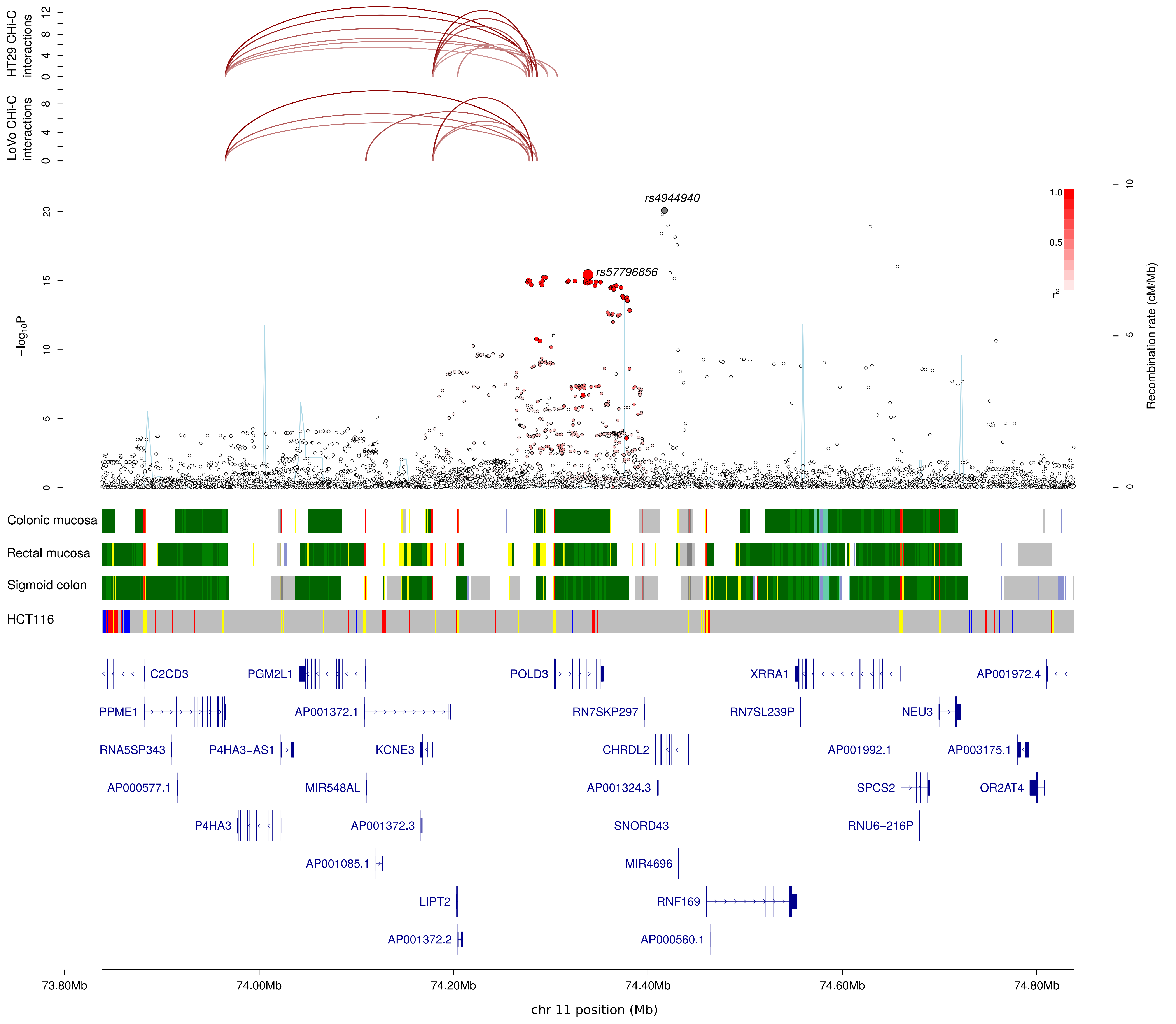

HT29 CHi-C interactions
LoVo CHi-C interactions
rs4944940
rs57796856
−log10P
Recombination rate (cM/Mb)
r2
Colonic mucosa
Rectal mucosa
Sigmoid colon
HCT116
C2CD3
PGM2L1
POLD3
XRRA1
AP001972.4
PPME1
AP001372.1
RN7SKP297
RN7SL239P
NEU3
RNA5SP343
P4HA3-AS1
KCNE3
CHRDL2
AP001992.1
AP003175.1
AP000577.1
MIR548AL
AP001324.3
SPCS2
OR2AT4
P4HA3
AP001372.3
SNORD43
RNU6-216P
AP001085.1
MIR4696
LIPT2
RNF169
AP001372.2
AP000560.1
73.80Mb
74.00Mb
74.20Mb
74.40Mb
74.60Mb
74.80Mb
chr 11 position (Mb)

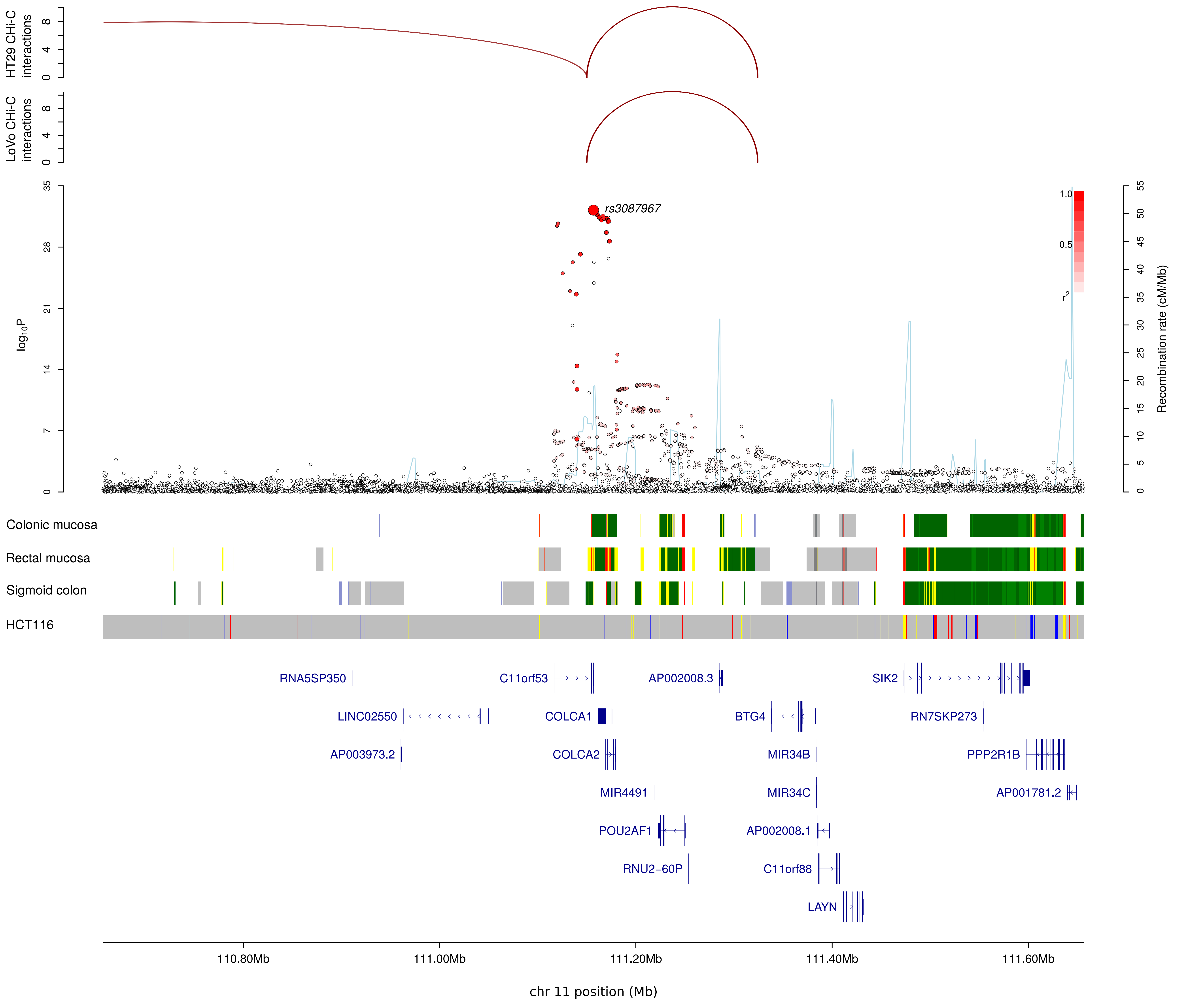

HT29 CHi-C interactions
LoVo CHi-C interactions
rs3087967
−log10P
Recombination rate (cM/Mb)
r²
Colonic mucosa
Rectal mucosa
Sigmoid colon
HCT116
RNA5SP350
C11orf53
AP002008.3
SIK2
LINC02550
COLCA1
BTG4
RN7SKP273
AP003973.2
COLCA2
MIR34B
PPP2R1B
MIR4491
MIR34C
AP001781.2
POU2AF1
AP002008.1
RNU2−60P
C11orf88
LAYN
110.80Mb
111.00Mb
111.20Mb
111.40Mb
111.60Mb
chr 11 position (Mb)

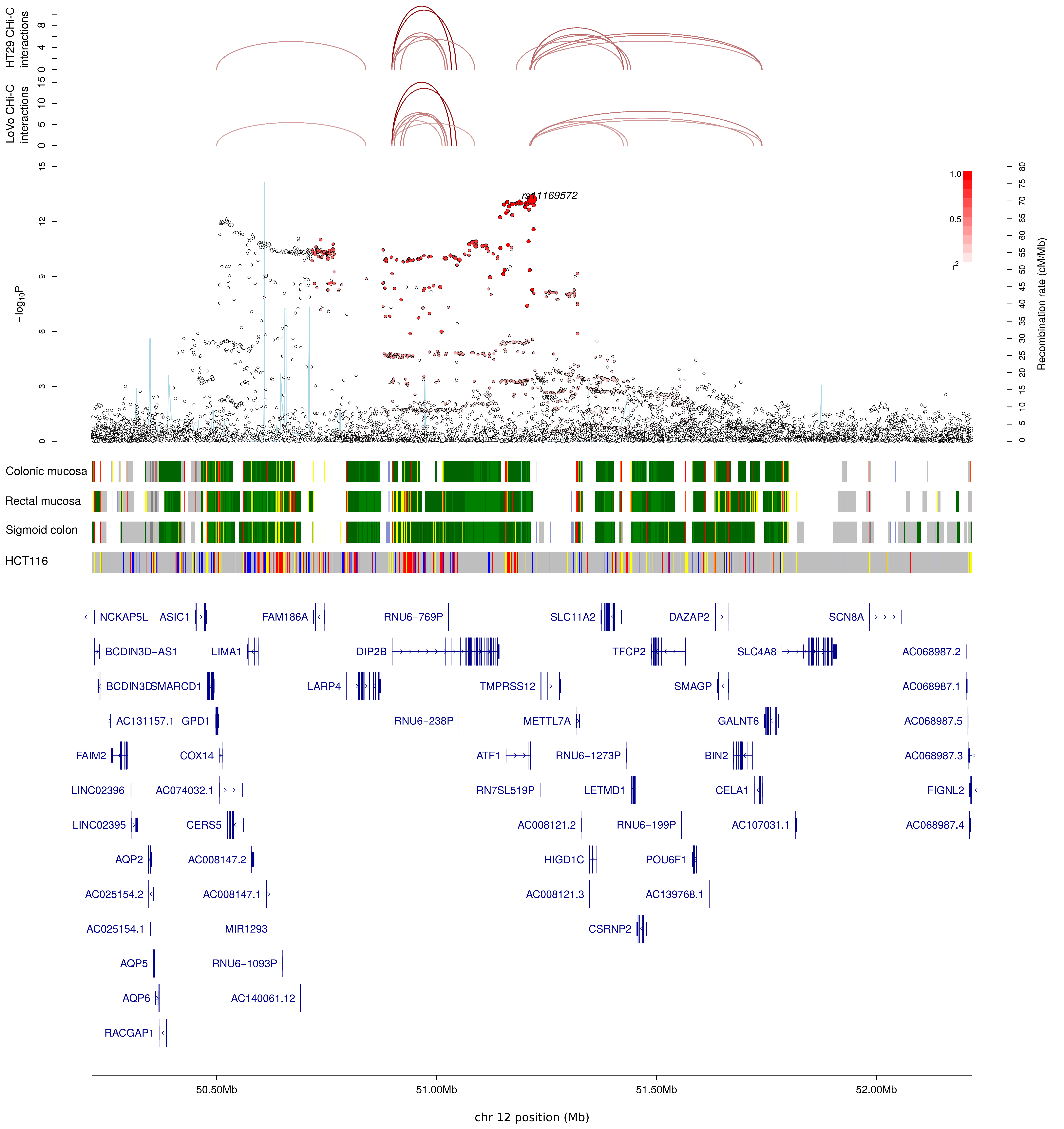

HT29 CHi-C interactions
LoVo CHi-C interactions
−log10P
rs11169572
Recombination rate (cM/Mb)
r2
Colonic mucosa
Rectal mucosa
Sigmoid colon
HCT116
NCKAP5L
ASIC1
FAM186A
RNU6−769P
SLC11A2
DAZAP2
SCN8A
BCDIN3D−AS1
LIMA1
DIP2B
TFCP2
SLC4A8
AC068987.2
BCDIN3D
SMARCD1
LARP4
TMPRSS12
SMAGP
AC068987.1
AC131157.1
GPD1
RNU6−238P
METTL7A
GALNT6
AC068987.5
FAIM2
COX14
ATF1
RNU6−1273P
BIN2
AC068987.3
LINC02396
AC074032.1
RN7SL519P
LETMD1
CELA1
FIGNL2
LINC02395
CERS5
AC008121.2
RNU6−199P
AC107031.1
AC068987.4
AQP2
AC008147.2
HIGD1C
POU6F1
AC025154.2
AC008147.1
AC008121.3
AC139768.1
AC025154.1
MIR1293
CSRNP2
AQP5
RNU6−1093P
AQP6
AC140061.12
RACGAP1
50.50Mb
51.00Mb
51.50Mb
52.00Mb
chr 12 position (Mb)

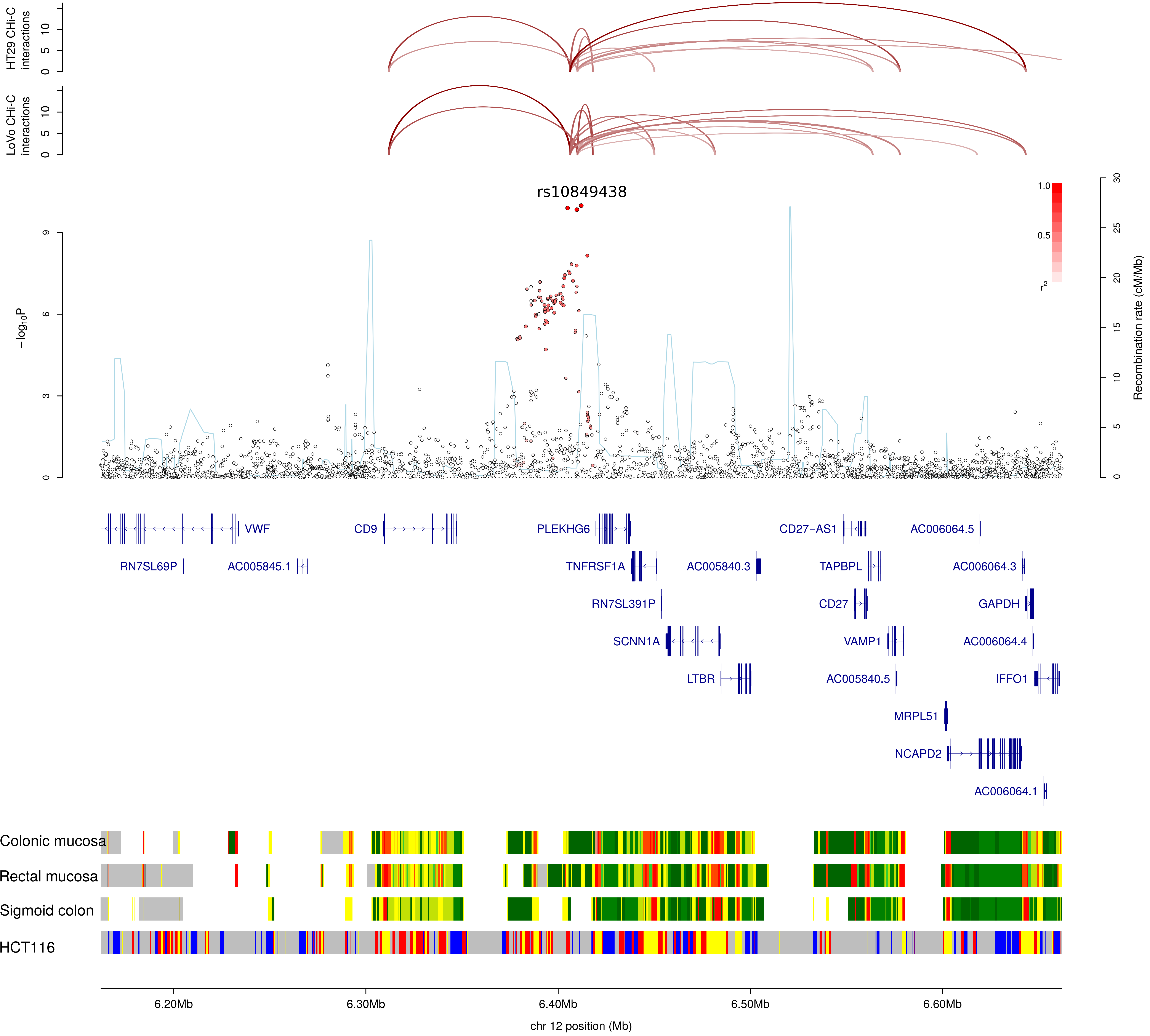
HT29 CHi-C interactions
LoVo CHi-C interactions
rs10849438
−log10P
Recombination rate (cM/Mb)
r2
1.0
0.5
VWF
CD9
PLEKHG6
CD27−AS1
AC006064.5
RN7SL69P
AC005845.1
TNFRSF1A
AC005840.3
TAPBPL
AC006064.3
RN7SL391P
CD27
GAPDH
SCNN1A
VAMP1
AC006064.4
LTBR
AC005840.5
IFFO1
MRPL51
NCAPD2
AC006064.1
Colonic mucosa
Rectal mucosa
Sigmoid colon
HCT116
6.20Mb
6.30Mb
6.40Mb
6.50Mb
6.60Mb
chr 12 position (Mb)

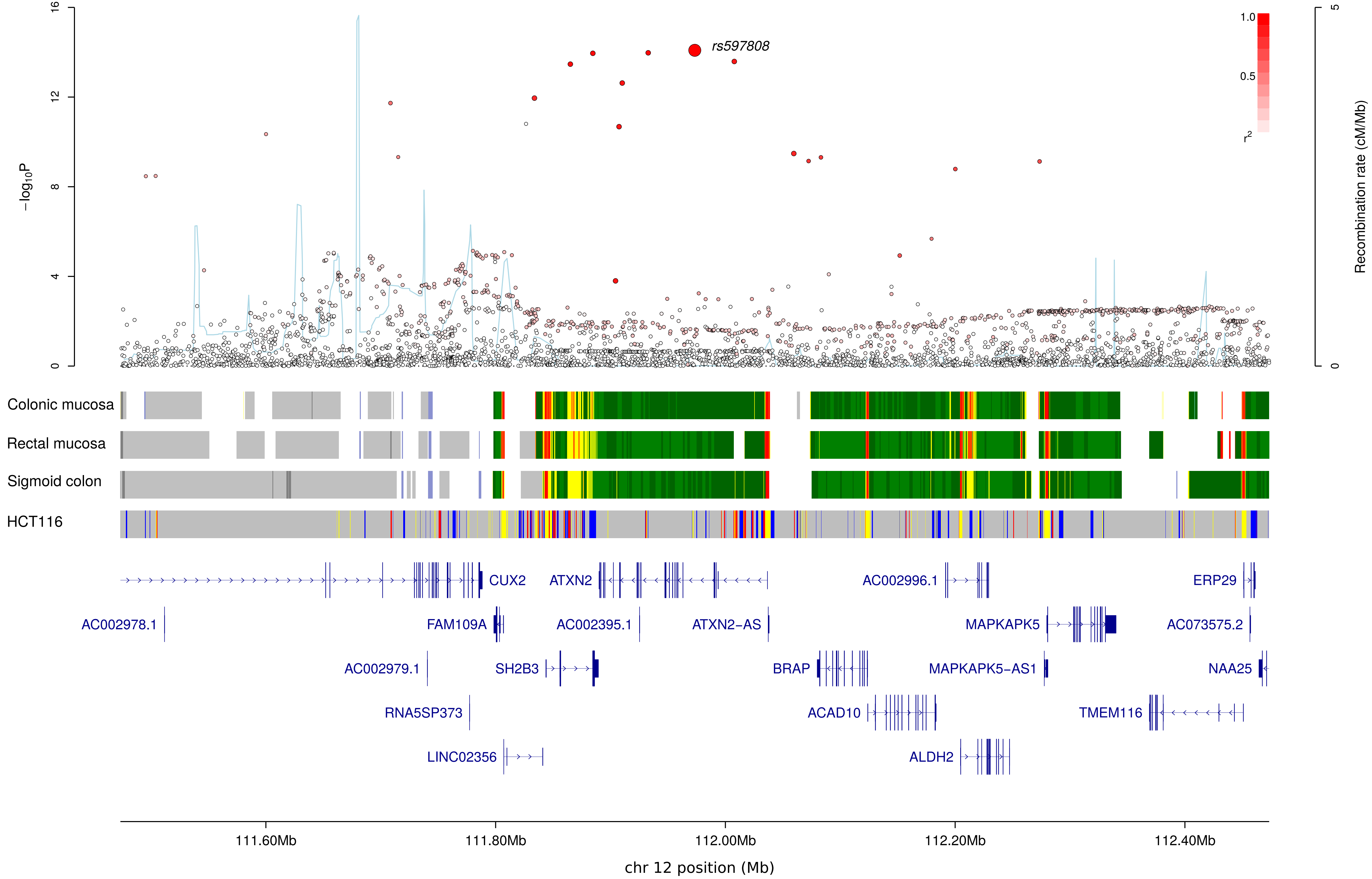

rs597808
1.0
0.5
r^2
$-\log_{10}P$
16
12
8
4
0
5
0
Recombination rate (cM/Mb)
Colonic mucosa
Rectal mucosa
Sigmoid colon
HCT116
CUX2
ATXN2
AC002996.1
ERP29
AC002978.1
FAM109A
AC002395.1
ATXN2-AS
MAPKAPK5
AC073575.2
AC002979.1
SH2B3
BRAP
MAPKAPK5-AS1
NAA25
RNA5SP373
ACAD10
TMEM116
LINC02356
ALDH2
111.60Mb
111.80Mb
112.00Mb
112.20Mb
112.40Mb
chr 12 position (Mb)

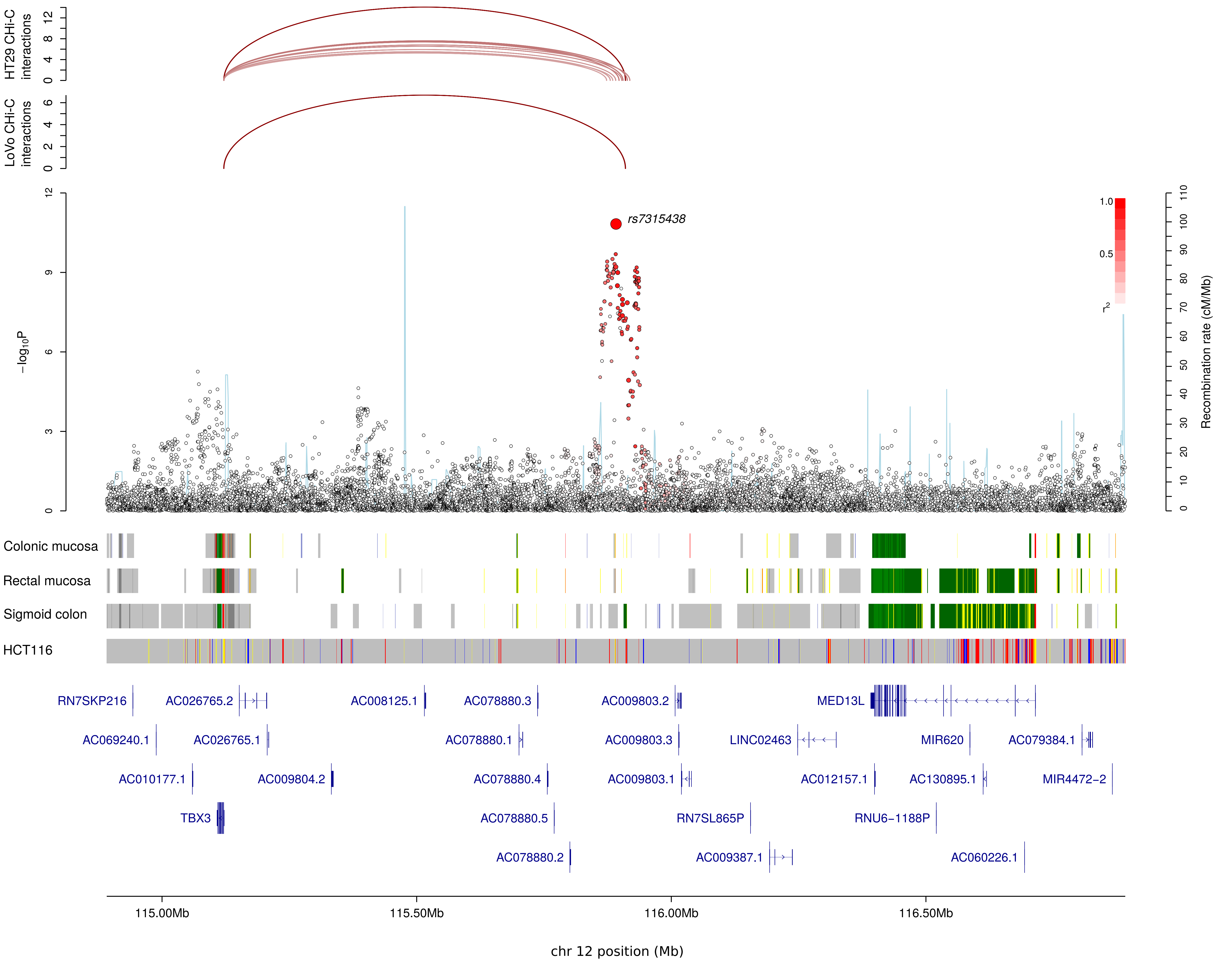

HT29 CHi-C interactions
LoVo CHi-C interactions
rs7315438
−log10P
Recombination rate (cM/Mb)
r2
Colonic mucosa
Rectal mucosa
Sigmoid colon
HCT116
RN7SKP216
AC026765.2
AC008125.1
AC078880.3
AC009803.2
MED13L
AC069240.1
AC026765.1
AC078880.1
AC009803.3
LINC02463
MIR620
AC079384.1
AC010177.1
AC009804.2
AC078880.4
AC009803.1
AC012157.1
AC130895.1
MIR4472−2
TBX3
AC078880.5
RN7SL865P
RNU6−1188P
AC078880.2
AC009387.1
AC060226.1
115.00Mb
115.50Mb
116.00Mb
116.50Mb
chr 12 position (Mb)

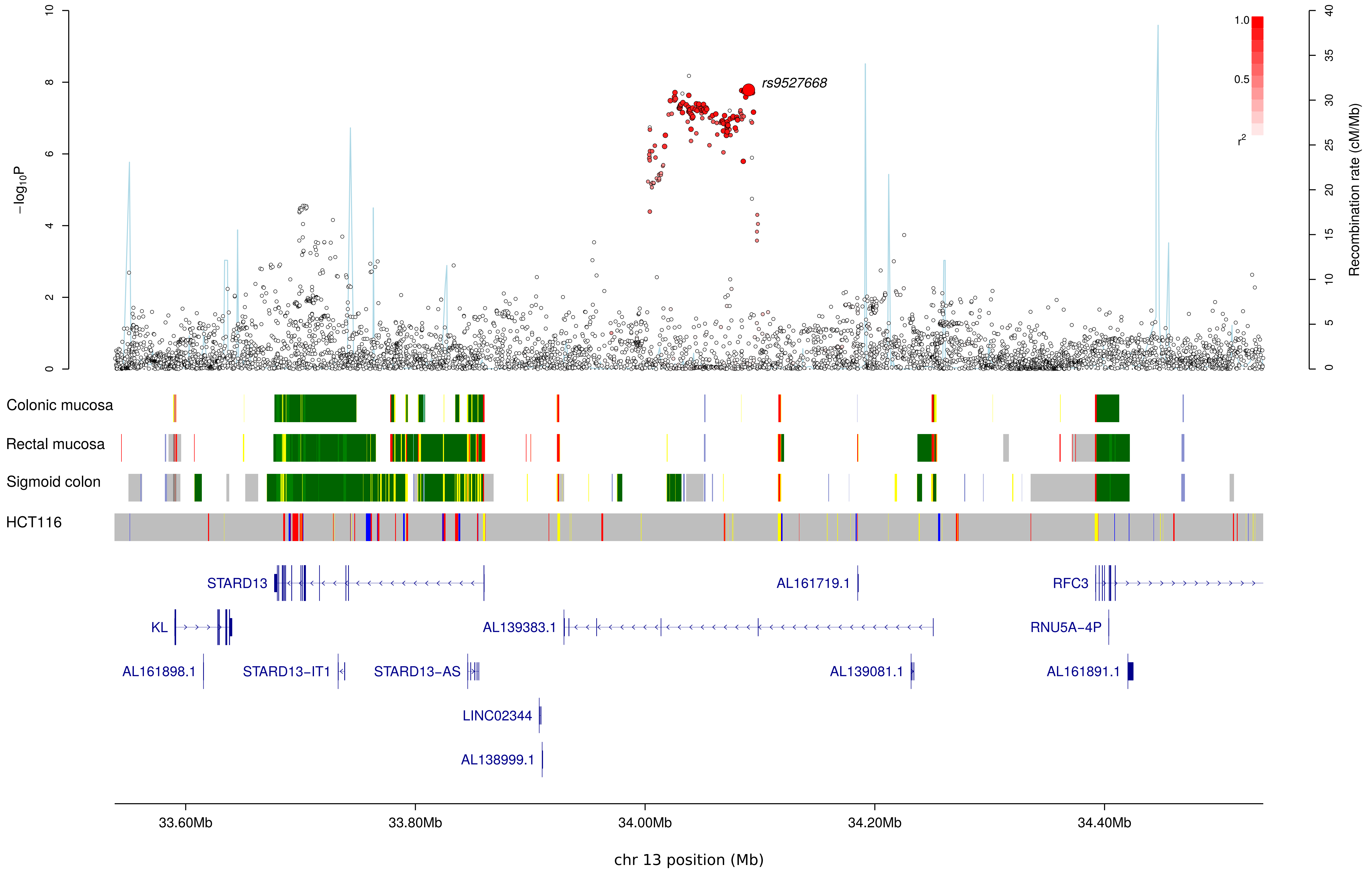

rs9527668
1.0
0.5
r^2
$-\log_{10}P$
Recombination rate (cM/Mb)
Colonic mucosa
Rectal mucosa
Sigmoid colon
HCT116
STARD13
AL161719.1
RFC3
KL
AL139383.1
RNU5A-4P
AL161898.1
STARD13-IT1
STARD13-AS
AL139081.1
AL161891.1
LINC02344
AL138999.1
33.60Mb
33.80Mb
34.00Mb
34.20Mb
34.40Mb
chr 13 position (Mb)

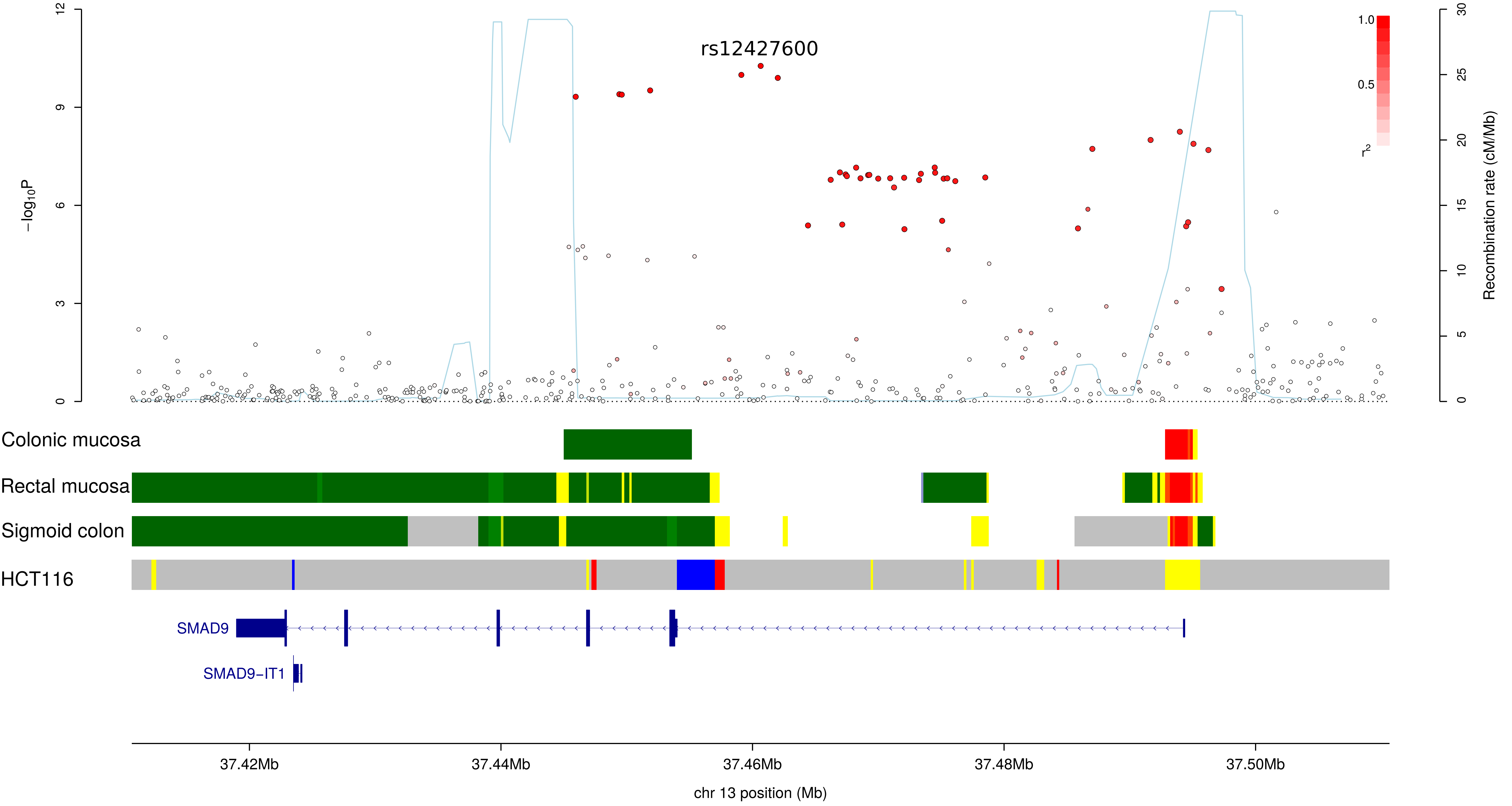
rs12427600
−log10P
Recombination rate (cM/Mb)
1.0
0.5
r2
Colonic mucosa
Rectal mucosa
Sigmoid colon
HCT116
SMAD9
SMAD9−IT1
37.42Mb
37.44Mb
37.46Mb
37.48Mb
37.50Mb
chr 13 position (Mb)

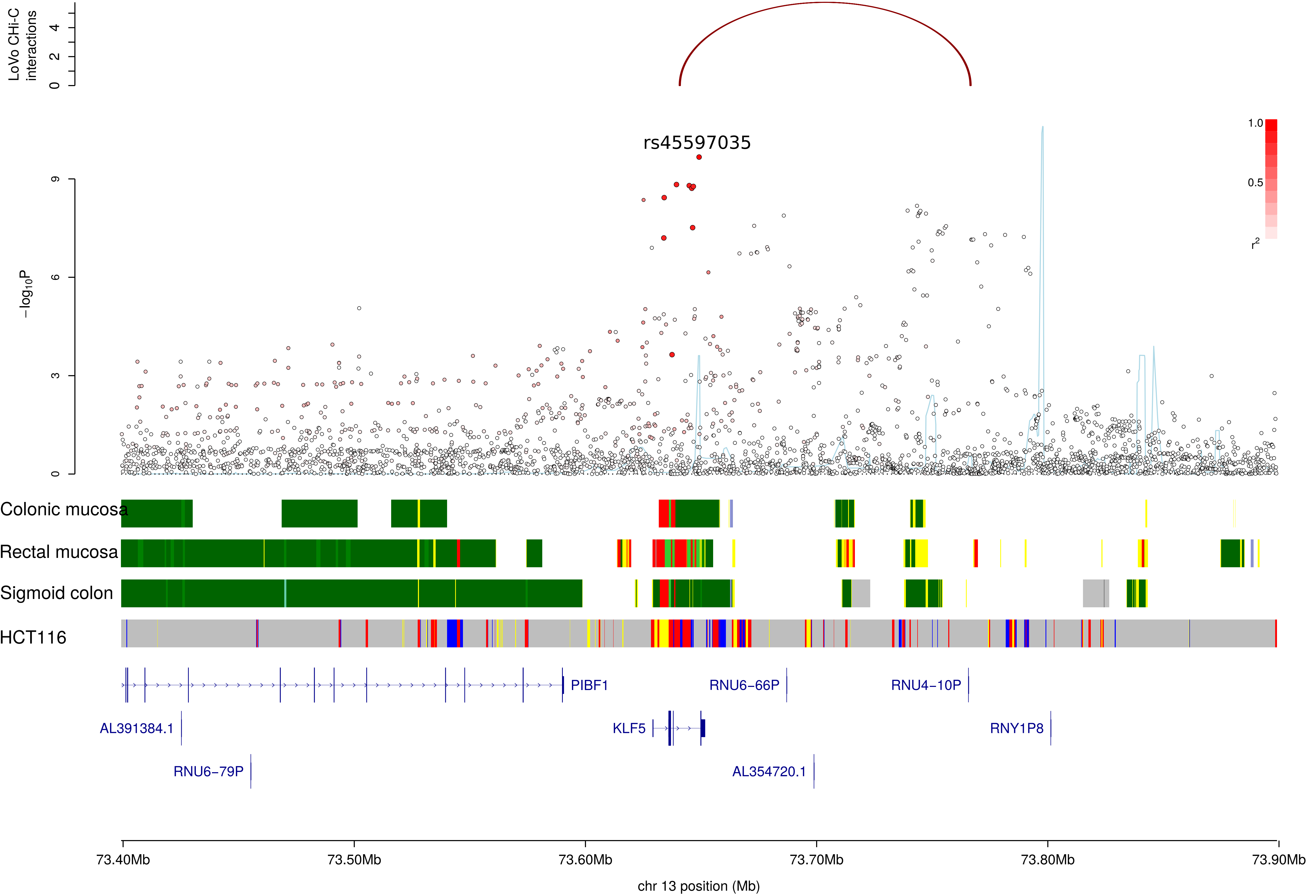

LoVo CHi-C interactions
0
2
4
rs45597035
$-\log_{10}P$
0
3
6
9
1.0
0.5
r^2
Recombination rate (cM/Mb)
0
5
10
15
20
25
30
35
40
45
50
Colonic mucosa
Rectal mucosa
Sigmoid colon
HCT116
PIBF1
RNU6-66P
RNU4-10P
AL391384.1
KLF5
RNY1P8
RNU6-79P
AL354720.1
73.40Mb
73.50Mb
73.60Mb
73.70Mb
73.80Mb
73.90Mb
chr 13 position (Mb)

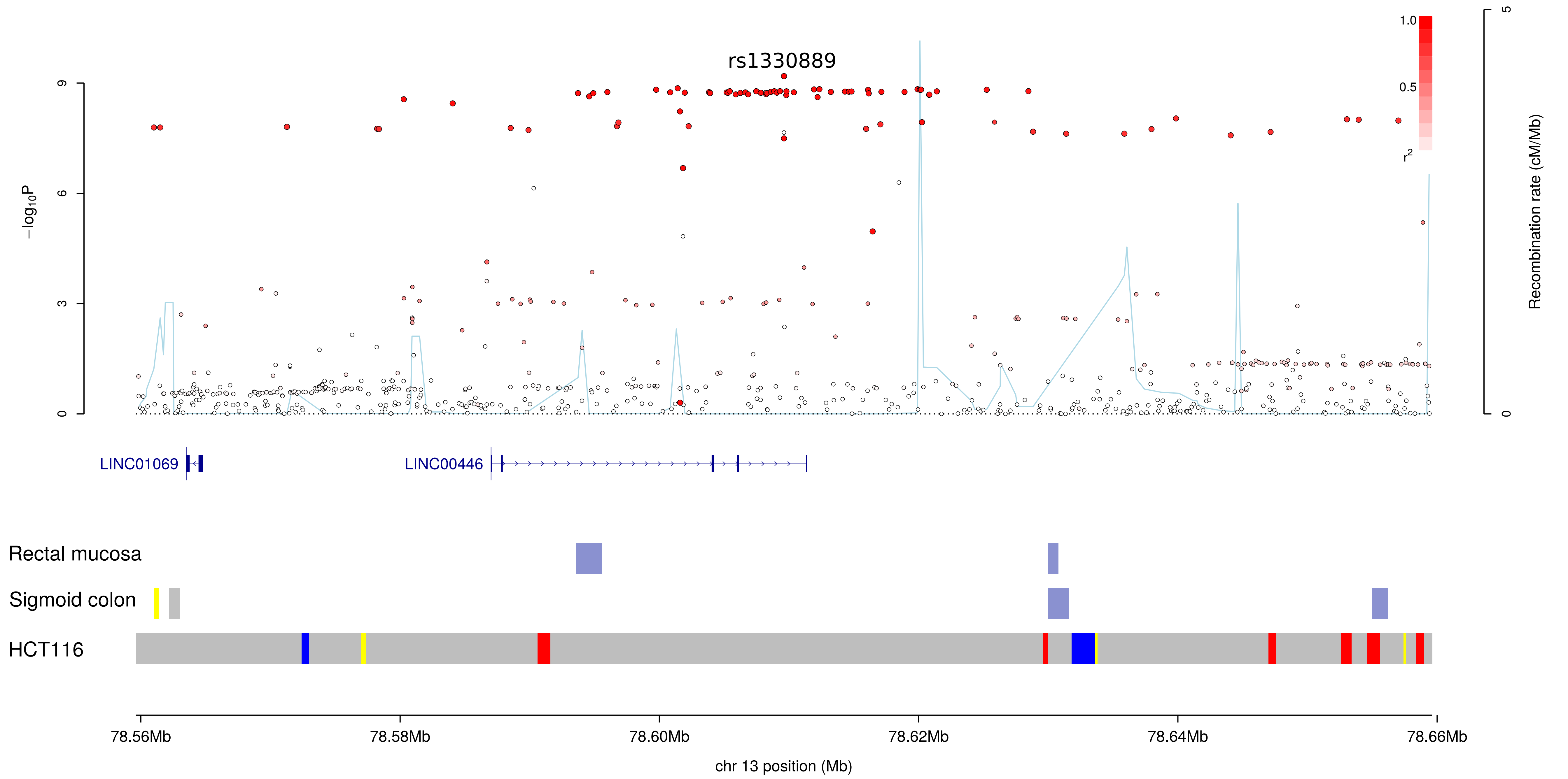

rs1330889
−log10P
9
6
3
0
1.0
0.5
r2
5
0
Recombination rate (cM/Mb)
LINC01069
LINC00446
Rectal mucosa
Sigmoid colon
HCT116
78.56Mb
78.58Mb
78.60Mb
78.62Mb
78.64Mb
78.66Mb
chr 13 position (Mb)

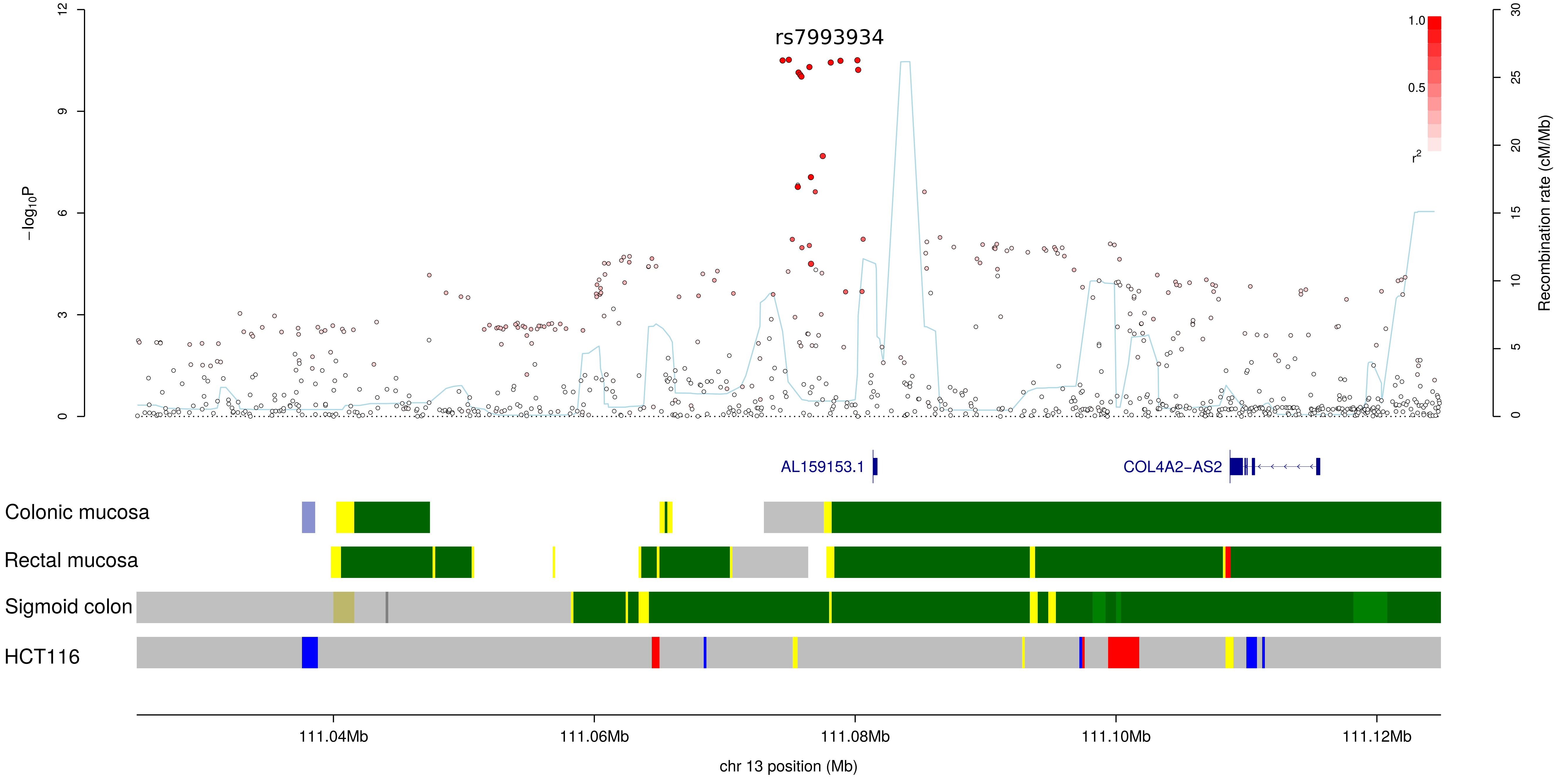

rs7993934
−log10P
Recombination rate (cM/Mb)
1.0
0.5
r2
AL159153.1
COL4A2−AS2
Colonic mucosa
Rectal mucosa
Sigmoid colon
HCT116
111.04Mb
111.06Mb
111.08Mb
111.10Mb
111.12Mb
chr 13 position (Mb)

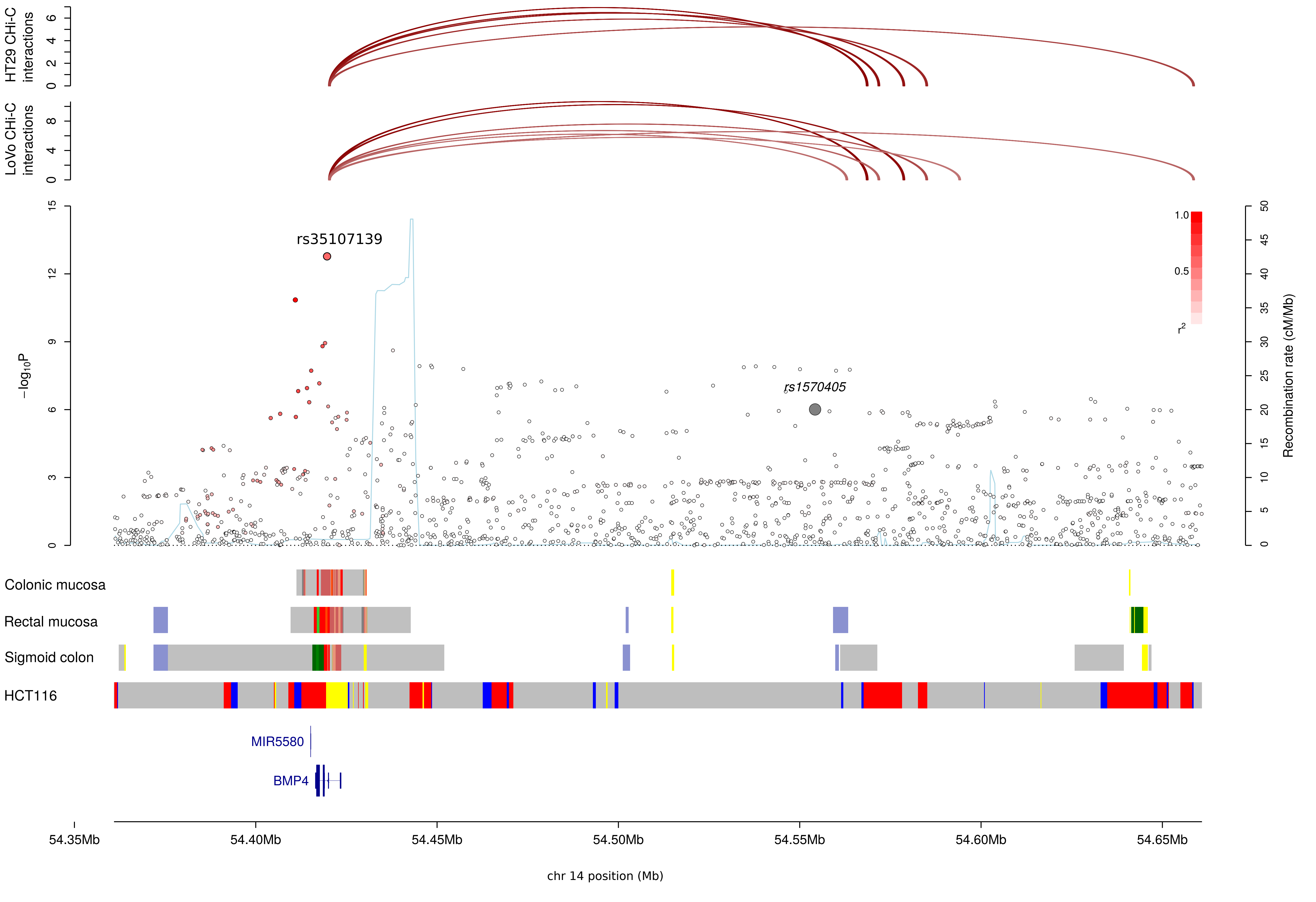
HT29 CHi-C interactions
LoVo CHi-C interactions
rs35107139
rs1570405
−log10P
Recombination rate (cM/Mb)
1.0
0.5
r2
Colonic mucosa
Rectal mucosa
Sigmoid colon
HCT116
MIR5580
BMP4
54.35Mb
54.40Mb
54.45Mb
54.50Mb
54.55Mb
54.60Mb
54.65Mb
chr 14 position (Mb)

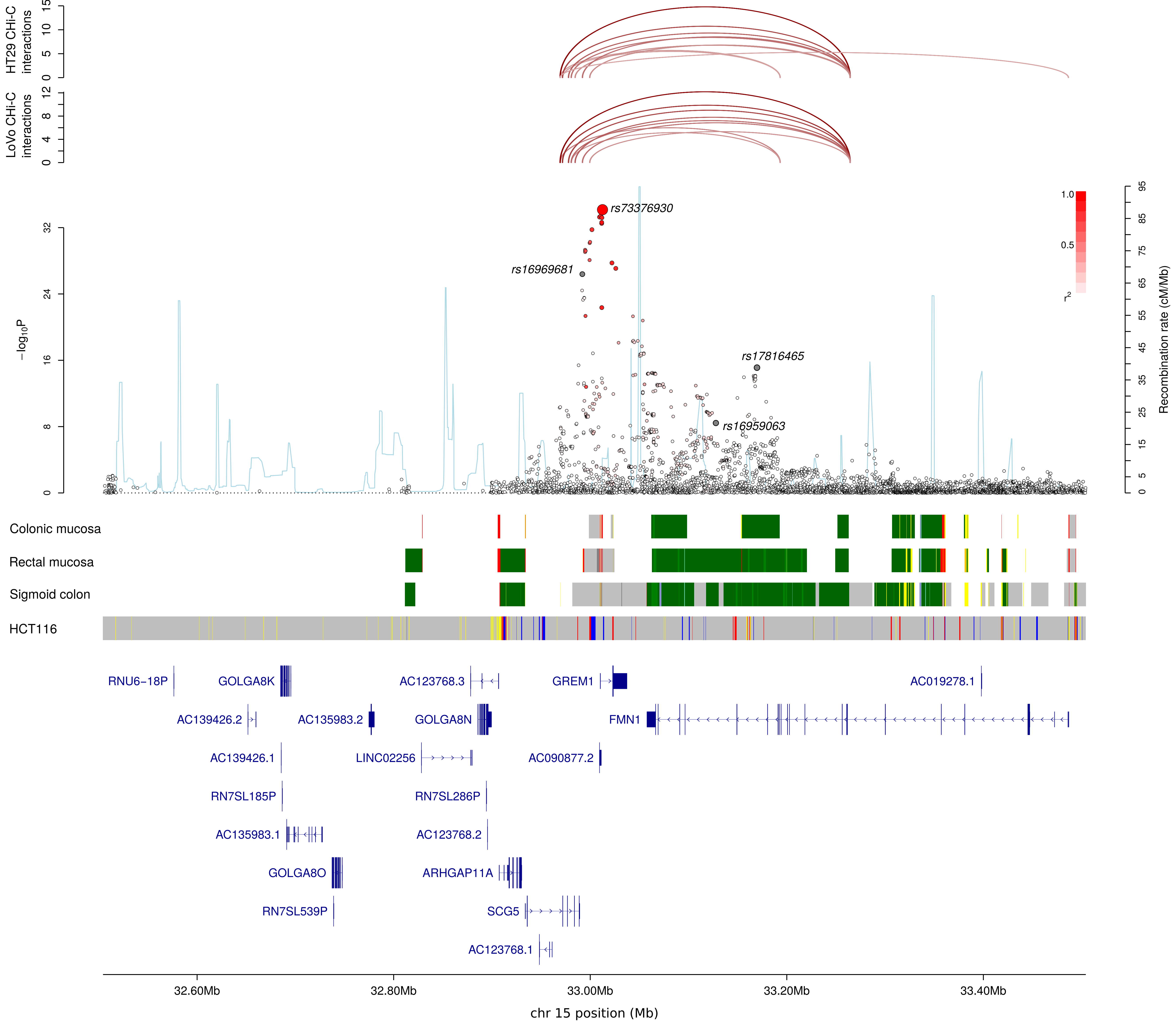
HT29 CHi-C interactions
LoVo CHi-C interactions
−log10P
Recombination rate (cM/Mb)
r2
rs73376930
rs16969681
rs17816465
rs16959063
Colonic mucosa
Rectal mucosa
Sigmoid colon
HCT116
RNU6-18P
GOLGA8K
AC123768.3
GREM1
AC019278.1
AC139426.2
AC135983.2
GOLGA8N
FMN1
AC139426.1
LINC02256
AC090877.2
RN7SL185P
RN7SL286P
AC135983.1
AC123768.2
GOLGA8O
ARHGAP11A
RN7SL539P
SCG5
AC123768.1
32.60Mb
32.80Mb
33.00Mb
33.20Mb
33.40Mb
chr 15 position (Mb)

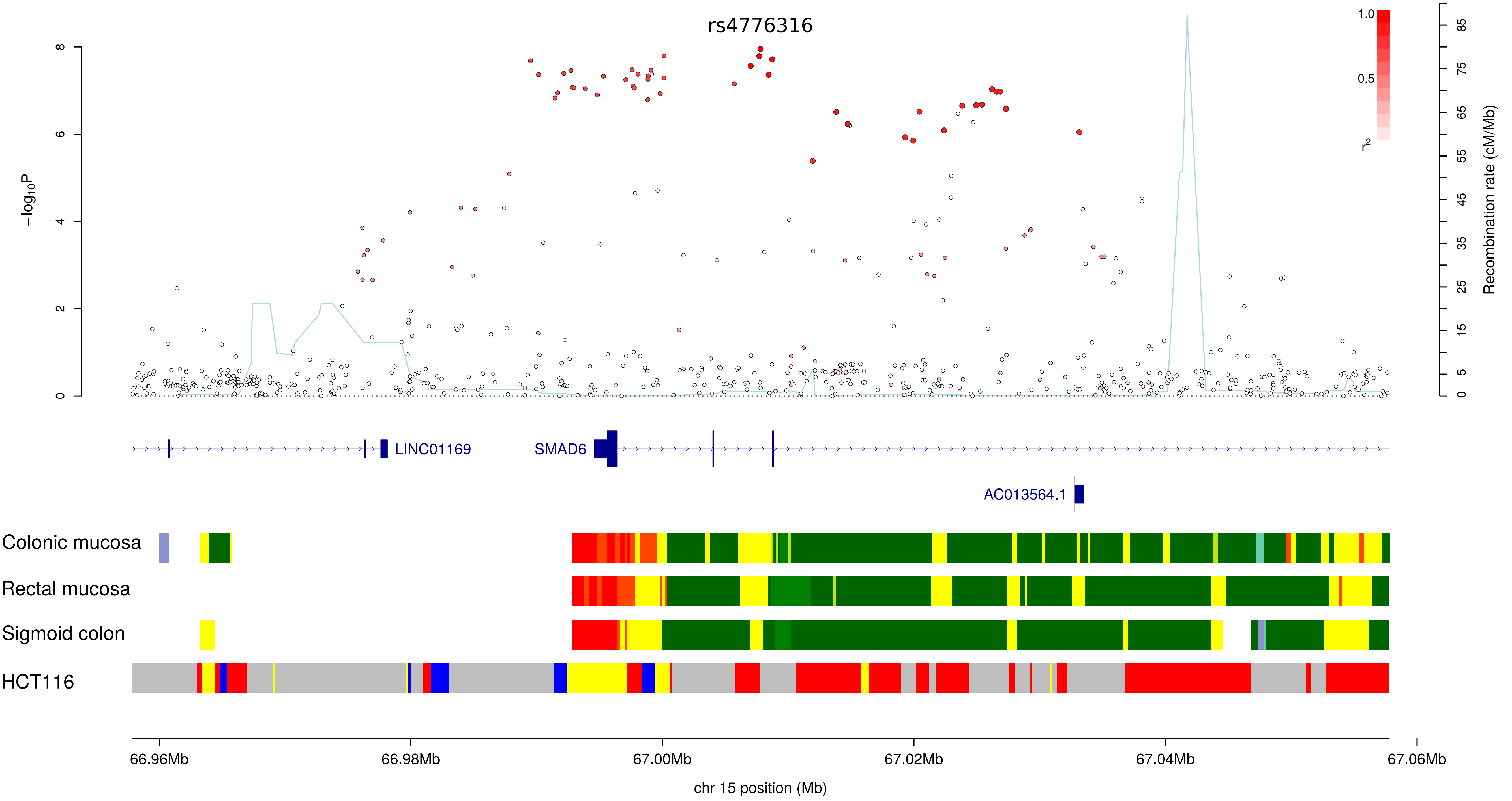

rs4776316
−log10P
0
2
4
6
8
1.0
0.5
r2
Recombination rate (cM/Mb)
0
5
15
25
35
45
55
65
75
85
LINC01169
SMAD6
AC013564.1
Colonic mucosa
Rectal mucosa
Sigmoid colon
HCT116
66.96Mb
66.98Mb
67.00Mb
67.02Mb
67.04Mb
67.06Mb
chr 15 position (Mb)

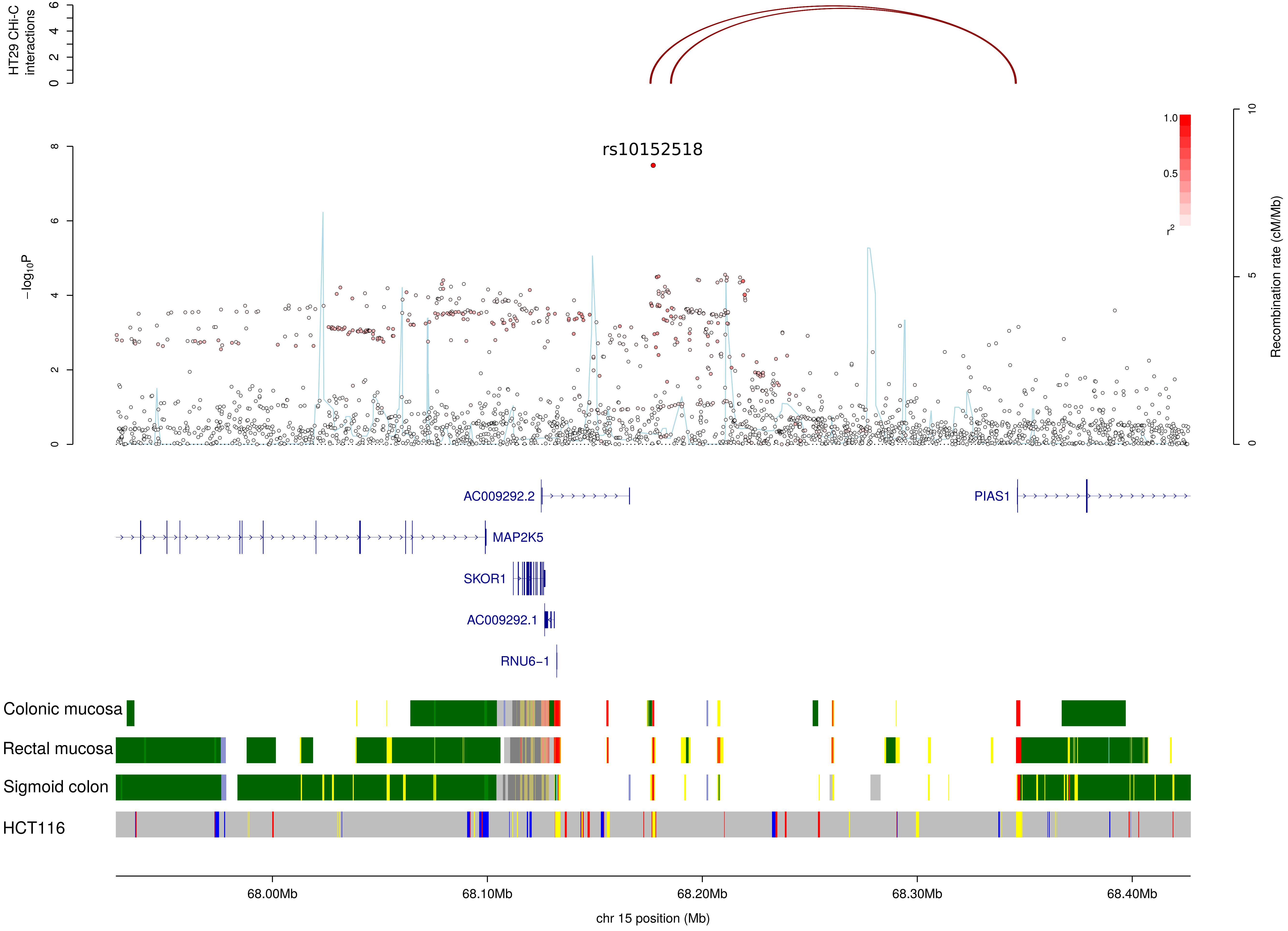
HT29 CHi-C interactions
0
2
4
6
rs10152518
−log10P
0
2
4
6
8
1.0
0.5
r2
Recombination rate (cM/Mb)
0
5
10
AC009292.2
PIAS1
MAP2K5
SKOR1
AC009292.1
RNU6−1
Colonic mucosa
Rectal mucosa
Sigmoid colon
HCT116
68.00Mb
68.10Mb
68.20Mb
68.30Mb
68.40Mb
chr 15 position (Mb)

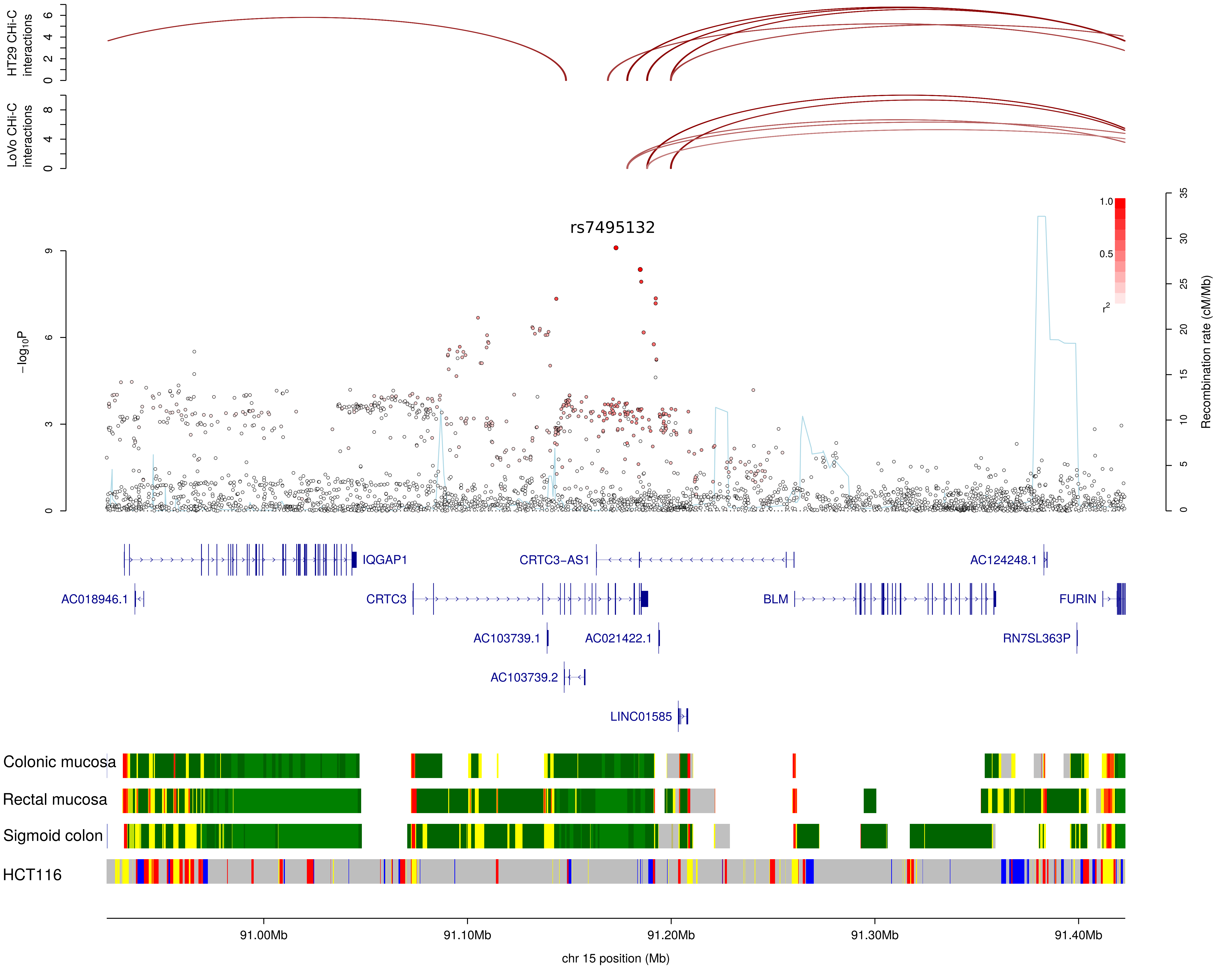
HT29 CHi-C interactions
LoVo CHi-C interactions
rs7495132
−log10P
Recombination rate (cM/Mb)
r2
IQGAP1
CRTC3-AS1
AC124248.1
AC018946.1
CRTC3
BLM
FURIN
AC103739.1
AC021422.1
RN7SL363P
AC103739.2
LINC01585
Colonic mucosa
Rectal mucosa
Sigmoid colon
HCT116
91.00Mb
91.10Mb
91.20Mb
91.30Mb
91.40Mb
chr 15 position (Mb)

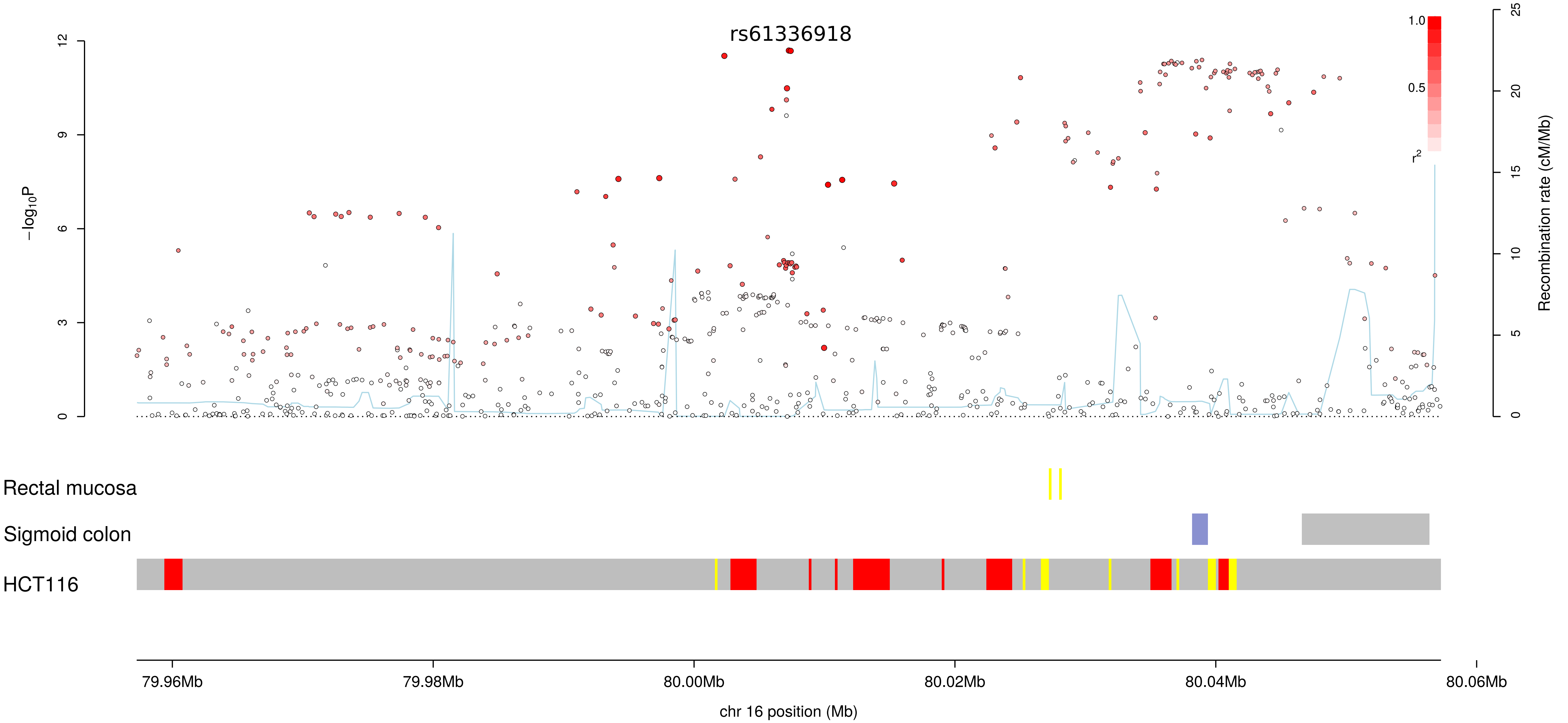

rs61336918
−log10P
0
3
6
9
12
Recombination rate (cM/Mb)
0
5
10
15
20
25
1.0
0.5
r2
Rectal mucosa
Sigmoid colon
HCT116
79.96Mb
79.98Mb
80.00Mb
80.02Mb
80.04Mb
80.06Mb
chr 16 position (Mb)

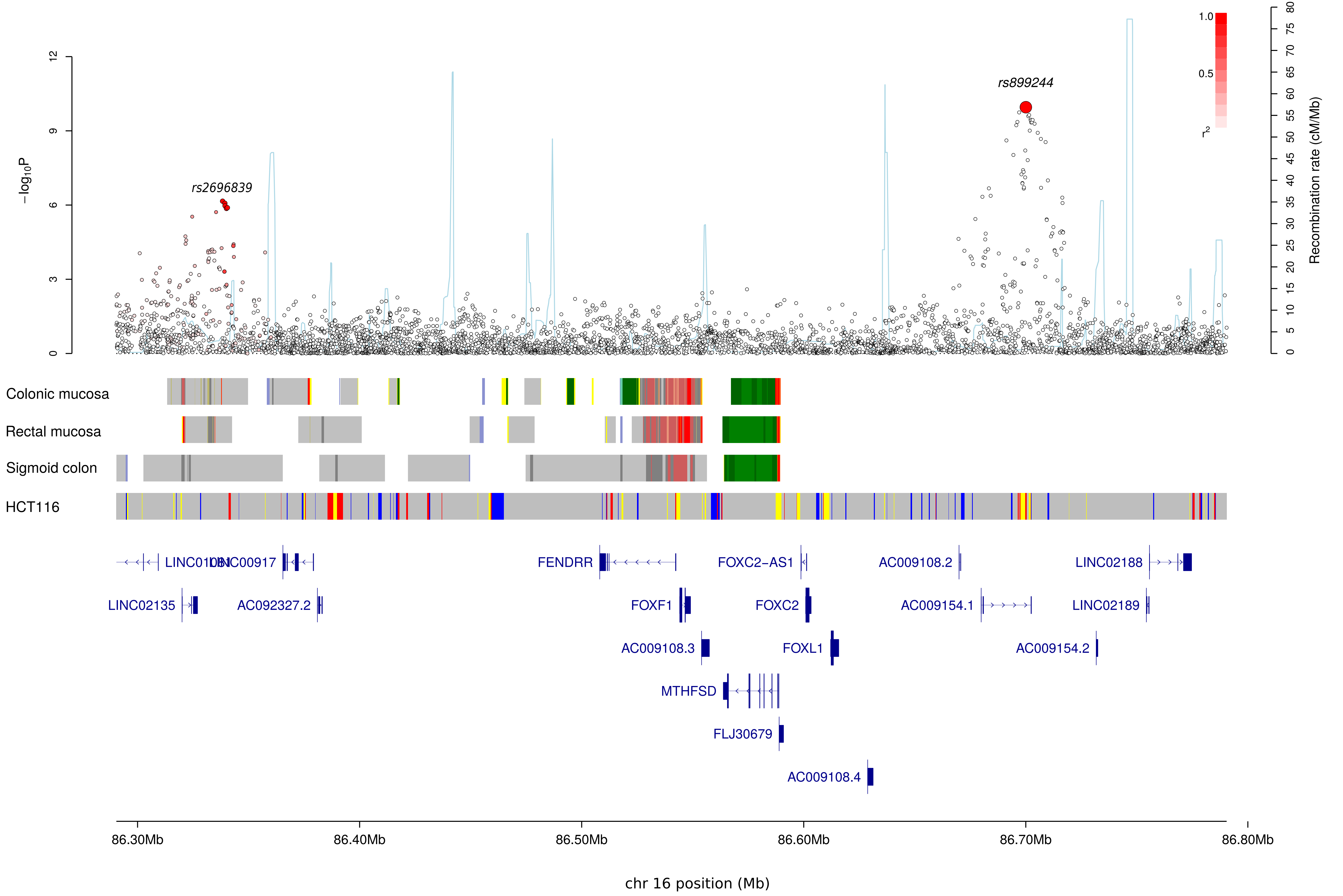

rs899244
rs2696839
−log10P
Recombination rate (cM/Mb)
r2
1.0
0.5
Colonic mucosa
Rectal mucosa
Sigmoid colon
HCT116
LINC01081
LINC00917
FENDRR
FOXC2−AS1
AC009108.2
LINC02188
LINC02135
AC092327.2
FOXF1
FOXC2
AC009154.1
LINC02189
AC009108.3
FOXL1
AC009154.2
MTHFSD
FLJ30679
AC009108.4
86.30Mb
86.40Mb
86.50Mb
86.60Mb
86.70Mb
86.80Mb
chr 16 position (Mb)

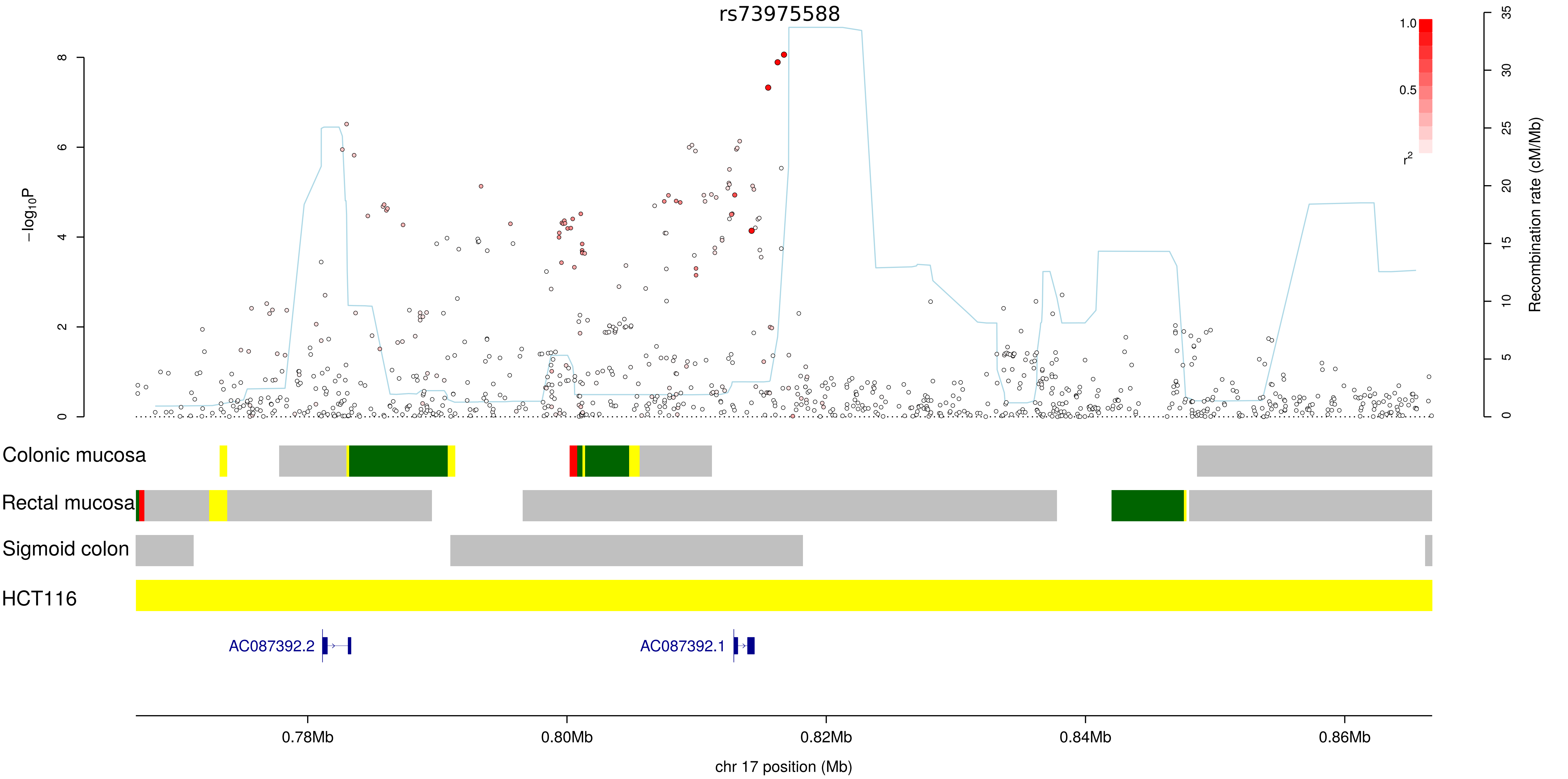
rs73975588
$-\log_{10}P$
Recombination rate (cM/Mb)
r^2
1.0
0.5
Colonic mucosa
Rectal mucosa
Sigmoid colon
HCT116
AC087392.2
AC087392.1
0.78Mb
0.80Mb
0.82Mb
0.84Mb
0.86Mb
chr 17 position (Mb)

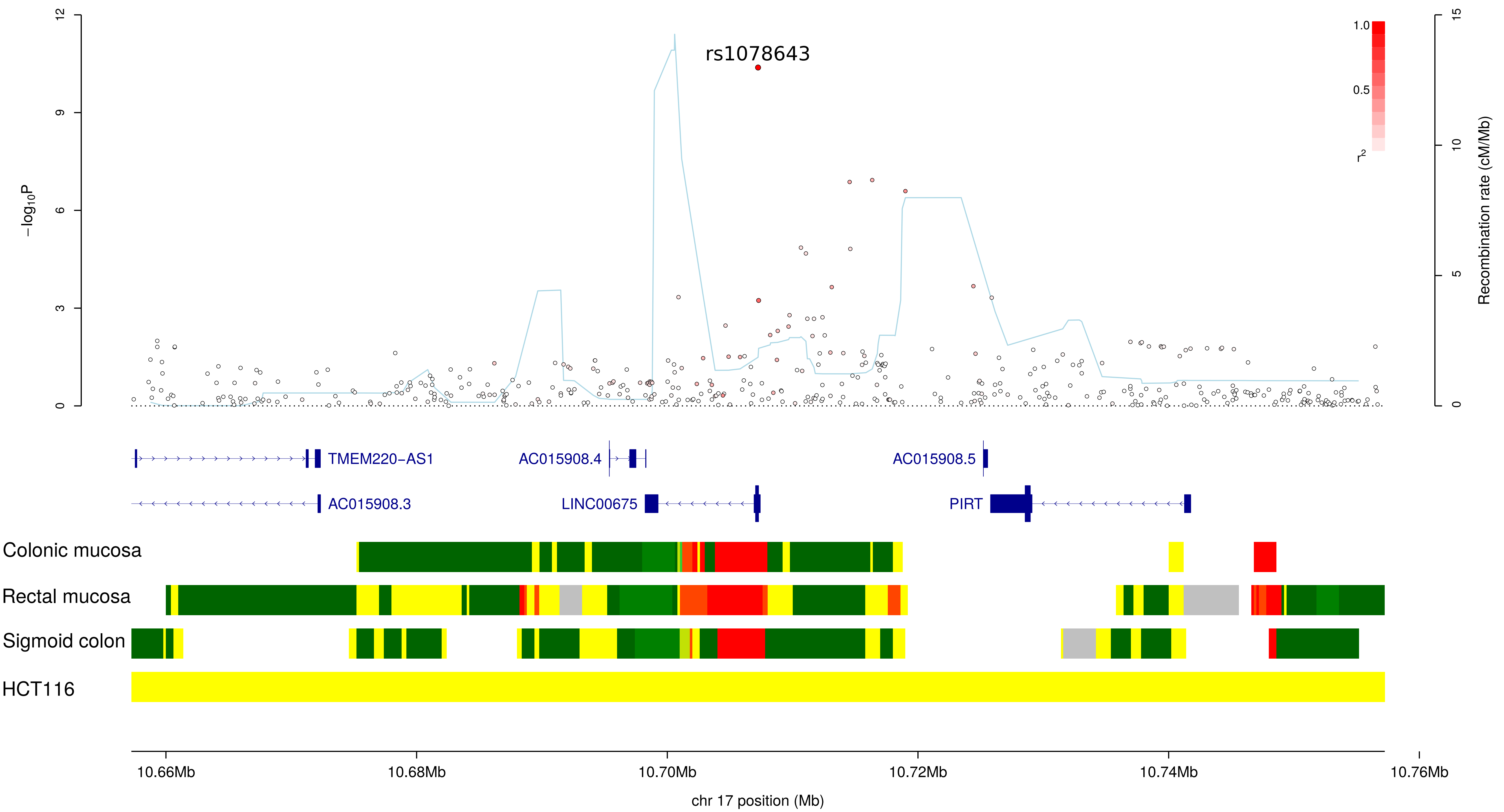

rs1078643
−log10P
Recombination rate (cM/Mb)
1.0
0.5
r2
TMEM220−AS1
AC015908.3
AC015908.4
LINC00675
AC015908.5
PIRT
Colonic mucosa
Rectal mucosa
Sigmoid colon
HCT116
10.66Mb
10.68Mb
10.70Mb
10.72Mb
10.74Mb
10.76Mb
chr 17 position (Mb)

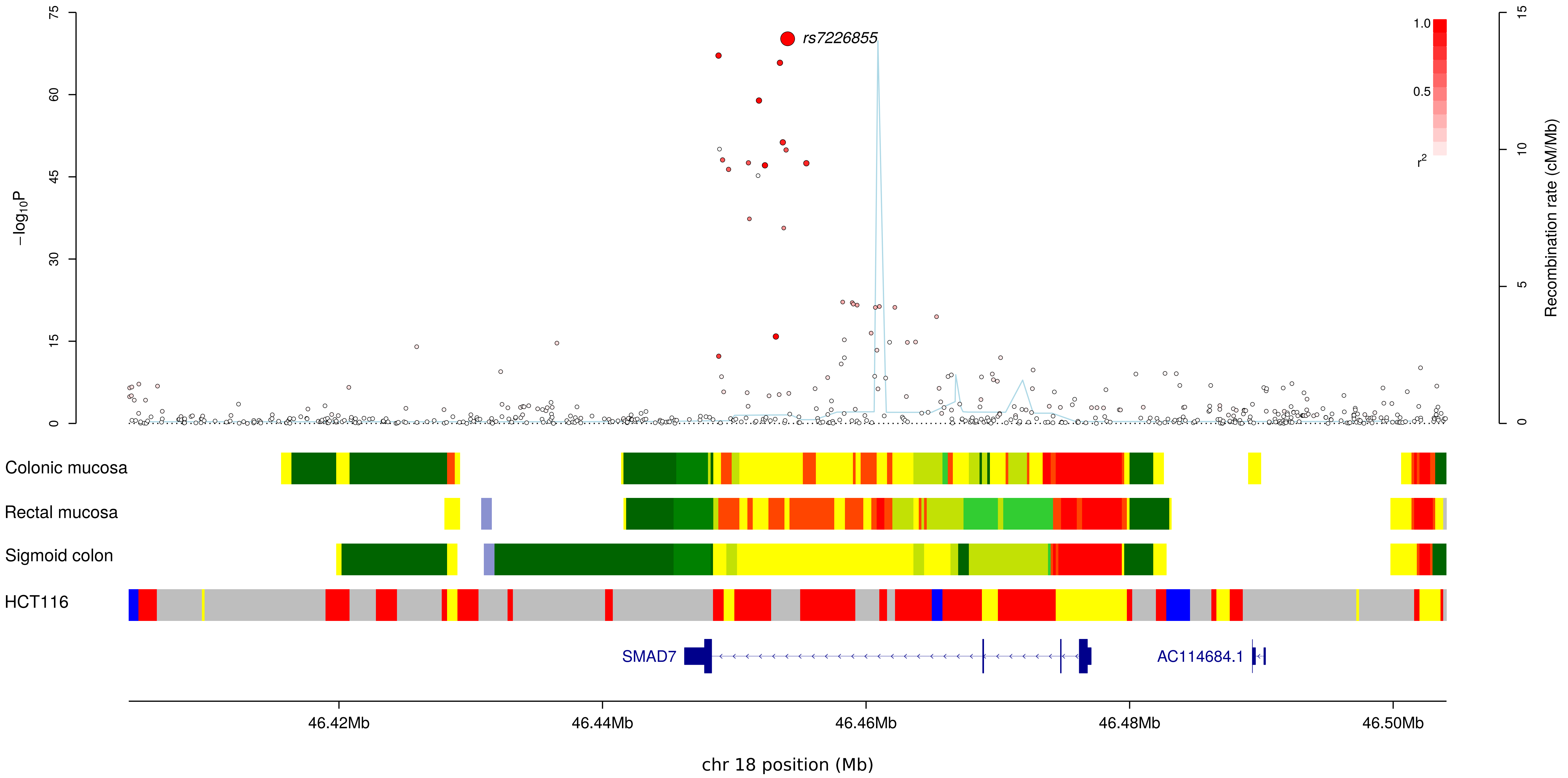

rs7226855
−log10P
75
60
45
30
15
0
1.0
0.5
r2
15
10
5
0
Recombination rate (cM/Mb)
Colonic mucosa
Rectal mucosa
Sigmoid colon
HCT116
SMAD7
AC114684.1
46.42Mb
46.44Mb
46.46Mb
46.48Mb
46.50Mb
chr 18 position (Mb)

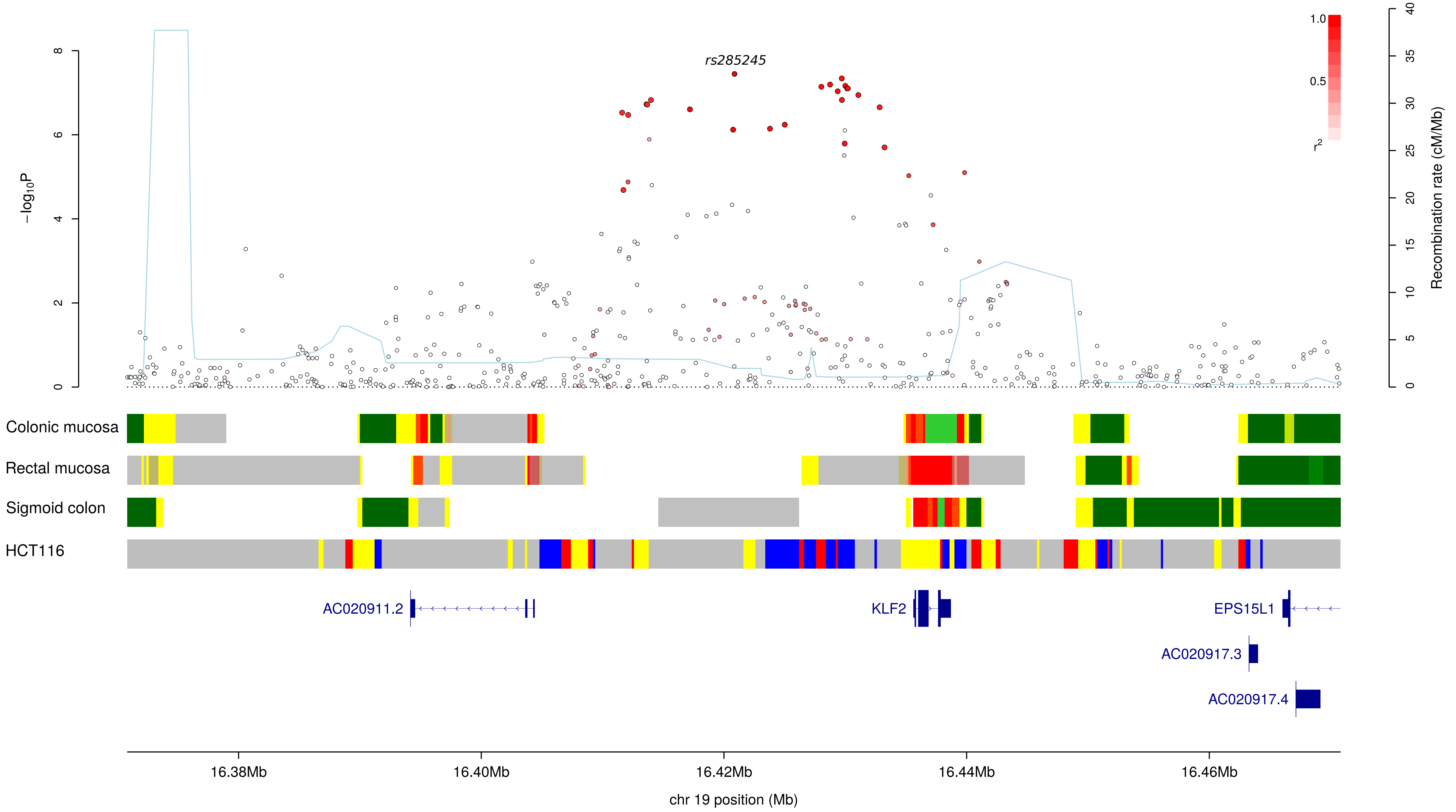
rs285245
1.0
0.5
r^2
$-\log_{10}P$
Recombination rate (cM/Mb)
Colonic mucosa
Rectal mucosa
Sigmoid colon
HCT116
AC020911.2
KLF2
EPS15L1
AC020917.3
AC020917.4
16.38Mb
16.40Mb
16.42Mb
16.44Mb
16.46Mb
chr 19 position (Mb)

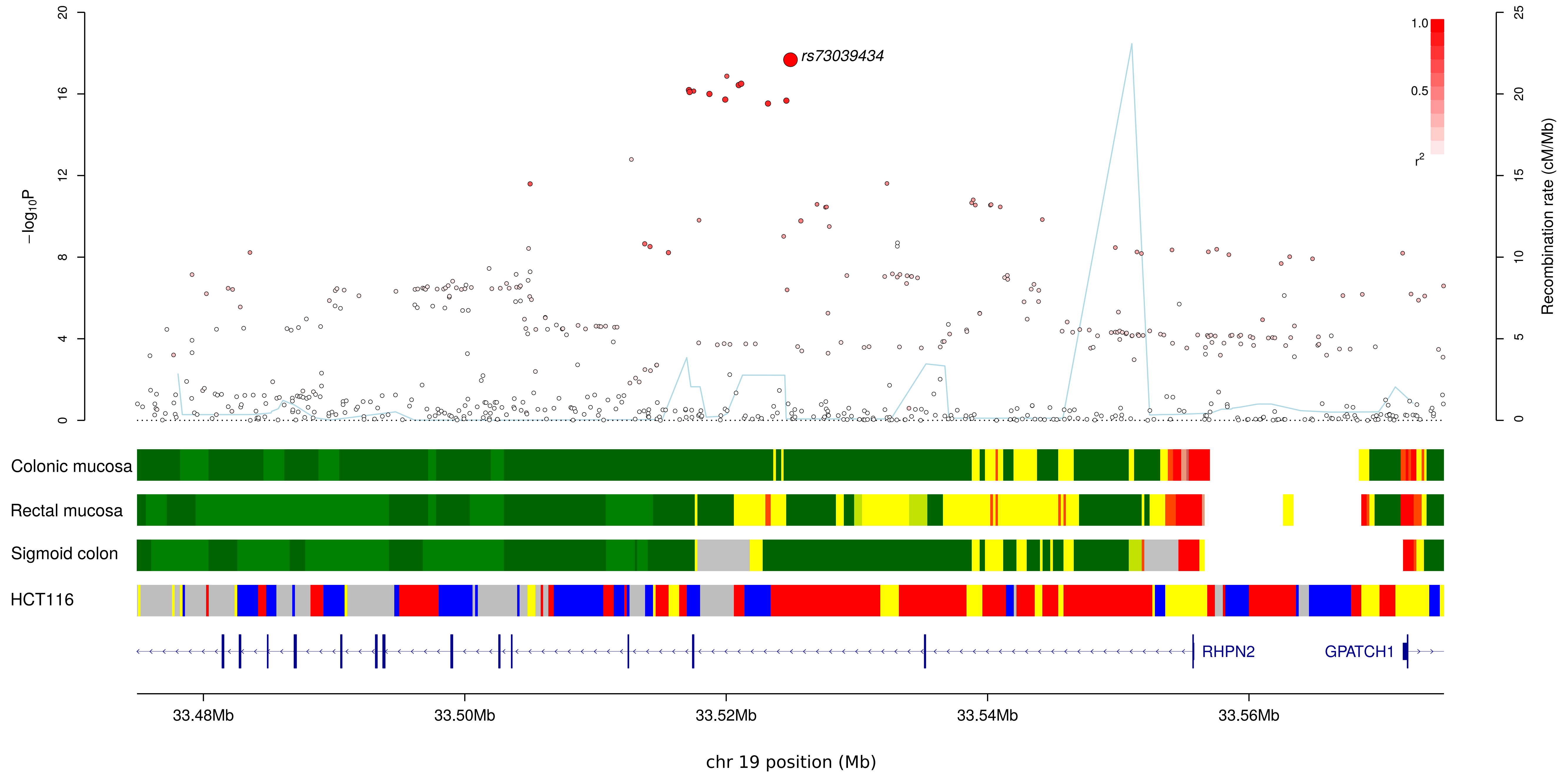

rs73039434
1.0
0.5
r2
−log10P
Recombination rate (cM/Mb)
Colonic mucosa
Rectal mucosa
Sigmoid colon
HCT116
RHPN2
GPATCH1
33.48Mb
33.50Mb
33.52Mb
33.54Mb
33.56Mb
chr 19 position (Mb)

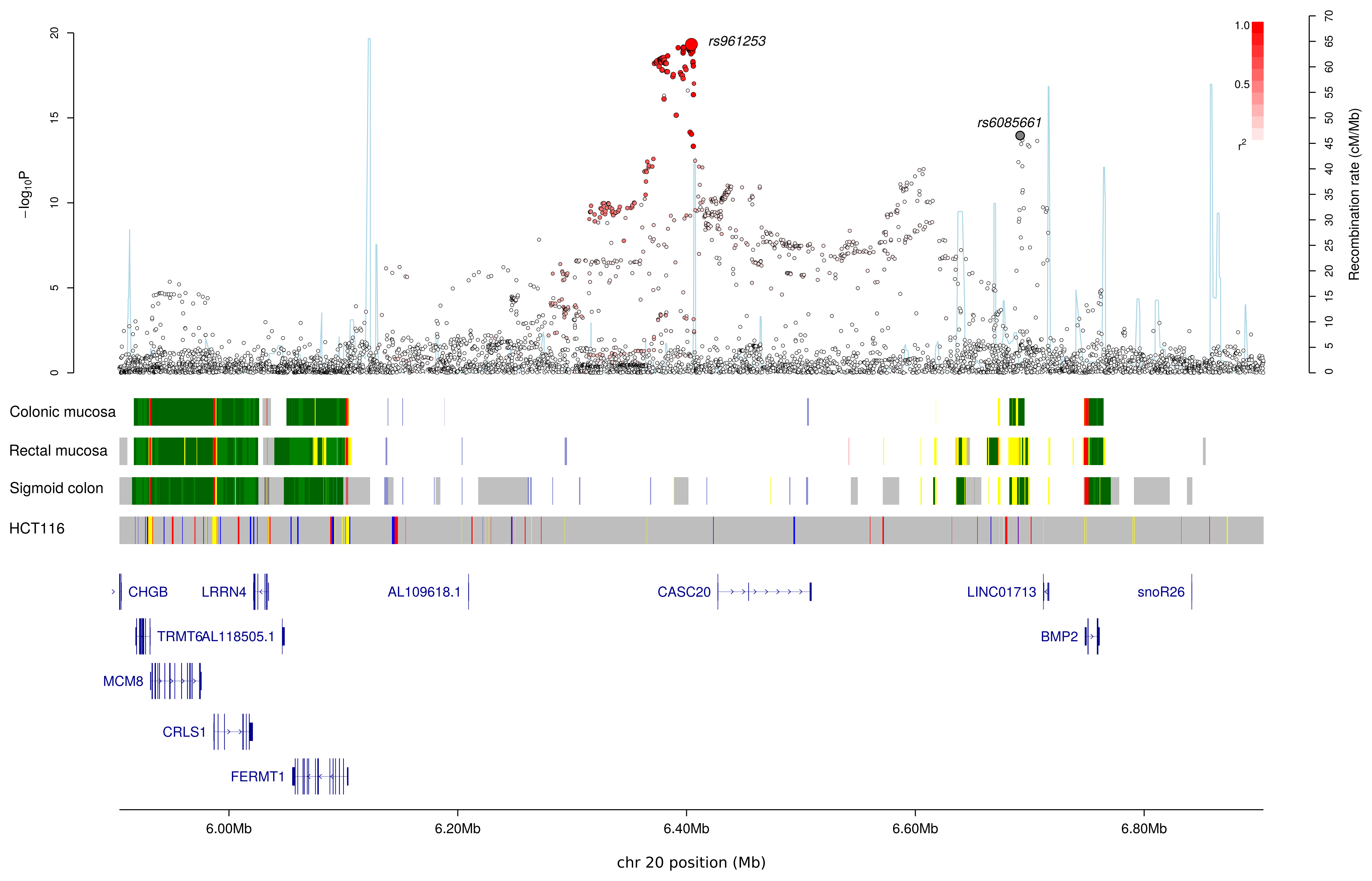

rs961253
rs6085661
1.0
0.5
r^2
$-\log_{10}P$
Recombination rate (cM/Mb)
Colonic mucosa
Rectal mucosa
Sigmoid colon
HCT116
CHGB
LRRN4
AL109618.1
CASC20
LINC01713
snoR26
TRMT6
AL118505.1
BMP2
MCM8
CRLS1
FERMT1
6.00Mb
6.20Mb
6.40Mb
6.60Mb
6.80Mb
chr 20 position (Mb)

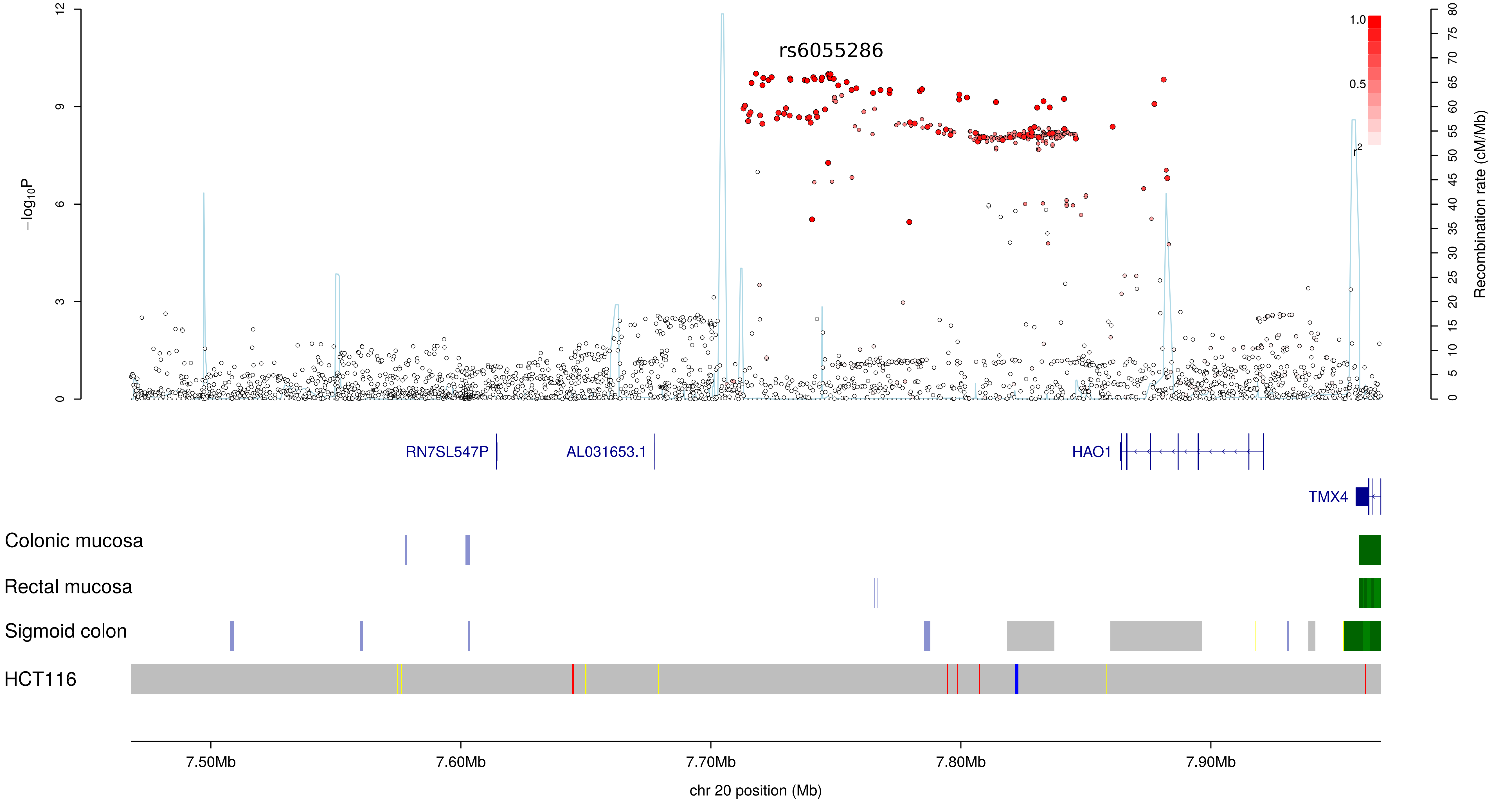

rs6055286
−log10P
Recombination rate (cM/Mb)
r2
1.0
0.5
RN7SL547P
AL031653.1
HAO1
TMX4
Colonic mucosa
Rectal mucosa
Sigmoid colon
HCT116
7.50Mb
7.60Mb
7.70Mb
7.80Mb
7.90Mb
chr 20 position (Mb)

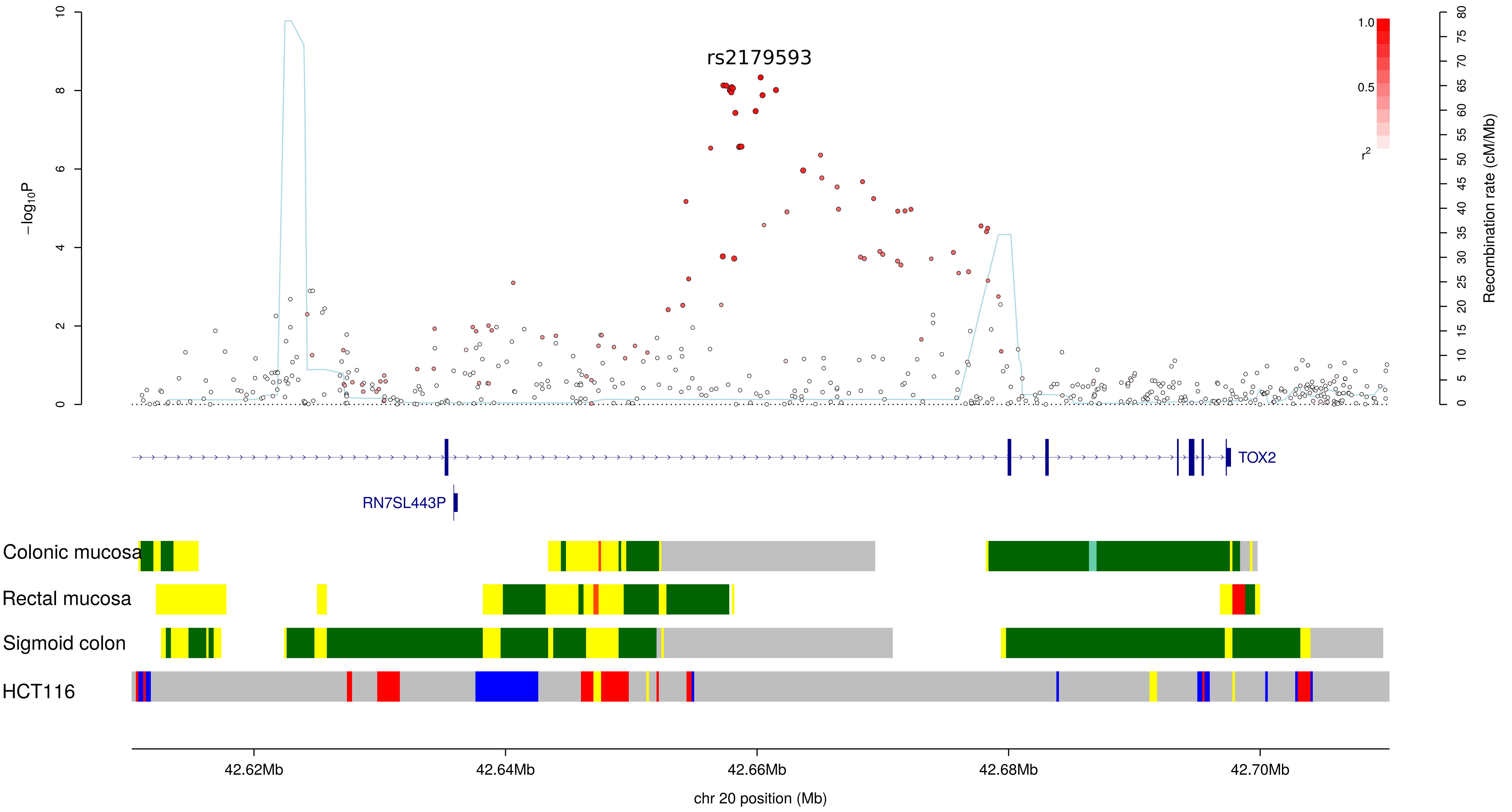
rs2179593
−log10P
Recombination rate (cM/Mb)
r2
1.0
0.5
TOX2
RN7SL443P
Colonic mucosa
Rectal mucosa
Sigmoid colon
HCT116
42.62Mb
42.64Mb
42.66Mb
42.68Mb
42.70Mb
chr 20 position (Mb)

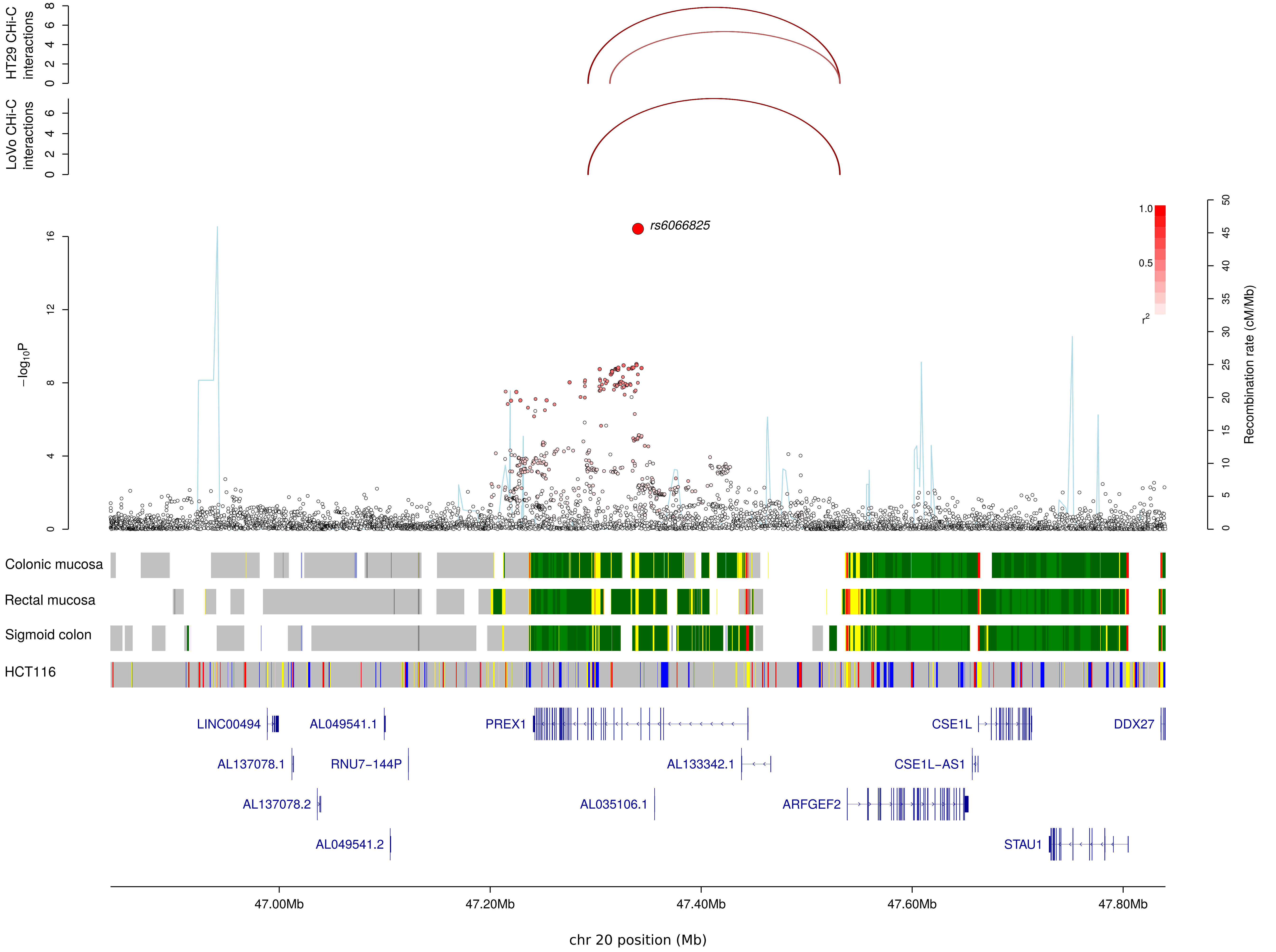

HT29 CHi-C interactions
LoVo CHi-C interactions
rs6066825
1.0
0.5
r^2
$-\log_{10}P$
Recombination rate (cM/Mb)
Colonic mucosa
Rectal mucosa
Sigmoid colon
HCT116
LINC00494
AL049541.1
PREX1
CSE1L
DDX27
AL137078.1
RNU7−144P
AL133342.1
CSE1L−AS1
AL137078.2
AL035106.1
ARFGEF2
AL049541.2
STAU1
47.00Mb
47.20Mb
47.40Mb
47.60Mb
47.80Mb
chr 20 position (Mb)

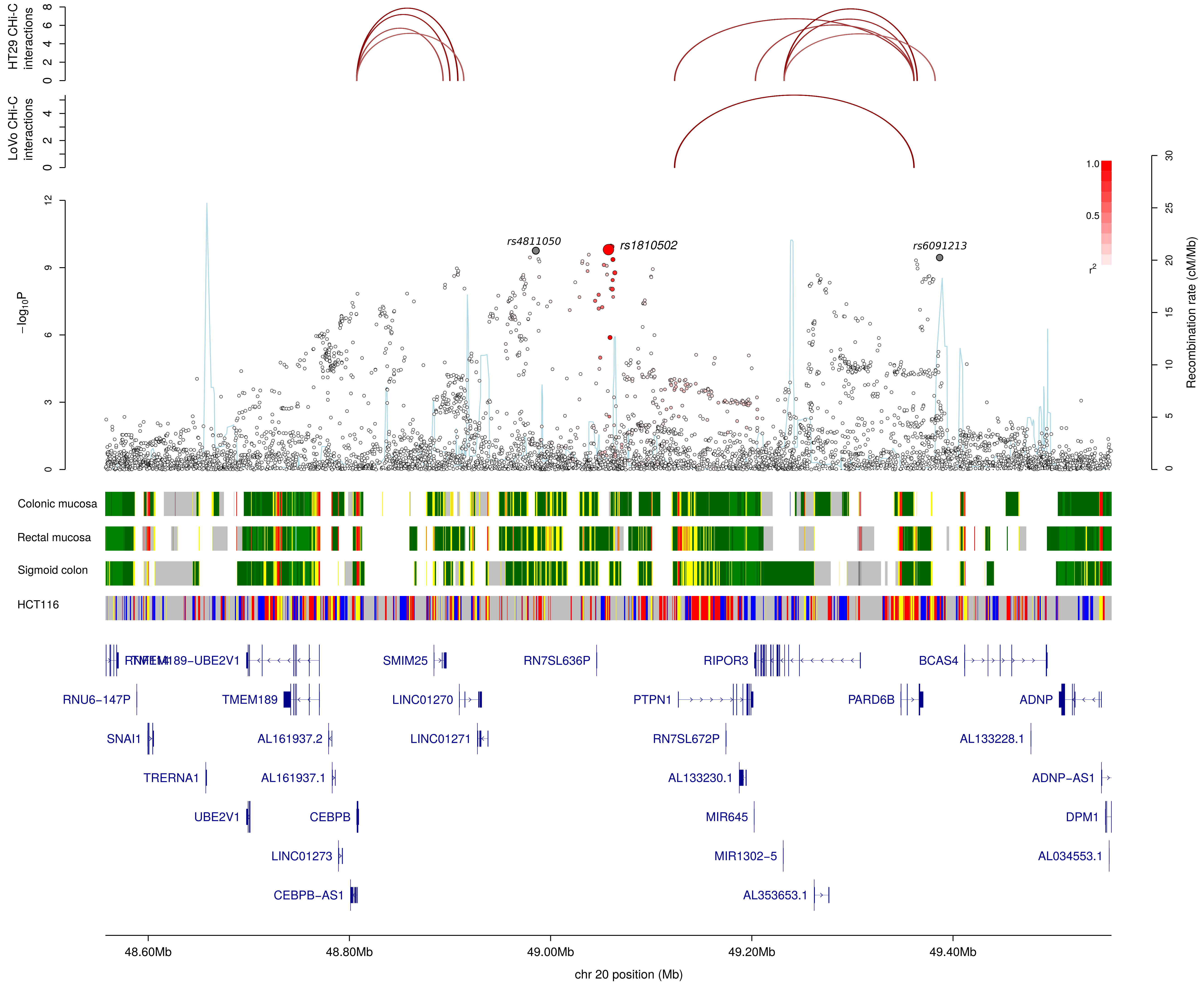

HT29 CHi-C interactions
LoVo CHi-C interactions
−log10P
Recombination rate (cM/Mb)
rs4811050
rs1810502
rs6091213
r2
Colonic mucosa
Rectal mucosa
Sigmoid colon
HCT116
RNF114
TMEM189−UBE2V1
SMIM25
RN7SL636P
RIPOR3
BCAS4
RNU6−147P
TMEM189
LINC01270
PTPN1
PARD6B
ADNP
SNAI1
AL161937.2
LINC01271
RN7SL672P
AL133228.1
TRERNA1
AL161937.1
AL133230.1
ADNP−AS1
UBE2V1
CEBPB
MIR645
DPM1
LINC01273
MIR1302−5
AL034553.1
CEBPB−AS1
AL353653.1
48.60Mb
48.80Mb
49.00Mb
49.20Mb
49.40Mb
chr 20 position (Mb)

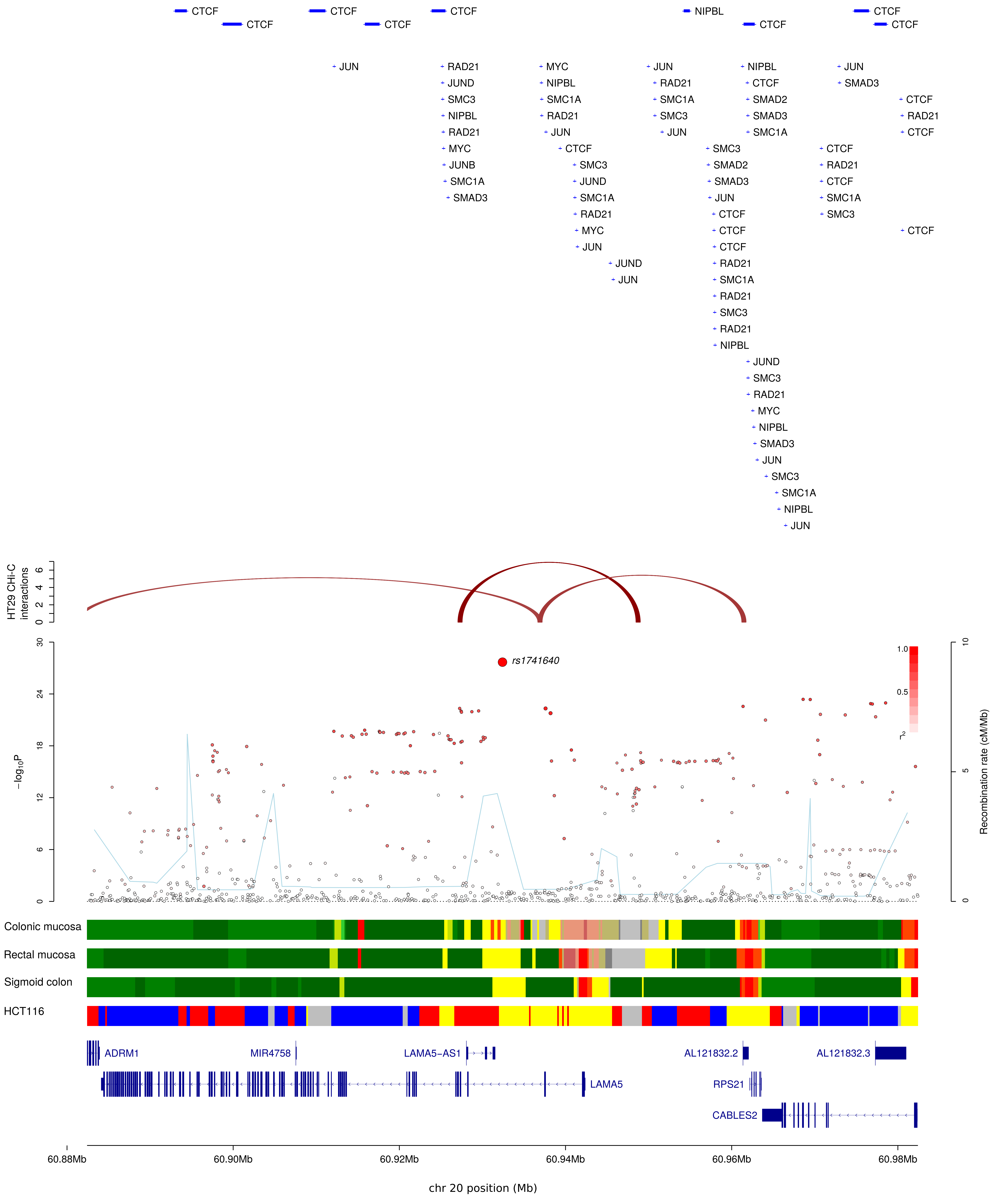
CTCF
CTCF
CTCF
CTCF
CTCF
NIPBL
CTCF
CTCF
CTCF
JUN
RAD21
JUND
SMC3
NIPBL
RAD21
MYC
JUNB
SMC1A
SMAD3
MYC
NIPBL
SMC1A
RAD21
JUN
CTCF
SMC3
JUND
SMC1A
RAD21
MYC
JUN
JUND
JUN
JUN
RAD21
SMC1A
SMC3
JUN
NIPBL
CTCF
SMAD2
SMAD3
SMC1A
SMC3
SMAD2
SMAD3
JUN
CTCF
CTCF
CTCF
RAD21
SMC1A
RAD21
SMC3
RAD21
NIPBL
JUND
SMC3
RAD21
MYC
NIPBL
SMAD3
JUN
SMC3
SMC1A
NIPBL
JUN
JUN
SMAD3
CTCF
RAD21
CTCF
RAD21
CTCF
SMC1A
SMC3
CTCF
CTCF
RAD21
CTCF
CTCF
HT29 CHi-C interactions
0
2
4
6
rs1741640
$-\log_{10}P$
0
6
12
18
24
30
1.0
0.5
r^2
Recombination rate (cM/Mb)
0
5
10
Colonic mucosa
Rectal mucosa
Sigmoid colon
HCT116
ADRM1
MIR4758
LAMA5-AS1
AL121832.2
AL121832.3
LAMA5
RPS21
CABLES2
60.88Mb
60.90Mb
60.92Mb
60.94Mb
60.96Mb
60.98Mb
chr 20 position (Mb)

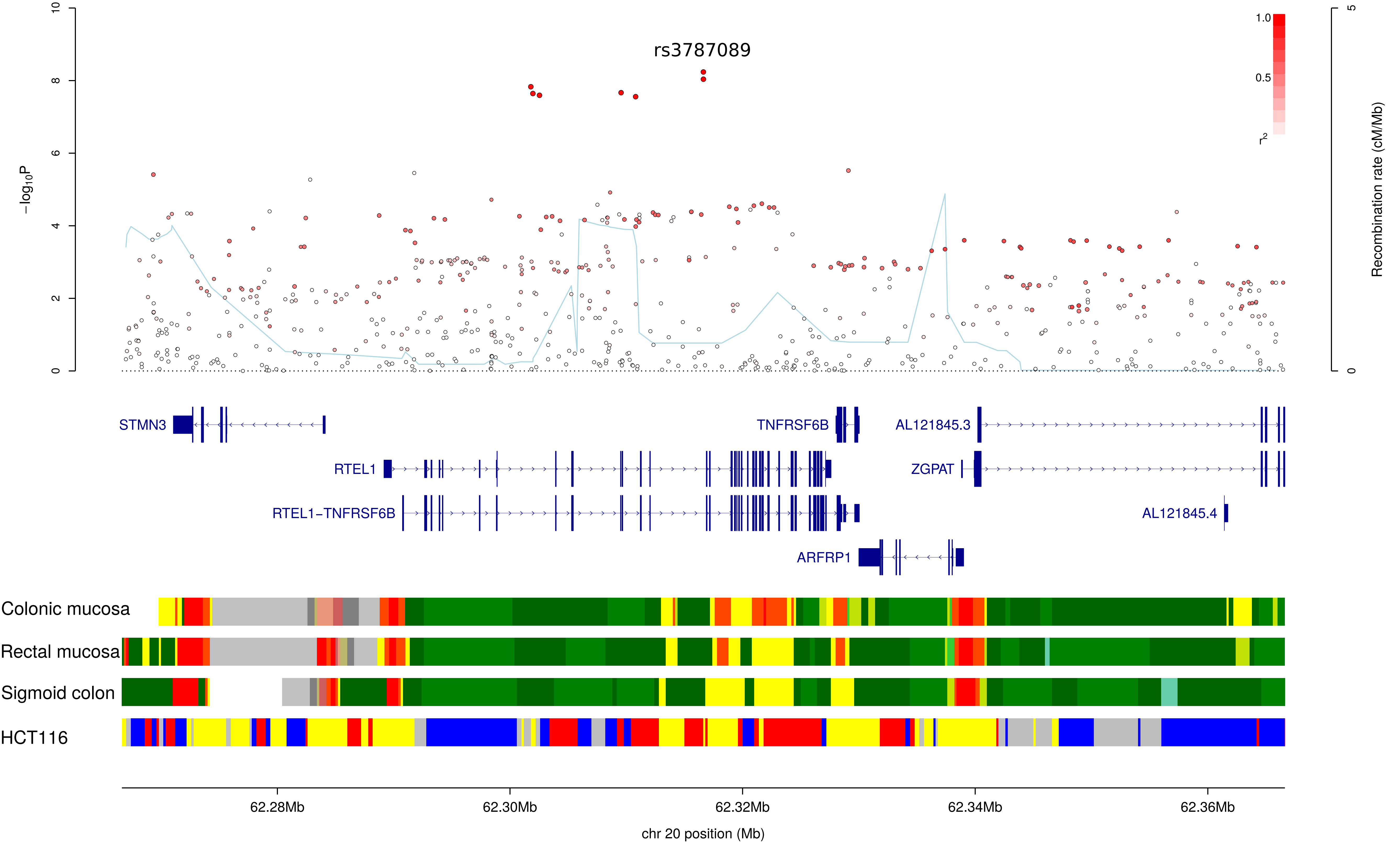

rs3787089
−log10P
Recombination rate (cM/Mb)
1.0
0.5
r2
STMN3
TNFRSF6B
AL121845.3
RTEL1
ZGPAT
RTEL1−TNFRSF6B
AL121845.4
ARFRP1
Colonic mucosa
Rectal mucosa
Sigmoid colon
HCT116
62.28Mb
62.30Mb
62.32Mb
62.34Mb
62.36Mb
chr 20 position (Mb)

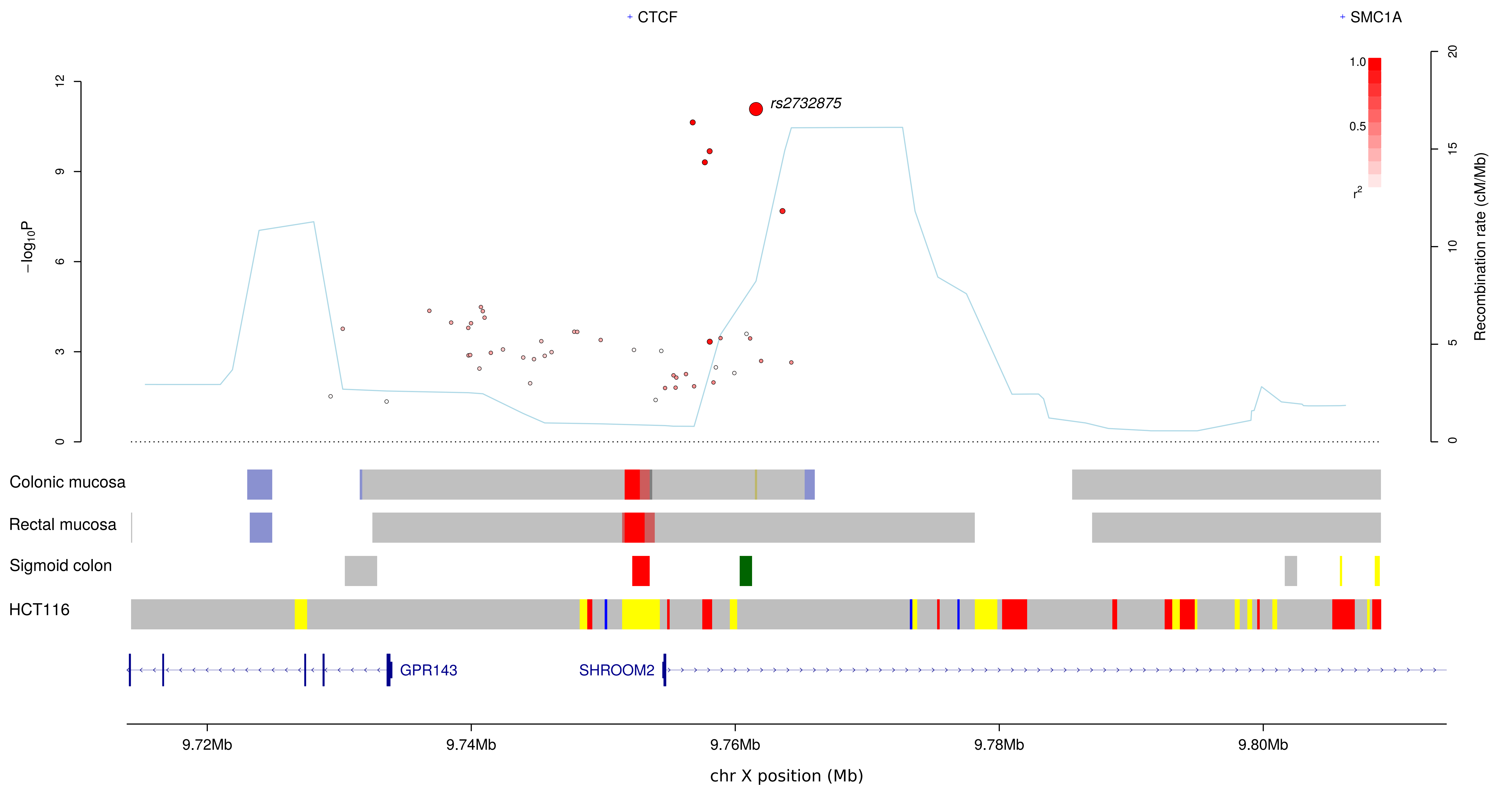
CTCF
SMC1A
rs2732875
1.0
0.5
r^2
$-\log_{10}P$
0
3
6
9
12
Recombination rate (cM/Mb)
0
5
10
15
20
Colonic mucosa
Rectal mucosa
Sigmoid colon
HCT116
GPR143
SHROOM2
9.72Mb
9.74Mb
9.76Mb
9.78Mb
9.80Mb
chr X position (Mb)

Supplementary Figure 3: Heatmap of enrichment of histone marks at colorectal cancer risk locus across (a) different colonic tissues, and (b) multiple cell types. SNP score calculated using EPIGWAS.

(a)

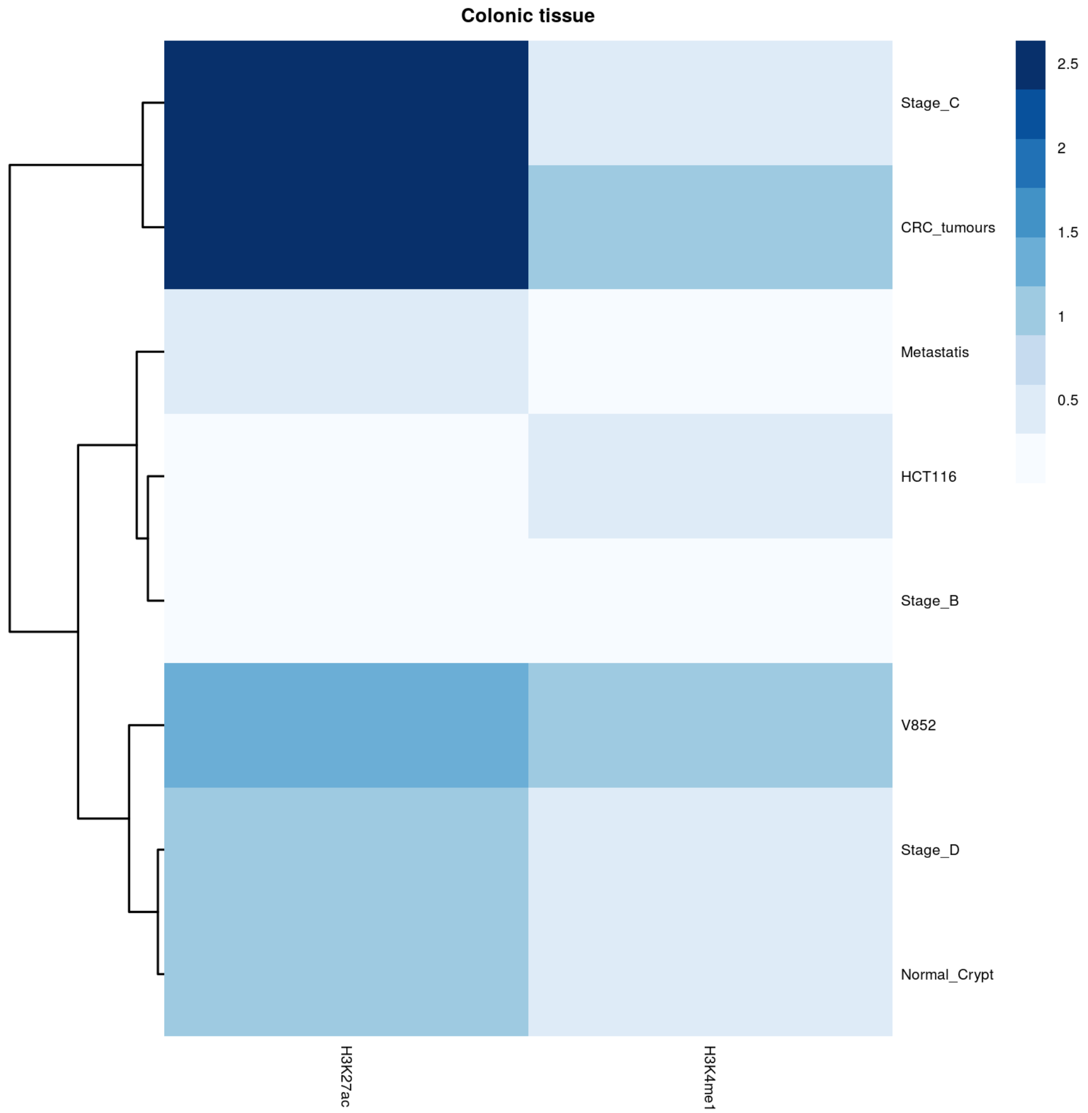

(b)

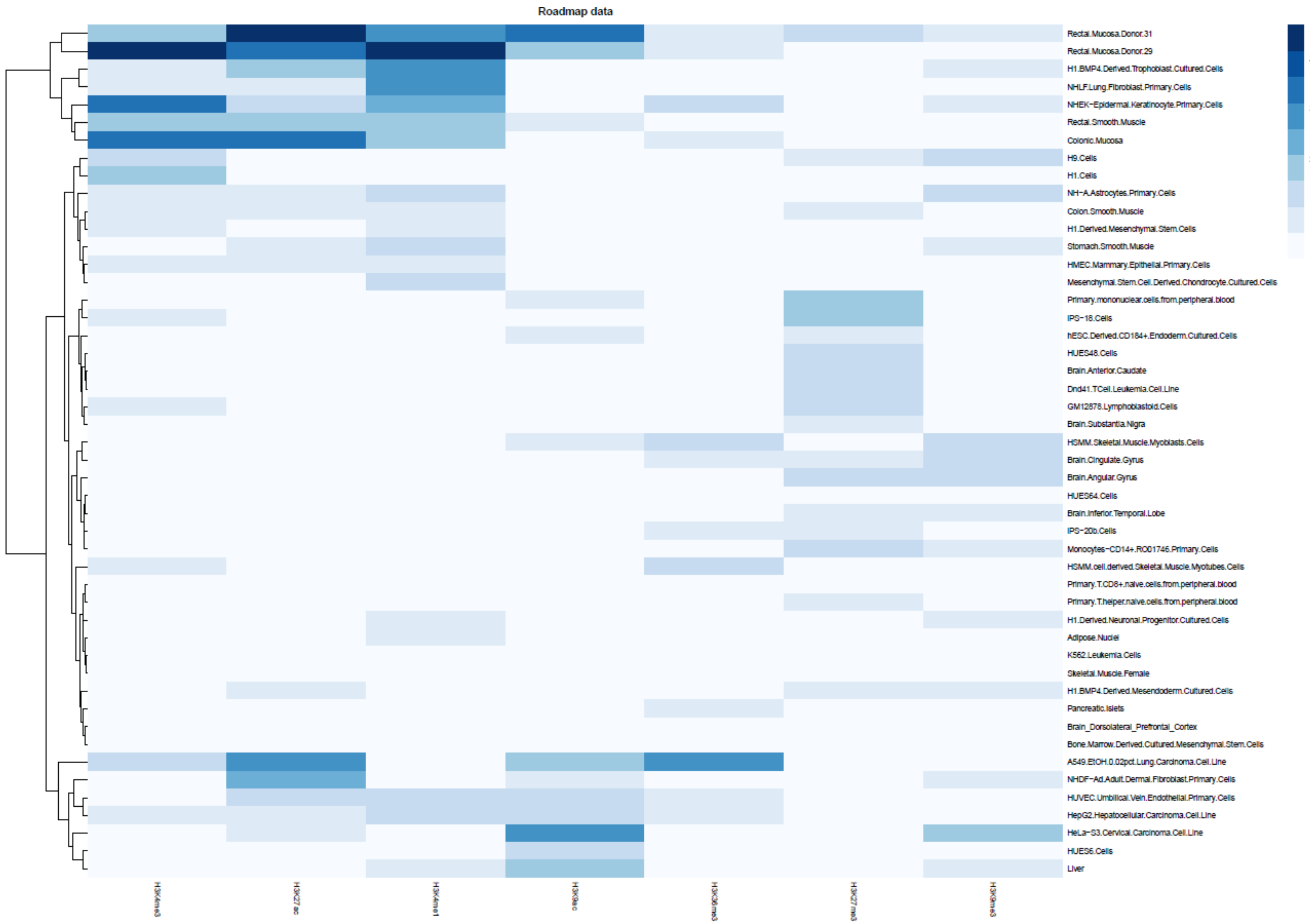
Roadmap data
Rectal.Mucosa.Donor.31
Rectal.Mucosa.Donor.29
H1.BMP4.Derived.Trophoblast.Cultured.Cells
NHLF.Lung.Fibroblast.Primary.Cells
NHEK−Epidermal.Keratinocyte.Primary.Cells
Rectal.Smooth.Muscle
Colonic.Mucosa
H9.Cells
H1.Cells
NH−A.Astrocytes.Primary.Cells
Colon.Smooth.Muscle
H1.Derived.Mesenchymal.Stem.Cells
Stomach.Smooth.Muscle
HMEC.Mammary.Epithelial.Primary.Cells
Mesenchymal.Stem.Cell.Derived.Chondrocyte.Cultured.Cells
Primary.mononuclear.cells.from.peripheral.blood
iPS−18.Cells
hESC.Derived.CD184+.Endoderm.Cultured.Cells
HUES48.Cells
Brain.Anterior.Caudate
Dnd41.TCell.Leukemia.Cell.Line
GM12878.Lymphoblastoid.Cells
Brain.Substantia.Nigra
HSMM.Skeletal.Muscle.Myoblasts.Cells
Brain.Cingulate.Gyrus
Brain.Angular.Gyrus
HUES64.Cells
Brain.Inferior.Temporal.Lobe
iPS−20b.Cells
Monocytes−CD14+.RO01746.Primary.Cells
HSMM.cell.derived.Skeletal.Muscle.Myotubes.Cells
Primary.T.CD8+.naive.cells.from.peripheral.blood
Primary.T.helper.naive.cells.from.peripheral.blood
H1.Derived.Neuronal.Progenitor.Cultured.Cells
Adipose.Nuclei
K562.Leukemia.Cells
Skeletal.Muscle.Female
H1.BMP4.Derived.Mesendoderm.Cultured.Cells
Pancreatic.Islets
Brain_Dorsolateral_Prefrontal_Cortex
Bone.Marrow.Derived.Cultured.Mesenchymal.Stem.Cells
A549.EtOH.0.02pct.Lung.Carcinoma.Cell.Line
NHDF−Ad.Adult.Dermal.Fibroblast.Primary.Cells
HUVEC.Umbilical.Vein.Endothelial.Primary.Cells
HepG2.Hepatocellular.Carcinoma.Cell.Line
HeLa−S3.Cervical.Carcinoma.Cell.Line
HUES6.Cells
Liver
H3K4me3
H3K27ac
H3K4me1
H3K9ac
H3K36me3
H3K27me3
H3K9me3

Supplementary Figure 4: Enrichment of transcription factors and histone marks in (a) LoVo and (b) HT29 cells at risk SNPs demonstrates that risk SNPs. The red line denotes the FDR *Q*-value of 0.05.

(a)

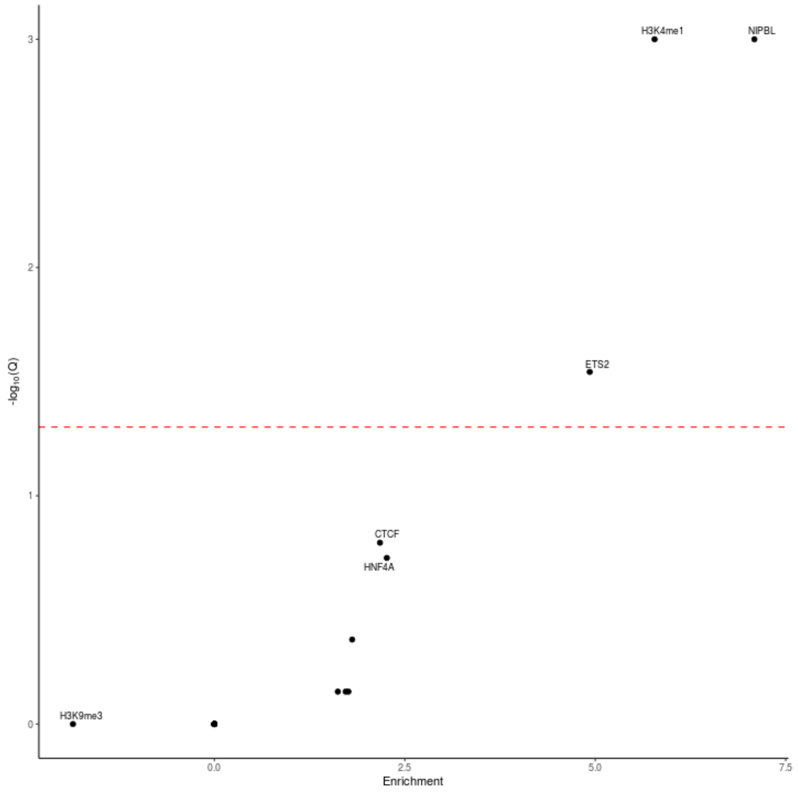

(b)

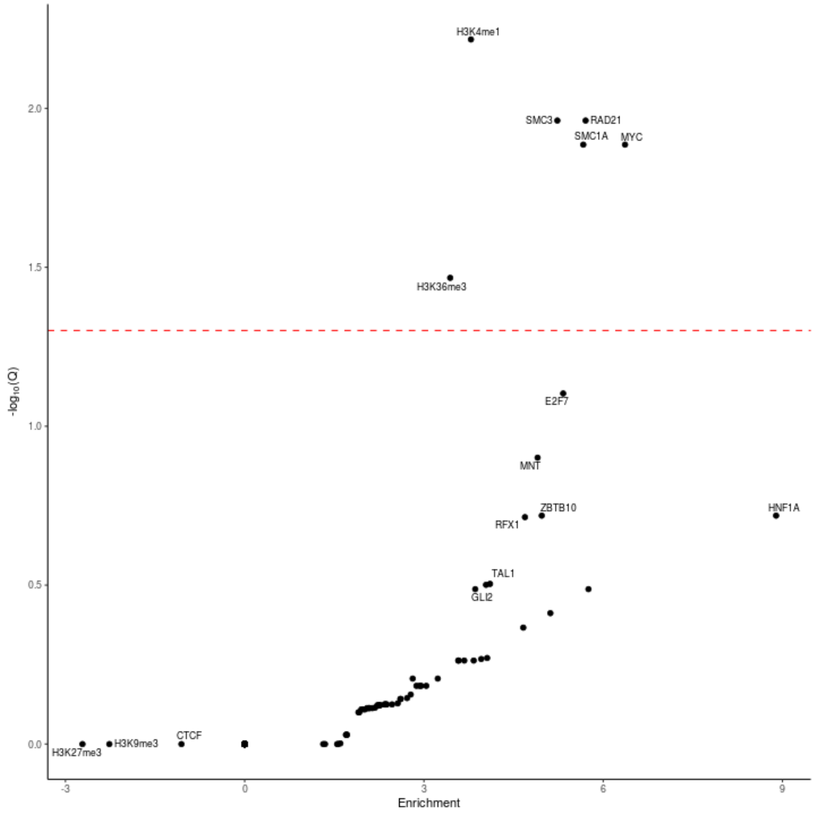

Supplementary Figure 5: Protein-protein interaction networks for colorectal cancer risk loci. Risk variants were mapped to candidate target genes, and interactions between the gene products were identified. Nodes represent credible gene products and edges represent protein-protein interactions. Nodes are coloured based on their community, i.e. genes that clustered together.

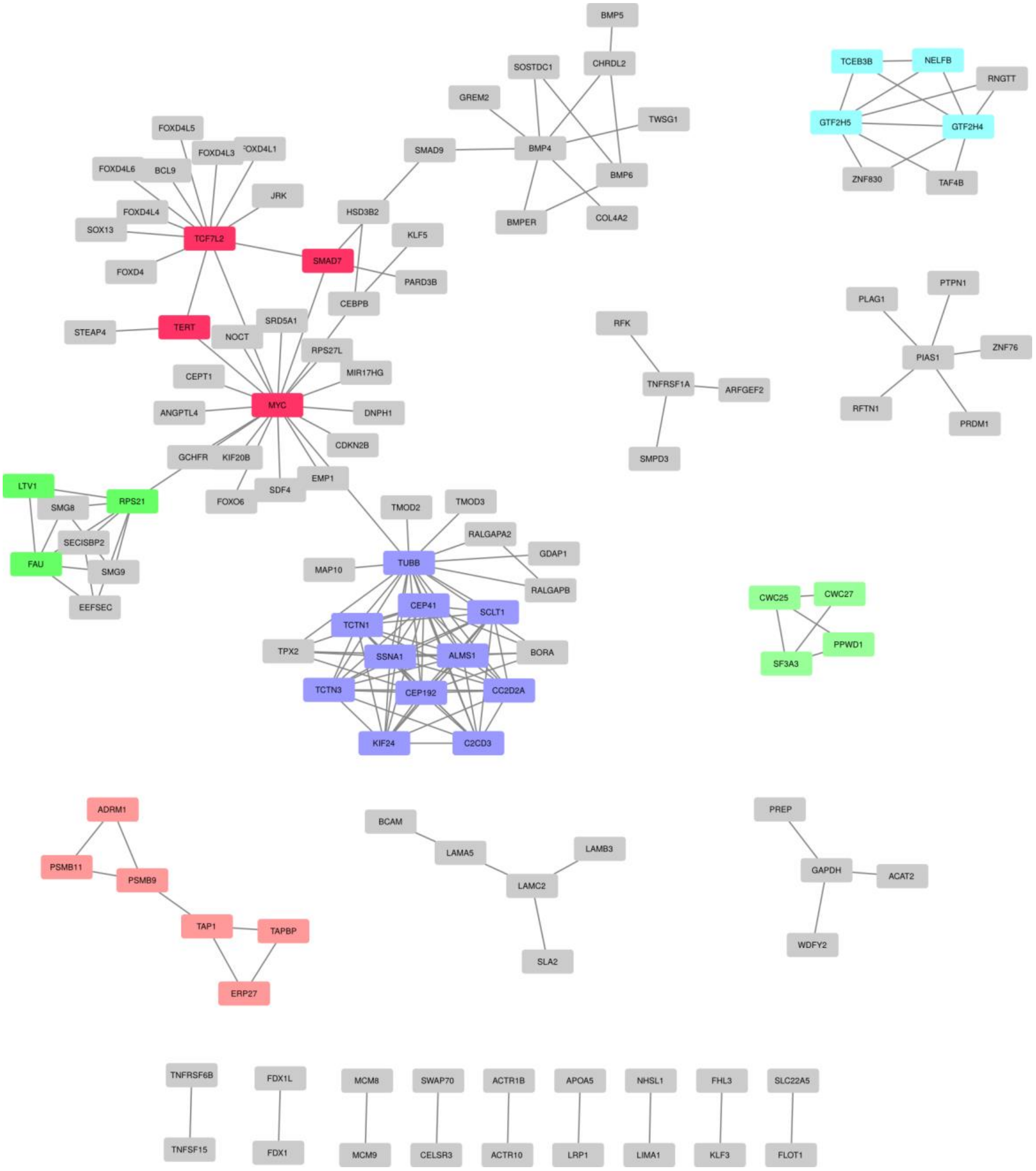

Supplementary Figure 6: Pathway enrichment using summary association statistics. Nodes represent pathways (sets of genes) and edges represent overlaps of genes within the pathways (correlation coefficient). Node size is representative of the number of genes in the pathway, and node colour represents the FDR *Q*-value from the enrichment analysis (deeper red indicates more significant). Only pathways with Q < 0.05 and connections with correlation coefficient > 0.55 are shown.

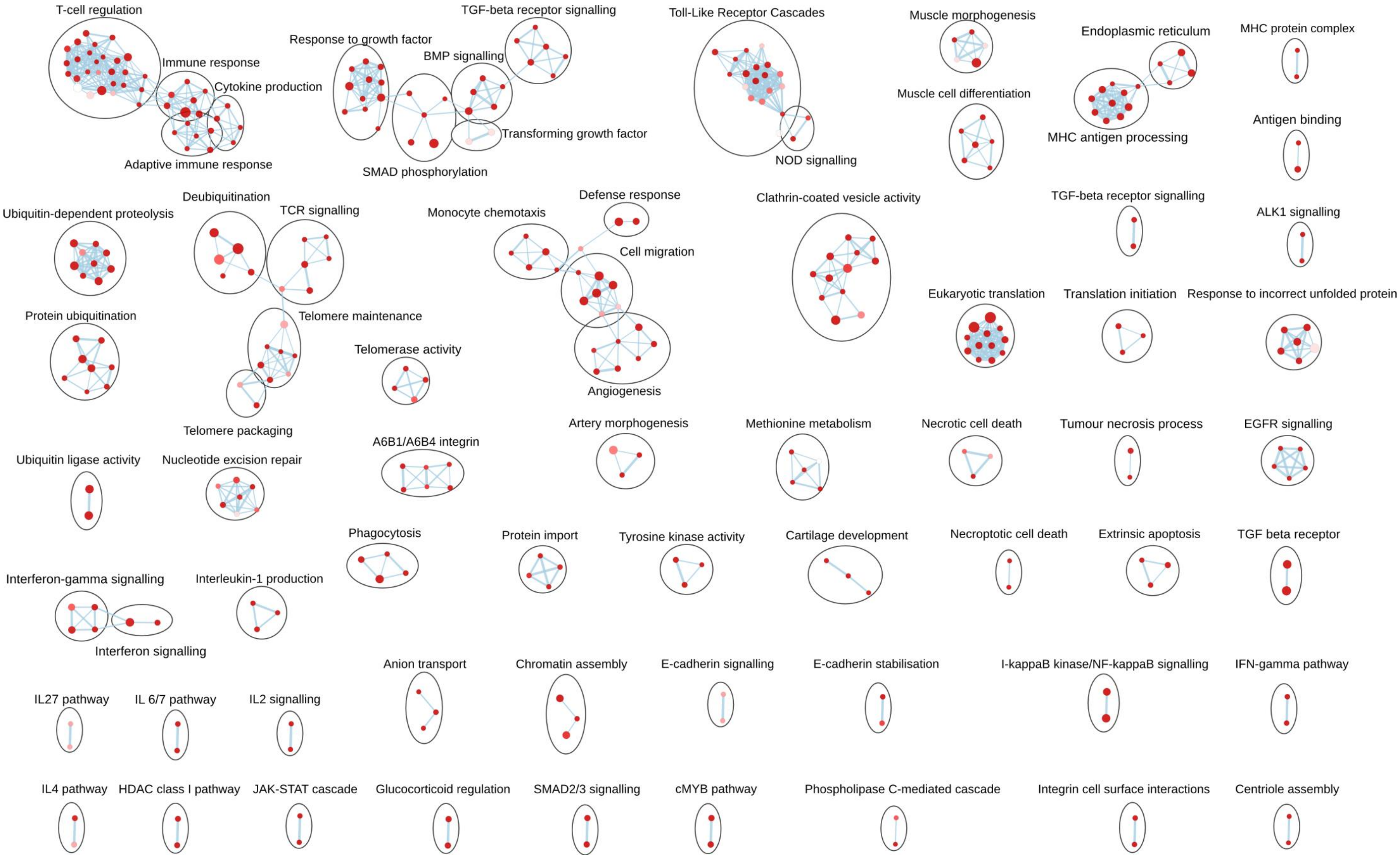

Supplementary Figure 7: Projected percentage of GWAS heritability explained for a given sample size. Results were obtained using a three-component model to estimate distribution of effect sizes. Grey shaded area represents the 95% confidence interval of the heritability estimate. The sample size indicates the total number of cases and controls, assuming a 1:1 ratio.

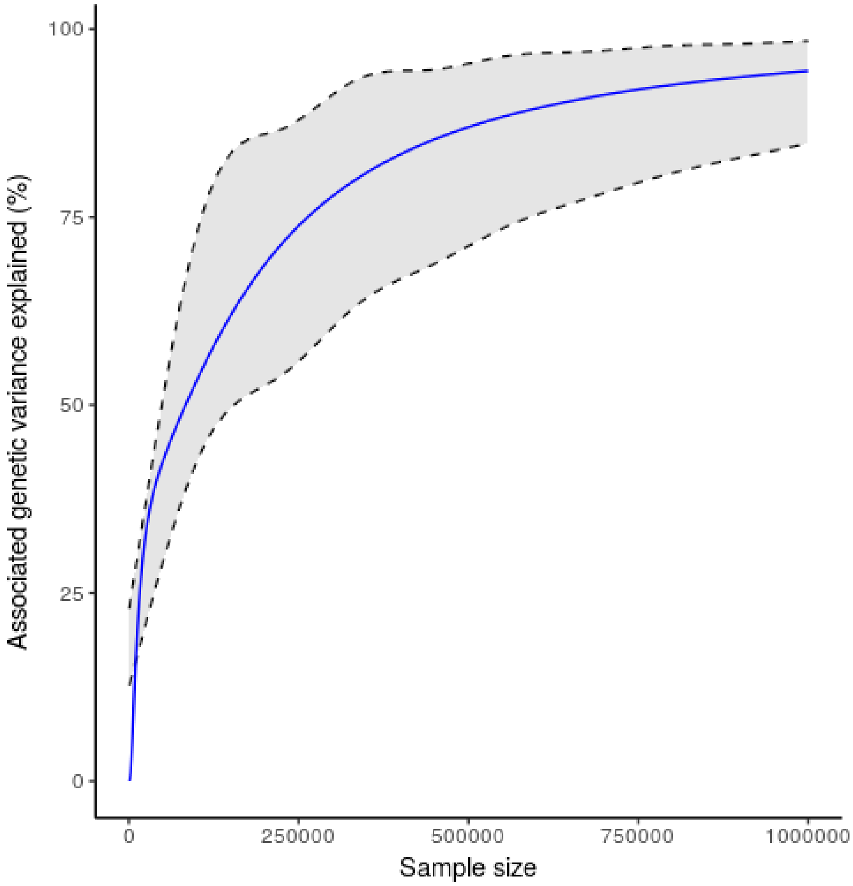

Supplementary Figure 8: Population risk distribution ordered by relative risk (RR), compared to the population average. Vertical dotted lines (left to right) correspond to 1%, 10%, 50%, 90%, and 99% centile, respectively. The RR distribution in the top figure was based on the 79 identified CRC risk SNPs, and the RR distribution in the bottom figure is based on assuming all common variation.

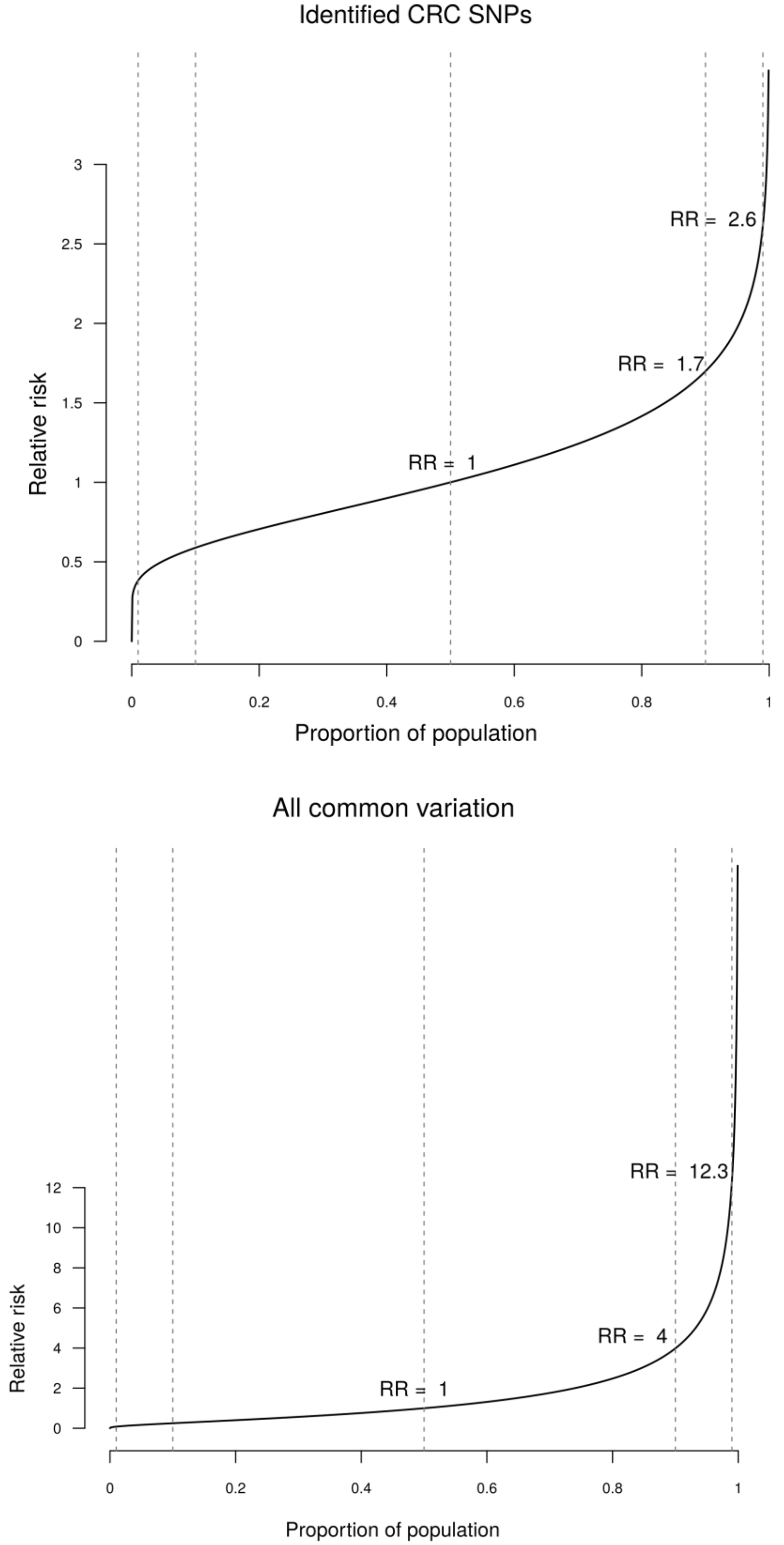

Supplementary Figure 9: Identification of individuals of non-European ancestry in cases and controls in the new GWAS. (a) NSCCG-OncoArray, (b) SCOT, (c) SOCCS/GS, (d) SOCCS/LBC, (e) UK Biobank. The first two principal components of the analysis are plotted. HapMap CEU individuals are plotted in red, JPT individuals are plotted in indigo, YRI are plotted in green. Cases and controls are plotted in black.

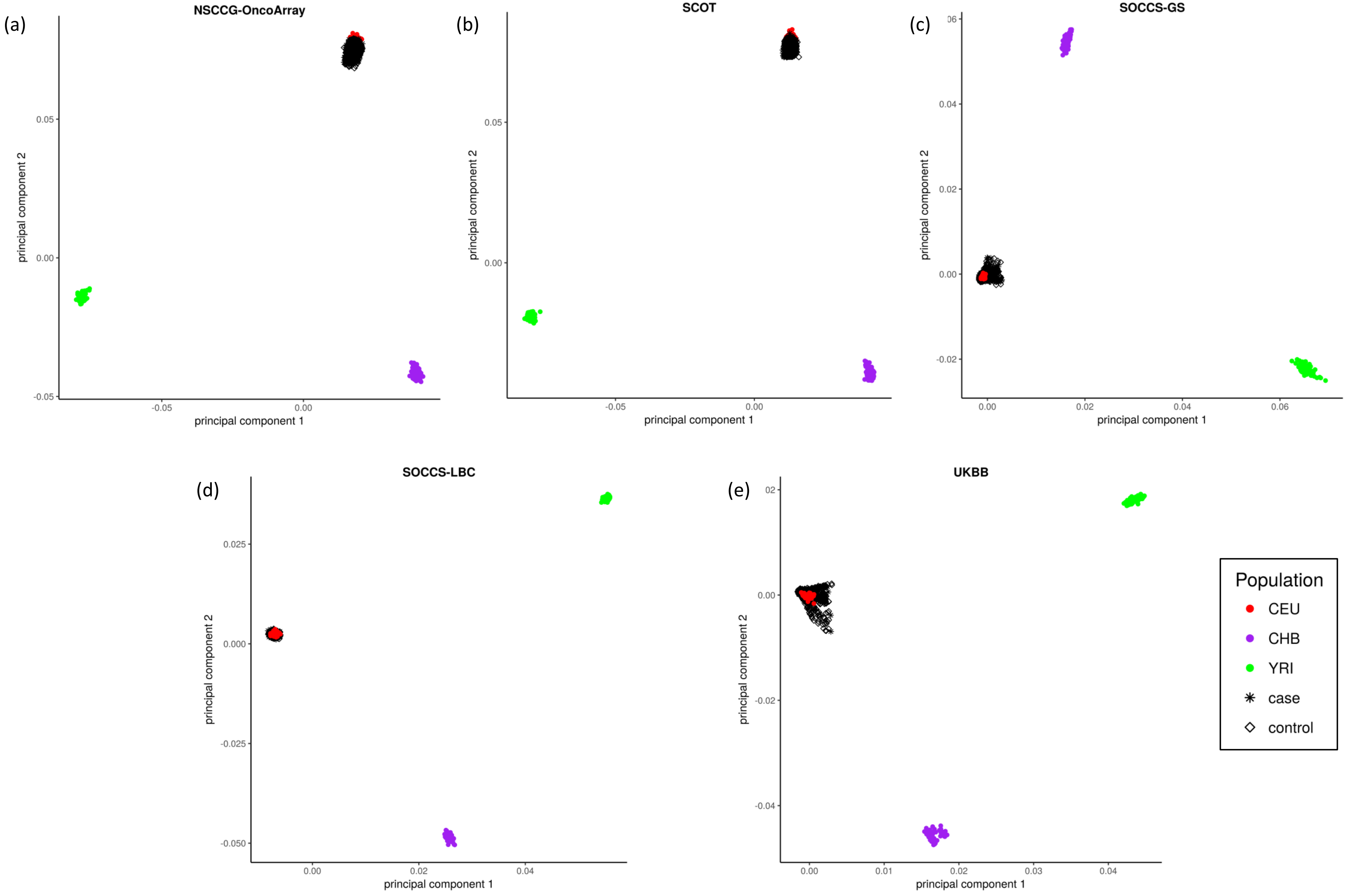

SUPPLEMENTARY REFERENCES

1. Al-Tassan, N.A. *et al.* A new GWAS and meta-analysis with 1000Genomes imputation identifies novel risk variants for colorectal cancer. *Sci Rep* **5**, 10442 (2015).
2. Broderick, P. *et al.* A genome-wide association study shows that common alleles of SMAD7 influence colorectal cancer risk. *Nat Genet* **39**, 1315-7 (2007).
3. Dunlop, M.G. *et al.* Common variation near CDKN1A, POLD3 and SHROOM2 influences colorectal cancer risk. *Nat Genet* **44**, 770-6 (2012).
4. Houlston, R.S. *et al.* Meta-analysis of three genome-wide association studies identifies susceptibility loci for colorectal cancer at 1q41, 3q26.2, 12q13.13 and 20q13.33. *Nat Genet* **42**, 973-7 (2010).
5. Orlando, G. *et al.* Variation at 2q35 (PNKD and TMBIM1) influences colorectal cancer risk and identifies a pleiotropic effect with inflammatory bowel disease. *Hum Mol Genet* **25**, 2349-2359 (2016).
6. Peters, U. *et al.* Identification of Genetic Susceptibility Loci for Colorectal Tumors in a Genome-Wide Meta-analysis. *Gastroenterology* **144**, 799-807 e24 (2013).
7. Real, L.M. *et al.* A colorectal cancer susceptibility new variant at 4q26 in the Spanish population identified by genome-wide association analysis. *PLoS One* **9**, e101178 (2014).
8. Schmit, S.L. *et al.* Novel Common Genetic Susceptibility Loci for Colorectal Cancer. *J Natl Cancer Inst* (2018).
9. Schmit, S.L. *et al.* A novel colorectal cancer risk locus at 4q32.2 identified from an international genome-wide association study. *Carcinogenesis* **35**, 2512-9 (2014).
10. Schumacher, F.R. *et al.* Genome-wide association study of colorectal cancer identifies six new susceptibility loci. *Nat Commun* **6**, 7138 (2015).
11. Cogent Study *et al.* Meta-analysis of genome-wide association data identifies four new susceptibility loci for colorectal cancer. *Nat Genet* **40**, 1426-35 (2008).
12. Tenesa, A. *et al.* Genome-wide association scan identifies a colorectal cancer susceptibility locus on 11q23 and replicates risk loci at 8q24 and 18q21. *Nat Genet* **40**, 631-7 (2008).
13. Tomlinson, I. *et al.* A genome-wide association scan of tag SNPs identifies a susceptibility variant for colorectal cancer at 8q24.21. *Nat Genet* **39**, 984-8 (2007).
14. Tomlinson, I.P. *et al.* Multiple common susceptibility variants near BMP pathway loci GREM1, BMP4, and BMP2 explain part of the missing heritability of colorectal cancer. *PLoS Genet* **7**, e1002105 (2011).
15. Tomlinson, I.P. *et al.* A genome-wide association study identifies colorectal cancer susceptibility loci on chromosomes 10p14 and 8q23.3. *Nat Genet* **40**, 623-30 (2008).
16. Whiffin, N. *et al.* Identification of susceptibility loci for colorectal cancer in a genome-wide meta-analysis. *Hum Mol Genet* **23**, 4729-37 (2014).
17. Jia, W.H. *et al.* Genome-wide association analyses in East Asians identify new susceptibility loci for colorectal cancer. *Nat Genet* **45**, 191-6 (2013).
18. Jiang, K. *et al.* Genome-wide association study identifies two new susceptibility loci for colorectal cancer at 5q23.3 and 17q12 in Han Chinese. *Oncotarget* **6**, 40327-36 (2015).
19. Tanikawa, C. *et al.* GWAS identifies two novel colorectal cancer loci at 16q24.1 and 20q13.12. *Carcinogenesis* **39**, 652-660 (2018).
20. Wang, H. *et al.* Trans-ethnic genome-wide association study of colorectal cancer identifies a new susceptibility locus in VTI1A. *Nat Commun* **5**, 4613 (2014).
21. Wang, M. *et al.* Common genetic variation in ETV6 is associated with colorectal cancer susceptibility. *Nat Commun* **7**, 11478 (2016).

22. Zeng, C. *et al.* Identification of Susceptibility Loci and Genes for Colorectal Cancer Risk. *Gastroenterology* **150**, 1633-1645 (2016).
23. Zhang, B. *et al.* Large-scale genetic study in East Asians identifies six new loci associated with colorectal cancer risk. *Nat Genet* **46**, 533-42 (2014).
24. Robinson, M.D. & Oshlack, A. A scaling normalization method for differential expression analysis of RNA-seq data. *Genome Biol* **11**, R25 (2010).
25. Ongen, H., Buil, A., Brown, A.A., Dermitzakis, E.T. & Delaneau, O. Fast and efficient QTL mapper for thousands of molecular phenotypes. *Bioinformatics* **32**, 1479-85 (2016).
26. Stegle, O., Parts, L., Piipari, M., Winn, J. & Durbin, R. Using probabilistic estimation of expression residuals (PEER) to obtain increased power and interpretability of gene expression analyses. *Nat Protoc* **7**, 500-7 (2012).
27. Storey, J.D. & Tibshirani, R. Statistical significance for genomewide studies. *Proc Natl Acad Sci U S A* **100**, 9440-5 (2003).
28. Sul, J.H., Han, B., Ye, C., Choi, T. & Eskin, E. Effectively identifying eQTLs from multiple tissues by combining mixed model and meta-analytic approaches. *PLoS Genet* **9**, e1003491 (2013).
29. Langmead, B. & Salzberg, S.L. Fast gapped-read alignment with Bowtie 2. *Nat Methods* **9**, 357-9 (2012).
30. Wingett, S. *et al.* HiCUP: pipeline for mapping and processing Hi-C data. *F1000Res* **4**, 1310 (2015).
31. Lohse, M. *et al.* RobiNA: a user-friendly, integrated software solution for RNA-Seq-based transcriptomics. *Nucleic Acids Res* **40**, W622-7 (2012).
32. Feng, J., Liu, T., Qin, B., Zhang, Y. & Liu, X.S. Identifying ChIP-seq enrichment using MACS. *Nat Protoc* **7**, 1728-40 (2012).
33. Ward, L.D. & Kellis, M. HaploReg v4: systematic mining of putative causal variants, cell types, regulators and target genes for human complex traits and disease. *Nucleic Acids Res* **44**, D877-81 (2016).
34. Kheradpour, P. & Kellis, M. Systematic discovery and characterization of regulatory motifs in ENCODE TF binding experiments. *Nucleic Acids Res* **42**, 2976-87 (2014).
35. Wang, J. *et al.* Sequence features and chromatin structure around the genomic regions bound by 119 human transcription factors. *Genome Res* **22**, 1798-812 (2012).
36. Kulakovskiy, I.V. *et al.* HOCOMOCO: a comprehensive collection of human transcription factor binding sites models. *Nucleic Acids Res* **41**, D195-202 (2013).
37. Heinz, S. *et al.* Simple combinations of lineage-determining transcription factors prime cis-regulatory elements required for macrophage and B cell identities. *Mol Cell* **38**, 576-89 (2010).
38. Zhu, Z. *et al.* A genome-wide cross-trait analysis from UK Biobank highlights the shared genetic architecture of asthma and allergic diseases. *Nat Genet* **50**, 857-864 (2018).
39. Nelson, C.P. *et al.* Association analyses based on false discovery rate implicate new loci for coronary artery disease. *Nat Genet* **49**, 1385-1391 (2017).
40. de Oliveira Otto, M.C. *et al.* Genome-wide association meta-analysis of circulating odd-numbered chain saturated fatty acids: Results from the CHARGE Consortium. *PLoS One* **13**, e0196951 (2018).
41. Guan, W. *et al.* Genome-wide association study of plasma N6 polyunsaturated fatty acids within the cohorts for heart and aging research in genomic epidemiology consortium. *Circ Cardiovasc Genet* **7**, 321-331 (2014).
42. Lemaitre, R.N. *et al.* Genetic loci associated with circulating levels of very long-chain saturated fatty acids. *J Lipid Res* **56**, 176-84 (2015).

43. Lemaitre, R.N. *et al.* Genetic loci associated with plasma phospholipid n-3 fatty acids: a meta-analysis of genome-wide association studies from the CHARGE Consortium. *PLoS Genet* **7**, e1002193 (2011).
44. Mozaffarian, D. *et al.* Genetic loci associated with circulating phospholipid trans fatty acids: a meta-analysis of genome-wide association studies from the CHARGE Consortium. *Am J Clin Nutr* **101**, 398-406 (2015).
45. Wu, J.H. *et al.* Genome-wide association study identifies novel loci associated with concentrations of four plasma phospholipid fatty acids in the de novo lipogenesis pathway: results from the Cohorts for Heart and Aging Research in Genomic Epidemiology (CHARGE) consortium. *Circ Cardiovasc Genet* **6**, 171-83 (2013).
46. Willer, C.J. *et al.* Discovery and refinement of loci associated with lipid levels. *Nat Genet* **45**, 1274-1283 (2013).
47. de Lange, K.M. *et al.* Genome-wide association study implicates immune activation of multiple integrin genes in inflammatory bowel disease. *Nat Genet* **49**, 256-261 (2017).
48. Okada, Y. *et al.* Genetics of rheumatoid arthritis contributes to biology and drug discovery. *Nature* **506**, 376-81 (2014).
49. Paternoster, L. *et al.* Multi-ancestry genome-wide association study of 21,000 cases and 95,000 controls identifies new risk loci for atopic dermatitis. *Nat Genet* **47**, 1449-1456 (2015).
50. Dubois, P.C. *et al.* Multiple common variants for celiac disease influencing immune gene expression. *Nat Genet* **42**, 295-302 (2010).
51. Patsopoulos, N.A. *et al.* Genome-wide meta-analysis identifies novel multiple sclerosis susceptibility loci. *Ann Neurol* **70**, 897-912 (2011).
52. Cordell, H.J. *et al.* International genome-wide meta-analysis identifies new primary biliary cirrhosis risk loci and targetable pathogenic pathways. *Nat Commun* **6**, 8019 (2015).
53. Bentham, J. *et al.* Genetic association analyses implicate aberrant regulation of innate and adaptive immunity genes in the pathogenesis of systemic lupus erythematosus. *Nat Genet* **47**, 1457-1464 (2015).
54. Yengo, L. *et al.* Meta-analysis of genome-wide association studies for height and body mass index in ~700,000 individuals of European ancestry. *bioRxiv* (2018).
55. Lu, Y. *et al.* New loci for body fat percentage reveal link between adiposity and cardiometabolic disease risk. *Nat Commun* **7**, 10495 (2016).
56. Manning, A.K. *et al.* A genome-wide approach accounting for body mass index identifies genetic variants influencing fasting glycemic traits and insulin resistance. *Nat Genet* **44**, 659-69 (2012).
57. Wheeler, E. *et al.* Impact of common genetic determinants of Hemoglobin A1c on type 2 diabetes risk and diagnosis in ancestrally diverse populations: A transethnic genome-wide meta-analysis. *PLoS Med* **14**, e1002383 (2017).
58. Horikoshi, M. *et al.* Genome-wide associations for birth weight and correlations with adult disease. *Nature* **538**, 248-252 (2016).
59. van der Valk, R.J. *et al.* A novel common variant in DCST2 is associated with length in early life and height in adulthood. *Hum Mol Genet* **24**, 1155-68 (2015).
60. Bradfield, J.P. *et al.* A genome-wide association meta-analysis identifies new childhood obesity loci. *Nat Genet* **44**, 526-31 (2012).
61. Felix, J.F. *et al.* Genome-wide association analysis identifies three new susceptibility loci for childhood body mass index. *Hum Mol Genet* **25**, 389-403 (2016).
62. Gorski, M. *et al.* 1000 Genomes-based meta-analysis identifies 10 novel loci for kidney function. *Sci Rep* **7**, 45040 (2017).
63. Scott, R.A. *et al.* An Expanded Genome-Wide Association Study of Type 2 Diabetes in Europeans. *Diabetes* **66**, 2888-2902 (2017).
