# Supplementary material for: Association analyses identify 31 new risk loci for colorectal cancer susceptibility
Source: Nat Commun. 2019 May 14;10:2154. doi: 10.1038/s41467-019-09775-w (PMC6517433; doi:10.1038/s41467-019-09775-w)
Supplement: Supplementary file 3 — Description of Additional Supplementary Files [file 41467_2019_9775_MOESM3_ESM.pdf]

Description of Additional Supplementary Information

File Name: Supplementary Data 1
Description: Previously identified colorectal cancer risk SNPs. The previously reported association statistics in European (EUR), Asian (ASN), and African cohorts (and where performed, combined analysis) are shown. Also shown in the row below is the best P-value for a correlated SNP at the same locus from this study (if different from the reported SNP, r2 > 0.1). BFDP, Bayesian False Discovery Probability, calculated using prior = 1x10-5, maximum relative risk = 1.2. LD calculated based on European populations. *Combined OR. ** Homozygous in EUR populations, not present in data.

File Name: Supplementary Data 2
Description: Summary characteristics of each data set

File Name: Supplementary Data 3
Description: Exclusion of individual samples from each study owing to quality control. Samples were excluded due to call rate (<95% or failed genotyping), ethnicity (principal components analysis or other samples reported to be not of white, European descent), relatedness (any individuals found to be duplicated or related within or between data sets through IBS), sex discrepancies, or other reasons (controls with a first degree relative with CRC, low concordance of genotyping in duplicates, excess heterozygosity, or samples which have been subsequently withdrawn from a study). SNPs were filtered on genotype call rate > 0.05, differential case-control missingness > 10-5, info score > 0.8, minor allele frequency > 0.005, Hardy-Weinberg Equilibrium < 10-5. Individual level genotyping data were not available from the Finnish cohort.

File Name: Supplementary Data 4
Description: Conditional analysis on CRC SNPs previously identified in Asian populations. Shown are the SNPs found to be genome-wide significant in GWAS from Asian populations, along with their P-values in this meta-analysis. Where applicable, the top SNP in the region is shown. Population allele frequencies based on 1000 Genomes data. In the MAF (Minor allele frequency) column is shown the MAF for the respective population, with the minor allele. EAS: East Asian, EUR: European.

File Name: Supplementary Data 5
Description: Colorectal cancer risk loci shared with other cancers. Data were obtained from the NHGRI-EBI GWAS Catalog. Only variants with r2 > 0.1 with the CRC SNP are shown.

File Name: Supplementary Data 6
Description: Predicted disrupted transcription factor binding at colorectal cancer risk SNPs, as determined by motifbreakR. Also shown are the transcription factors bound at these sites in LoVo, HT29 and ENCODE cell lines.

File Name: Supplementary Data 7
Description: Results from the cis-eQTL analysis at new and established CRC risk loci. All nominally significant (P < 0.05) SNP-gene pairs are shown for both the INTERMPHEN data set and the GTEx transverse colon dataset for new and previously-reported.

File Name: Supplementary Data 8
Description: SMR analysis showing GWAS loci with significant associations with gene expression. We

show the results for the INTERMPHEN and GTEx transverse colon datasets based on SMR analysis of eQTLs with P-values <5x10 -8. Bonferroni-corrected transcriptome-level SMR significance is P = 8.6x10-5.

File Name: Supplementary Data 9
Description: Integration of expression quantitative trait locus, histone modification, and promoter capture Hi-C data at all colorectal cancer risk loci to identify candidate target genes.

File Name: Supplementary Data 10
Description: Gene set enrichment analysis, showing enriched pathways.

File Name: Supplementary Data 11
Description: Data-driven Expression-Prioritized Integration for Complex Traits gene prioritisation and tissue enrichment of colorectal cancer risk SNPs.

File Name: Supplementary Data 12
Description: LD score regression determining genetic correlation between colorectal cancer and selected other traits.

File Name: Supplementary Data 13
Description: CRC SNPs identified in contemporaneous study.

File Name: Supplementary Data 14
Description: Fidelity of imputation. Imputed genotypes were compared to sequencing derived genotypes on 201 samples with both WGS and Omni-Express arrays at 51 SNPS tested.
