# Supplementary material for: Association analyses identify 31 new risk loci for colorectal cancer susceptibility
Source: Nat Commun. 2019 May 14;10:2154. doi: 10.1038/s41467-019-09775-w (PMC6517433; doi:10.1038/s41467-019-09775-w)
Supplement: Supplementary file 18 — Reporting Summary [file 41467_2019_9775_MOESM18_ESM.pdf]

nature research

Corresponding author(s): Richard Houlston

Reporting Summary

Nature Research wishes to improve the reproducibility of the work that we publish. This form provides structure for consistency and transparency in reporting. For further information on Nature Research policies, see Authors & Referees and the Editorial Policy Checklist.

Statistical parameters

When statistical analyses are reported, confirm that the following items are present in the relevant location (e.g. figure legend, table legend, main text, or Methods section).

n/a	Confirmed	
☐	☒	The exact sample size (*n*) for each experimental group/condition, given as a discrete number and unit of measurement
☐	☒	An indication of whether measurements were taken from distinct samples or whether the same sample was measured repeatedly
☐	☒	The statistical test(s) used AND whether they are one- or two-sided<br>*Only common tests should be described solely by name; describe more complex techniques in the Methods section.*
☐	☒	A description of all covariates tested
☐	☒	A description of any assumptions or corrections, such as tests of normality and adjustment for multiple comparisons
☐	☒	A full description of the statistics including central tendency (e.g. means) or other basic estimates (e.g. regression coefficient) AND variation (e.g. standard deviation) or associated estimates of uncertainty (e.g. confidence intervals)
☐	☒	For null hypothesis testing, the test statistic (e.g. *F*, *t*, *r*) with confidence intervals, effect sizes, degrees of freedom and *P* value noted<br>*Give P values as exact values whenever suitable.*
☐	☒	For Bayesian analysis, information on the choice of priors and Markov chain Monte Carlo settings
☐	☒	For hierarchical and complex designs, identification of the appropriate level for tests and full reporting of outcomes
☐	☒	Estimates of effect sizes (e.g. Cohen's *d*, Pearson's *r*), indicating how they were calculated
☒	☐	Clearly defined error bars<br>*State explicitly what error bars represent (e.g. SD, SE, CI)*

Our web collection on statistics for biologists may be useful.

Software and code

Policy information about availability of computer code

Data collection	GenomeStudio
Data analysis	GWAS: SHAPEIT v2.83781, IMPUTE v2.3.282, SNPTEST v2.5.285, meta v1.7, SNP2HLA, GCTA v1.91, plink v1.90<br>eQTL: RNA-SeQC v1.1.8, edgeR, FastQTL, metaTissue, SMR<br>Additional analyses: EPIGWAS, motifbreakR, ldsc, LDAK, InBioMap, GenGen, genesis<br>All software is publicly available

For manuscripts utilizing custom algorithms or software that are central to the research but not yet described in published literature, software must be made available to editors/reviewers upon request. We strongly encourage code deposition in a community repository (e.g. GitHub). See the Nature Research guidelines for submitting code & software for further information.

Data

Policy information about availability of data

All manuscripts must include a data availability statement. This statement should provide the following information, where applicable:

- Accession codes, unique identifiers, or web links for publicly available datasets
- A list of figures that have associated raw data
- A description of any restrictions on data availability

The SCOT data can be requested through the TransSCOT committee according to the ethical permissions obtained as part of the clinical trial approval (https://www.oncology.ox.ac.uk/trial/scot). The PRACTICAL and BCAC consortium control data is available through the respective Data Access Coordination Committees (http://practical.icr.ac.uk and http://bcac.ccge.medschl.cam.ac.uk/) and the Heinz Nixdorf Recall Study control data can be requested through https://www.uni-due.de/recall-studie/die-studien/hnr/. UK Biobank data can be obtained through http://www.ukbiobank.ac.uk/. The Colon Cancer Family Registry data can be obtained through http://coloncfr.org/. Finnish cohort samples can be requested from THL Biobank https://thl.fi/en/web/thl-biobank. Hi-C data have been deposited in EGA under accession number EGAS00001002614. The accession number for the ChIP-seq data reported in this paper is EGAS00001002414. The remaining data are contained within the Supplementary Files or available from the authors upon reasonable request.

Field-specific reporting

Please select the best fit for your research. If you are not sure, read the appropriate sections before making your selection.

☒ Life sciences ☐ Behavioural & social sciences ☐ Ecological, evolutionary & environmental sciences

For a reference copy of the document with all sections, see nature.com/authors/policies/ReportingSummary-flat.pdf

Life sciences study design

All studies must disclose on these points even when the disclosure is negative.

Sample size	Observational study, so results based on all available data. This new study includes data on over 73,500 new samples.
Data exclusions	Data were quality controlled using established measures. Specifically, individuals with low SNP call rate (<95%), sex discrepancies and individuals evaluated to be of non-European ancestry (using the HapMap version 2 CEU, JPT/CHB and YRI populations as a reference) were excluded. For apparent first-degree relative pairs, we excluded the control from a case-control pair; otherwise, we excluded the individual with the lower call rate.<br>SNPs were excluded if call rate <95%, MAF < 0.5%, significantly different call rate between cases and controls (P < 10–5), or displaying significant deviation from Hardy-Weinberg equilibrium (P < 10–5).
Replication	Observational study, so results based on all available data. Results were meta-analysed with previous studies.
Randomization	Observational study, so randomisation not relevant. Sample recruitment based on presence or absence of colorectal cancer
Blinding	Population based study, so blinding not relevant.

Reporting for specific materials, systems and methods

Materials & experimental systems

n/a	Involved in the study
☒	☐ Unique biological materials
☒	☐ Antibodies
☒	☐ Eukaryotic cell lines
☒	☐ Palaeontology
☒	☐ Animals and other organisms
☐	☒ Human research participants

Methods

n/a	Involved in the study
☒	☐ ChIP-seq
☒	☐ Flow cytometry
☒	☐ MRI-based neuroimaging

Human research participants

Policy information about studies involving human research participants

Population characteristics	Samples were of Northern European Ancestry.

Recruitment

Cases were recruited based on presence of colorectal cancer through clinics, diagnosed with the following codes: ICD-9 153, 154; ICD10 C18.9, C19, C20. Controls were healthy, unrelated individuals with no cancer diagnosis.
